# Tissue‐Resident Macrophage in Inflammation and Cancer

**DOI:** 10.1002/mco2.70970

**Published:** 2026-09-11

**Authors:** Siyuan Huang, Jiangbo Fan, Wenyi Liu, Di Liu, Rui Wang, Yuanyang Wang, Jianhui Sun, Huacai Zhang, Qingli Cai, Zhe Xu, Fang Xu, Jianxin Jiang, Zhen Wang, Ling Zeng

**Affiliations:** ^1^ State Key Laboratory of Trauma and Chemical Poisoning Daping Hospital Army Medical University Chongqing China; ^2^ Department of Intensive Care Unit Daping Hospital, State Key Laboratory of Trauma and Chemical Poisoning, Army Medical University Chongqing China; ^3^ Department of Critical Care Medicine the First Affiliated Hospital of Chongqing Medical University Chongqing China; ^4^ Department of Emergency the Affiliated Hospital of Guizhou Medical University Guizhou Medical University Guiyang China

**Keywords:** immunotherapy, inflammation, macrophage plasticity, sepsis, tissue‐resident macrophages, tumor‐associated macrophages

## Abstract

Tissue‐resident macrophages (TRMs) are long‐lived immune cells strategically distributed across organs, where their functional plasticity enables both homeostatic maintenance and pathological dysfunction. This review provides a comprehensive analysis of TRM biology in inflammation and cancer. We first delineate the heterogeneity and developmental origins of TRM subsets across organs, highlighting how organ‐specific niche signals create distinct vulnerability patterns. We then dissect macrophage dysfunction during sepsis, tracing their trajectory from protective anti‐infectious immunity through critical tipping points to immunoparalysis. Next, we examine additional inflammatory contexts, including sterile inflammation, autoimmune inflammation, Type 2 inflammation, and metabolic inflammation, each revealing distinct facets of macrophage functional plasticity. In parallel, we examine how the tumor microenvironment chronically co‐opts TRMs, transforming them into tumor‐associated macrophages that promote angiogenesis, suppress antitumor immunity, facilitate metastasis, and support cancer stem cells (CSCs). Then, a systematic cross‐disease comparison reveals shared mechanisms that underlie the opposing functional outputs of macrophages in inflammation and cancer. Finally, we evaluate emerging therapeutic strategies and propose a paradigm shift from functional blockade toward precise recalibration of macrophage function to restore immune balance across both disease categories. Together, this review highlights the therapeutic potential of targeting shared macrophage regulatory nodes to restore immune balance across inflammation and cancer.

## Graphical abstract

Opposing macrophage functions in inflammation and cancer converge on shared mechanistic nodes, informing cross‐disease therapeutic strategies.

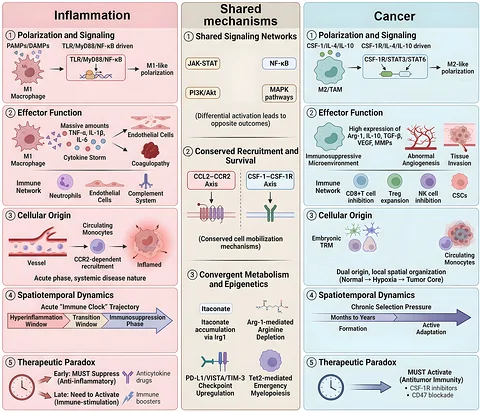

## 1 Introduction

Tissue‐resident macrophages (TRMs) are long‐lived innate immune cells strategically distributed across organs. Originating primarily from embryonic precursors and maintained through local self‐renewal, these cells serve as master regulators of tissue development, homeostasis, and repair [1]. Their functional identity is intricately shaped by tissue‐specific niches, giving rise to highly specialized roles. This profound adaptability, termed plasticity, enables TRMs to mount appropriately calibrated responses to diverse environmental cues, positioning them as indispensable first responders to infection, injury, and cellular stress [2].

However, this very plasticity renders TRMs a double‐edged sword. When their regulatory networks become dysregulated, these guardians of tissue health can pivot into central drivers of pathology. This Janus‐faced nature is starkly illustrated in the context of acute systemic inflammatory diseases, among which sepsis stands as the most devastating and well‐characterized example [3]. Sepsis remains a global health care issue, defined by dysregulated host responses to infection that culminate in life‐threatening organ dysfunction. With an estimated 49 million incident cases and 13 million attributable deaths annually worldwide, it imposes a heavy and persistent burden on health care systems globally [4]. Its high incidence and mortality rates, compounded by rising antimicrobial resistance and frequent delays in diagnosis, underscore the urgency of redefining therapeutic strategies through a deeper mechanistic understanding of the immune dysregulation that underlies this syndrome [5]. At the epicenter of this dysregulation lie macrophages, cells whose functional plasticity profoundly shapes the trajectory of sepsis [6]. During early infection, Toll‐like receptor (TLR)‐mediated recognition of pathogen‐associated molecular patterns (PAMPs) triggers essential bactericidal responses, including the production of proinflammatory cytokines and the execution of phagocytic clearance [7]. Paradoxically, these same defense mechanisms become maladaptive when sustained, precipitating inflammasome‐driven pyroptosis that amplifies tissue injury and propagates organ damage [8]. As sepsis evolves, metabolic exhaustion and epigenetic reprogramming conspire to convert macrophages into profoundly immunosuppressive entities [9, 10]. This functional conversion creates a permissive environment for secondary infections, characterized by elevated expression of negative costimulatory molecules [11], progressive mitochondrial dysfunction [12], and a senescence‐associated secretory phenotype [13]. This progression from hyperinflammation to immunoparalysis, termed the “persistent inflammation–immunosuppression paradox,” epitomizes the formidable challenge posed by dysregulated macrophage plasticity in acute inflammatory diseases [14].

Beyond acute infection‐driven inflammation, however, TRMs are also central to a much broader spectrum of inflammatory diseases that collectively impose a staggering global health burden. Sterile inflammation underlies conditions such as ischemia–reperfusion injury, gout, and atherosclerosis [15, 16]; autoimmune inflammation drives rheumatoid arthritis (RA), systemic lupus erythematosus, and multiple sclerosis [17]; Type 2 inflammation is the core pathology of allergic asthma and atopic dermatitis [18]; and metabolic inflammation fuels the progression of obesity, nonalcoholic steatohepatitis (NASH), and Type 2 diabetes [19]. Despite advances in anticytokine biologics and disease‐modifying antirheumatic drugs, many of these conditions remain poorly controlled, with high rates of treatment resistance, relapse, and long‐term disability. Current therapies largely target terminal effector pathways rather than the underlying cellular dysregulation, and none adequately address the functional plasticity of macrophages that drives disease chronicity and progression [20, 21]. Given that macrophages serve as master integrators of inflammatory signals across all these contexts, strategies that recalibrate macrophage function, rather than simply blocking individual cytokines, hold promise for achieving more durable and broadly applicable therapeutic effects. Understanding how macrophage plasticity is deployed differently across sterile, autoimmune, Type 2, and metabolic inflammation is therefore essential for identifying shared regulatory nodes that can be targeted to restore immune balance.

Strikingly, this dynamic spectrum of macrophage dysfunction is not unique to inflammation. In the ostensibly disparate realm of cancer, a remarkably parallel process of macrophage subversion unfolds. Within the tumor microenvironment, TRMs and their monocyte‐derived counterparts are co‐opted by malignant cells. Educated by factors such as hypoxia, lactate, and tumor‐derived cytokines including colony‑stimulating factor 1 (CSF‐1) and TGF‐β, these cells adopt a chronically reprogrammed, protumoral phenotype [22]. Tumor‐associated macrophages (TAMs) foster angiogenesis, suppress antitumor immunity, facilitate invasion and metastasis, and support the maintenance of CSCs, thereby serving as faithful accomplices to tumor progression [23]. Although the tempo and triggers differ, acute pathogen‐driven inflammation versus chronic tumor‐driven education, the underlying mechanisms of macrophage reprogramming in these two disease contexts are profoundly convergent. Dysregulated metabolic pathways, pathogenic epigenetic imprinting, and the upregulation of immune checkpoints such as PD‐L1 are features shared by both the septic immunoparalytic macrophage and the immunosuppressive TAM [24, 25]. This mechanistic convergence suggests that insights gained from studying TRM dysfunction in one disease context can profoundly illuminate the other. The inflammatory macrophage, in essence, provides an accelerated and temporally compressed model of the macrophage dysfunction that the tumor microenvironment achieves through sustained chronic signaling.

This functional duality raises a critical question for therapeutic targeting: do macrophages serve primarily as protectors or as perpetrators of pathology in these diseases? The therapeutic challenge is further compounded by an apparent paradox. In sepsis and other inflammatory conditions, early interventions must temper the hyperinflammatory response without crippling host defense, while late‐stage strategies must reverse established immunoparalysis without provoking a resurgence of inflammation [26]. In cancer, the goal is precisely the opposite: to reignite the suppressed antitumor functions of TAMs and dismantle the immunosuppressive niche they maintain [27]. Adding further complexity, emerging single‐cell and spatial transcriptomic analyses have revealed striking organ‐specific and disease‐specific macrophage phenotypes [28], from ferroptosis of alveolar macrophages (AM) in sepsis‐induced acute respiratory distress syndrome [29] to the SPP1^hi^ proangiogenic TAM subset in colorectal cancer [30]. Such heterogeneity demands precision therapeutic approaches that extend beyond simplistic “activate” or “inhibit” paradigms toward the spatiotemporally precise recalibration of macrophage behavior.

In this review, we provide a comprehensive analysis of the roles of TRMs in inflammatory diseases and cancer. We first delineate the heterogeneity and developmental origins of distinct TRM subsets across major organs, examining how niche‐specific imprinting shapes their homeostatic functions and pathological responses. We then systematically track the dynamic trajectory of macrophage dysfunction during sepsis progression, from the initial hyperinflammatory phase through the critical tipping point of inflammasome‐driven tissue injury to the late‐stage immunosuppressive phase of immunoparalysis. We then extend our analysis to additional inflammatory contexts, including sterile inflammation, autoimmune inflammation, Type 2 inflammation, and metabolic inflammation, each illustrating distinct paradigms of macrophage‐driven pathology. The discussion subsequently transitions to the hijacked roles of macrophages in the tumor microenvironment, dissecting their multifaceted contributions to immune suppression, angiogenesis, metastasis, and CSC support. A dedicated cross‐disease comparison synthesizes the core functional differences and the underlying mechanistic similarities, highlighting the value of this comparative perspective for therapeutic discovery. Finally, we evaluate emerging therapeutic strategies that target macrophage plasticity, assessing their potential to restore immune balance across both inflammatory and neoplastic diseases.

## 2 TRM in Different Organs

**FIGURE 1:**
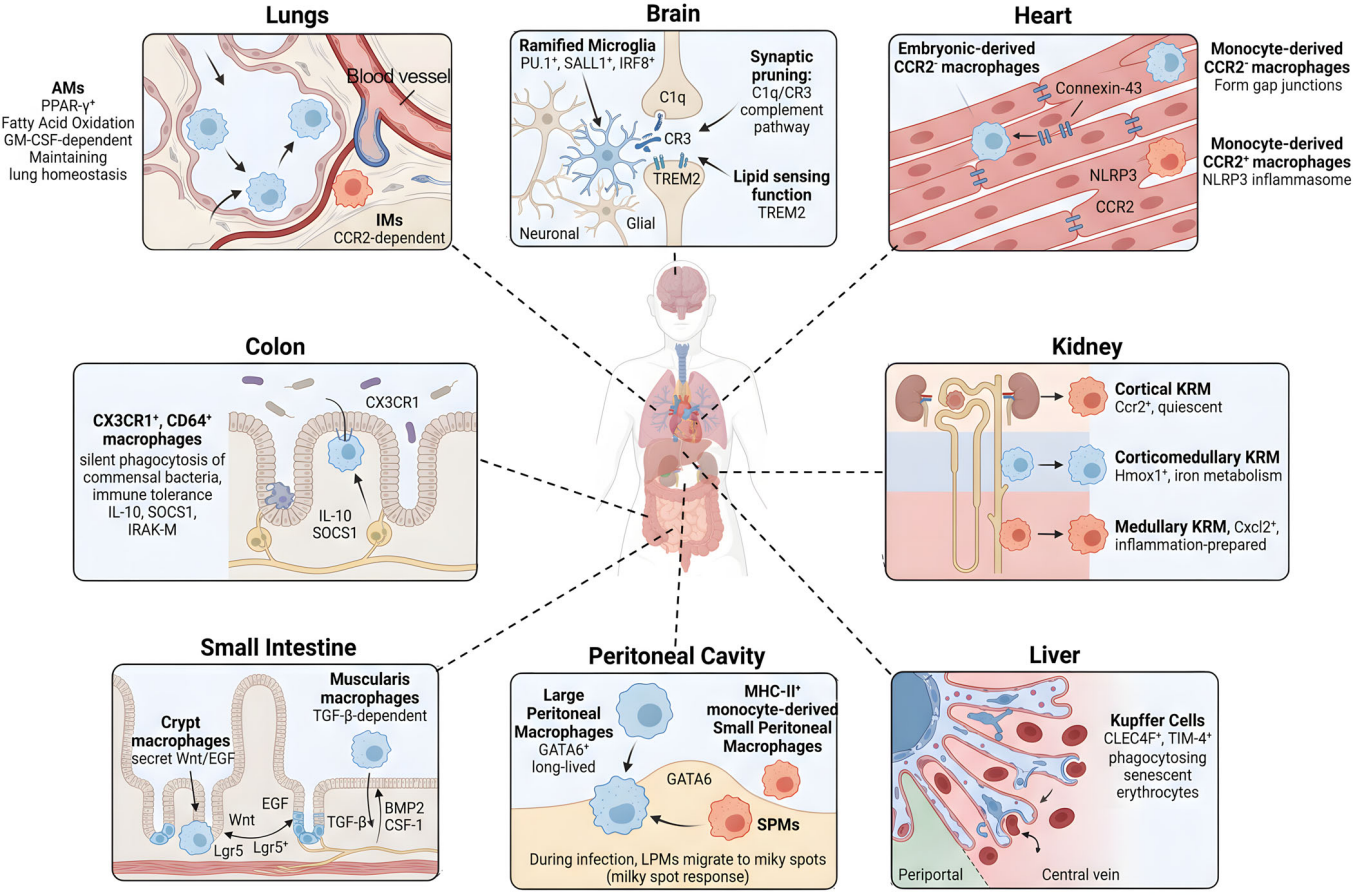
Heterogeneity and organ‐specific specialization of tissue‐resident macrophages. This schematic illustrates the distinct origins, phenotypic markers, and functional adaptations of TRMs across eight major anatomical sites. As depicted from top to bottom, the lungs harbor PPAR‐γ^+^, GM‐CSF‐dependent alveolar macrophages (AMs) that maintain surfactant homeostasis via fatty acid oxidation, alongside CCR2‐dependent interstitial macrophages (IMs). The brain contains ramified microglia (PU.1^+^, SALL1^+^, IRF8^+^) that prune synapses through the C1q/CR3 complement pathway and sense lipids via TREM2. The heart comprises embryo‐derived CCR2^−^ macrophages forming Connexin‐43 gap junctions and monocyte‐derived CCR2^+^ macrophages expressing the NLRP3 inflammasome. In the kidney, cortical, corticomedullary, and medullary kidney‐resident macrophages (KRMs) are distributed along an osmotic gradient, exhibiting quiescent (Ccr2^+^), iron‐handling (Hmox1^+^), and inflammation‐prepared (Cxcl2^+^) phenotypes, respectively. In the colon, CX3CR1^+^, CD64^+^ macrophages mediate silent phagocytosis of commensal bacteria and maintain immune tolerance through IL‐10, SOCS1, and IRAK‐M. In the small intestine, distinct subsets include crypt macrophages that support Lgr5^+^ stem cells via Wnt/EGF secretion, and muscularis macrophages that interact with enteric nerves through TGF‐β, BMP2, and CSF‐1. The peritoneal cavity harbors GATA6^+^ long‐lived large peritoneal macrophages (LPMs) and MHC‐II^+^ monocyte‐derived small peritoneal macrophages (SPMs), with LPMs undergoing a milky spot response during infection. Finally, in the liver, CLEC4F^+^, TIM‐4^+^ Kupffer cells exhibit spatial zonation and specialize in the phagocytosis of senescent erythrocytes. Together, these diverse populations illustrate the developmental dichotomy and niche‐driven functional specialization that define TRM biology across distinct organ systems. (Drafted by NanoBanana Pro 4K and subsequently manually redrawn and finalized by the author using BioRender.)

Macrophages represent a functionally and ontogenetically heterogeneous population that is central to tissue surveillance and inflammatory regulation [31]. Current classification frameworks emphasize their developmental dichotomy: TRMs originate from embryonic precursors and maintain tissue homeostasis through local self‐renewal, whereas circulatory monocyte‐derived macrophages arise from bone marrow progenitors and infiltrate tissues during inflammatory challenges. This ontogenetic divide underpins their functional specialization‐resident populations, which exhibit tissue‐adapted metabolic profiles and express unique markers, whereas recruited macrophages display heightened plasticity to respond dynamically to microenvironmental cues [32]. Different tissues can harbor distinct subsets of macrophages, including monocyte‐derived macrophages, with conserved origins and life cycles. The parameters that dictate the carrying capacity of these tissue‐specific macrophage subsets are yet to be fully defined (Figure 1).

### 2.1 Lung

The lung harbors a sophisticated macrophage network adapted to the organ's unique anatomical and functional demands. As the primary interface for gas exchange, the lung is continuously exposed to inhaled particulates, airborne pathogens, and fluctuating oxygen tensions. This distinctive environment necessitates a multilayered macrophage system capable of rapid pathogen clearance while minimizing inflammatory injury to the delicate alveolar–capillary interface. The pulmonary macrophage compartment comprises two principal populations with fundamentally distinct developmental origins and anatomical niches: AMs residing within the airspace lumen, and interstitial macrophages (IMs) occupying the bronchovascular interstitium [33].

AMs are the dominant immune cell population of the airspace, accounting for approximately 90–95% of resident immune cells in the alveolar lumen under homeostatic conditions. Their developmental origins trace to yolk sac erythromyeloid progenitors that seed the developing lung during embryogenesis (E10.5‐11.5 in mice). Following birth, AMs are maintained predominantly through local self‐renewal rather than replenishment by circulating monocytes. This self‐sustaining capacity is driven by a specialized transcriptional program centered on the GM‐CSF/PU.1/PPAR‐γ axis [34, 35]. TGF‐βR signaling reinforces AM identity by cooperatively inducing PPAR‐γ expression [36]. PPAR‐γ serves as the master transcriptional regulator of AM differentiation and maintenance, orchestrating the expression of genes involved in lipid uptake, surfactant catabolism, and efferocytosis. The dependence on GM‐CSF signaling is underscored by the complete absence of mature AMs in Csf2‐deficient mice, which develop pulmonary alveolar proteinosis due to defective surfactant clearance [37]. In humans, AMs typically coexpress HLA‐DR, CD43, CD169, CD206, CD11b, CD141, CD64, and low levels of CD14. In mice, they are reliably identified by the surface phenotype CD11c^hi^Siglec‐F^hi^CD64^hi^F4/80^lo^CD11b^lo^. Functionally, AMs are specialized for the clearance of pulmonary surfactant through the ATP‐binding cassette transporter ABCA3, as well as the phagocytosis and degradation of inhaled particles and pathogens [38]. Their unique metabolic wiring, characterized by a constitutive dependence on fatty acid oxidation and PPAR‐γ‐driven lipid metabolism, enables sustained surfactant clearance while simultaneously constraining the rapidity with which they can shift metabolic programs in response to acute perturbations [39]. Recent single‐cell multiomics studies have revealed that Ams exhibit distinct metabolic and regulatory features that shape their responses in both inflammatory and neoplastic lung diseases. AMs rely on a unique regulatory network architecture involving the transcription factors PU.1 and CEBP/β, which constrains their inflammatory responses compared with recruited monocyte‐derived macrophages, serving as a molecular safeguard against excessive immunopathology [40]. In the pulmonary fibrosis to lung cancer transition, key profibrotic and protumor AM subsets, including SPP1^+^, TREM2^+^, and MARCO^+^ cells, have been identified [41]. The fibrotic microenvironment induces profound metabolic reprogramming, including the Warburg effect and lipid peroxidation, and stabilizes epigenetic memory through DNA methylation and histone modifications [42]. These AMs form self‐amplifying feedback loops with fibroblasts and epithelial cells through secreted factors and extracellular vesicles (EVs), driving the progression from chronic inflammation to malignancy [43]. This integrated multiomics perspective positions AMs as central hubs linking chronic lung inflammation to tumor development.

IMs populate the alveolar septum, bronchial submucosa, and perivascular spaces, where they constitute a phenotypically and functionally heterogeneous population distinct from their alveolar counterparts [44]. Unlike AMs, which are largely of embryonic origin, the IM compartment is continuously replenished by circulating monocytes in a CCR2‐dependent manner, although a subpopulation of embryo‐derived, self‐renewing IMs also persists [45, 46]. Two major IM subsets have been characterized based on surface marker expression and anatomical localization. Perivascular IMs, identified as CD206^hi^Lyve‐1^hi^MHC‐II^lo^CX3CR1^−^, are tightly associated with blood vessels and contribute to endothelial barrier maintenance through constitutive VEGF‐α signaling and PLAUR‐dependent mechanisms. Nerve‐associated interstitial phagocytes, defined as CD206^−^ Lyve‐1^lo^MHC‐II^hi^ CX3CR1^+^, reside in close proximity to peripheral nerves and participate in neuroimmune crosstalk [45]. In contrast to AMs, IMs express higher levels of pattern recognition receptors (PRRs) and costimulatory molecules, positioning them to respond rapidly to signals that breach or translocate across the alveolar–capillary barrier [47]. Their functional repertoire is correspondingly broader, encompassing antigen presentation, proinflammatory cytokine production, and extracellular matrix (ECM) remodeling.

The niche signals that shape lung TRM identity derive predominantly from the unique physicochemical environment of the pulmonary airspace and interstitium. AMs operate in an environment with minimal microbial colonization under homeostatic conditions, and their identity is driven by the lipid‐rich alveolar microenvironment, where surfactant components serve as both metabolic substrates and transcriptional regulators. Surfactant protein D, for instance, not only opsonizes pathogens but also modulates AM TLR responses, maintaining these cells in a state of controlled readiness that balances pathogen surveillance against inflammatory restraint [48]. IMs, by contrast, are shaped by signals from the vascular endothelium and the ECM, including VEGF, angiopoietins, and integrin‐mediated mechanical sensing [49]. These distinct niche inputs ensure that AMs and IMs each adopt a functional posture calibrated to their specific anatomical location: luminal AMs poised for rapid phagocytic clearance with minimal inflammatory perturbation, and interstitial IMs positioned for more robust innate immune activation at the vascular interface.

The developmental and functional division of labor between self‐sustaining luminal macrophages and monocyte‐replenished interstitial responders represents a core organizational principle of pulmonary macrophage biology. The embryo‐derived origin and PPAR‐γ‐dependent transcriptional program of AMs provide a relatively stable functional identity optimized for homeostatic surfactant metabolism and silent phagocytosis [50]. This specialization renders AMs dependent on local GM‐CSF signaling for their maintenance, a dependency that may constrain their capacity to adapt when the alveolar niche is disrupted. The monocyte‐dependent replenishment of IMs, by contrast, positions this population for greater functional responsiveness to systemic and local perturbations [51]. These intrinsic differences in developmental origin and niche dependence fundamentally shape how each population participates in inflammatory and neoplastic disease processes within the lung.

### 2.2 Heart

The heart harbors a diverse macrophage network that is integral to myocardial development, homeostasis, and the coordination of tissue responses to physiological stress. Cardiac macrophages operate within a sterile environment under continuous mechanical load, where they support critical functions including electrical conduction, coronary vascular integrity, and the clearance of senescent cardiomyocytes [52]. Single‐cell transcriptomic analyses have revealed two ontogenetically distinct macrophage populations within the murine heart: a predominant population of embryonic‐derived CCR2^−^ macrophages that are maintained through local self‐renewal, and a smaller population of bone marrow‐derived CCR2^+^ macrophages that arise from circulating monocytes and undergo gradual replacement over time [53].

The embryo‐derived CCR2^−^ population constitutes the principal cardiac macrophage subset under homeostatic conditions. These cells are seeded from yolk sac and fetal liver progenitors during embryonic development and persist throughout postnatal life through in situ proliferation. Their maintenance is independent of monocyte input, relying instead on signals from the local myocardial niche, including CSF‐1 produced by cardiomyocytes and fibroblasts [53]. CCR2^−^ cardiac macrophages uniformly express CD64 and MerTK and can be further subdivided by major histocompatibility complex (MHC)‐II and CD206 expression levels into functionally distinct subsets. CCR2^−^MHC‐II^lo^ macrophages express high levels of CD206, TIMD4, and LYVE1 and are specialized for homeostatic clearance of apoptotic cells and tissue surveillance. CCR2^−^MHC‐II^hi^ macrophages also express CD64 and MerTK, but are distinguished by higher CX3CR1 and intermediate CD206 expression, lower TIMD4 and LYVE1, and a transcriptional program enriched for antigen presentation and chemokine responsiveness. Functionally, they are specialized for homeostatic maintenance rather than inflammatory activation. A hallmark of their homeostatic role is the facilitation of cardiomyocyte calcium handling through connexin‐43 gap junctions formed at macrophage–cardiomyocyte contact sites, a process that directly contributes to normal electromechanical coupling [54]. CCR2^−^ macrophages also perform continuous immune surveillance through MerTK‐mediated efferocytosis, efficiently clearing apoptotic cardiomyocytes and cellular debris without triggering inflammation [55]. This silent phagocytosis is essential for maintaining tissue health in an organ with limited regenerative capacity and a high metabolic demand.

The metabolic specialization of CCR2^−^ cardiac macrophages reflects their homeostatic functional posture. These cells preferentially utilize oxidative phosphorylation, mainly fatty acid oxidation, which provides the steady energy supply required for sustained efferocytosis and tissue surveillance. This metabolic configuration simultaneously suppresses the expression of proinflammatory cytokines, coupling metabolic state to functional output [56]. The dependence on oxidative phosphorylation parallels that of AMs but is tailored to the distinct demands of myocardial tissue, where continuous contractile activity requires macrophages to operate without perturbing the electrical and mechanical function of adjacent cardiomyocytes.

By contrast, the CCR2^+^ macrophage population is derived from circulating Ly6C^hi^ monocytes recruited to the heart in a CCR2‐dependent manner. Under homeostatic conditions, these cells are present at low frequencies and display a gene expression profile enriched for proinflammatory pathways, including NLRP3 inflammasome components and genes encoding CXCL chemokines [57]. GM‐CSF signaling drives the expression of the proinflammatory chemokine CCL17 through cooperative activation of STAT5 and canonical NF‐κB in CCR2^+^ macrophages recruited to the injured myocardium [58]. Additionally, TREM1/3‐dependent signaling promotes CCL3 production by these cells [59]. These pathways position CCR2^+^ macrophages for more rapid inflammatory responses upon challenge, while their dependence on continuous recruitment renders their population size tightly linked to monocyte influx dynamics.

The functional organization of cardiac macrophages extends beyond the CCR2^−^/CCR2^+^ dichotomy to encompass anatomically defined specializations. Single‐cell studies have further resolved the heterogeneity within the CCR2^−^‐resident macrophage pool. Beyond the MHC‐II^lo^ and MHC‐II^hi^ subsets, a distinct TIMD4^hi^MHC‐II^hi^ double‐positive population has been identified and validated through lineage tracing as being maintained independently of monocyte input. This population is characterized by the expression of Retnla and has been shown to play a protective role in limiting adverse cardiac remodeling [60]. Under pathological conditions, additional resident macrophage states have been identified, including TREM2^hi^‐resident macrophages that internalize dysfunctional mitochondria extruded by cardiomyocytes during septic cardiomyopathy [61], and VSIG4^+^‐resident macrophages that modulate postinfarction inflammation through the recruitment of CCR5^+^ regulatory T cells [62]. A CD74^+^ cardiac macrophage subset has been identified as a key mediator of trastuzumab‐induced cardiotoxicity, driving myocardial inflammation and apoptosis through CD74/STAT1 signaling [63]. In experimental autoimmune myocarditis, rapamycin was shown to target Cxcl9^+^ pathogenic macrophages via the mTORC1‐C/EBPβ axis, revealing a therapeutically tractable inflammatory subset [64].

The developmental and functional architecture of the cardiac macrophage network has several innate properties that carry implications for how this network responds to pathological conditions. The embryonic origin of the CCR2^−^ population provides a stable, long‐lived cellular foundation optimized for homeostatic support functions, but its limited capacity for rapid expansion may constrain the immediate availability of effector cells during acute challenges. The monocyte‐derived CCR2^+^ population provides a reservoir of inflammatory capacity that can be rapidly mobilized, but its recruitment and activation must be tightly regulated to prevent collateral injury to the nonregenerative myocardium. The anatomical specialization of macrophages within the conduction system, in particular, renders certain cardiac functions uniquely vulnerable to perturbations that disrupt the macrophage–cardiomyocyte interactions upon which normal electrophysiology depends. These organizing principles of cardiac macrophage biology establish the cellular framework within which acute inflammatory and chronic disease processes each operate.

### 2.3 Brain

Microglia are the resident macrophages of the central nervous system (CNS) and represent a unique population within the mononuclear phagocyte system. Unlike peripheral tissue macrophage populations that reside within stromal or epithelial environments, microglia occupy the neural parenchyma, a compartment that is anatomically sequestered from the systemic circulation by the blood–brain barrier and characterized by a distinct extracellular milieu dominated by neuronal and glial signals [65]. This specialized niche imposes unique functional demands on microglia and fundamentally shapes their developmental programming, metabolic configuration, and homeostatic effector functions.

Microglia originate exclusively from yolk sac erythromyeloid progenitors that colonize the developing brain during early embryogenesis, prior to the formation of the blood–brain barrier. In mice, this colonization occurs around embryonic Day 9.5, and the population is subsequently maintained throughout postnatal life through local self‐renewal without contribution from circulating monocytes under physiological conditions [66]. This embryonic origin is shared with other long‐lived TRM populations, such as AMs and Kupffer cells, and distinguishes microglia from monocyte‐derived macrophage populations that undergo continuous replacement from the bone marrow. The self‐renewal capacity of microglia is driven by CSF‐1R signaling, with its ligands CSF‐1 and interleukin (IL)‐34 provided by astrocytes and neurons, respectively [67]. The dependence on IL‐34 is a distinctive feature of the CNS niche, reflecting the regional specialization of macrophage trophic support across tissues [68].

The transcriptional identity of microglia is established by a core regulatory network centered on PU.1, IRF8, and the microglia‐specific transcription factor SALL1. PU.1 serves as the master lineage‐determining transcription factor required for the specification and maintenance of all macrophage populations [69]. SALL1 acts downstream of TGF‐β signaling to enforce the microglia‐specific gene expression program while simultaneously repressing genes associated with peripheral macrophage identity, thereby maintaining microglial uniqueness within the CNS environment [70]. IRF8 contributes to the regulation of microglial activation thresholds, modulating responses to environmental stimuli while preserving homeostatic functions [71]. This transcriptional framework is complemented by the obligate requirement for TGF‐β signaling, which is constitutively provided by the CNS microenvironment and is essential for maintaining the ramified morphology, low proliferative rate, and attenuated inflammatory responsiveness that characterize homeostatic microglia [72, 73].

The homeostatic functions of microglia are shaped by their continuous physical interaction with neural elements. Microglia extend highly ramified processes that dynamically survey the surrounding neuropil, making transient contacts with neuronal synapses, dendritic spines, and astrocytic endfeet [74, 75]. Through complement‐dependent mechanisms, microglia selectively prune redundant or weakened synapses during postnatal development and throughout adulthood. Synapses tagged with the classical complement component C1q are recognized by the microglial complement receptor CR3, triggering localized phagocytosis of synaptic elements [76]. This pruning activity is critical for the refinement of neural circuits and is regulated by neuronal signals including CX3CL1, which is expressed on the neuronal surface and engages the microglial receptor CX3CR1 to restrain excessive synaptic elimination [77]. Conversely, microglia provide neurotrophic support through the secretion of brain‐derived neurotrophic factor, which promotes neuronal survival, synaptic plasticity, and cognitive function [78]. This balance between synaptic removal and neurotrophic support represents a core homeostatic function of microglia that is continuously calibrated by the neural microenvironment.

The metabolic organization of microglia is closely coupled to their surveillance and housekeeping functions. Under homeostatic conditions, microglia rely predominantly on oxidative phosphorylation for ATP generation. This oxidative metabolic state is sustained by lactate provided by astrocytes through the astrocyte‐neuron lactate shuttle, establishing a metabolic coupling between glial populations that maintains microglia in a relatively quiescent functional posture [79]. TREM2 serves as a critical lipid sensor that links extracellular lipid signals to intracellular metabolic programs, regulating the expression of genes involved in lipid catabolism and oxidative phosphorylation [80]. The TREM2 signaling pathway is particularly important for microglial responses to membrane debris and apoptotic cells, allowing microglia to adjust their metabolic output to meet the demands of phagocytic clearance without activating proinflammatory effector programs [81].

Despite their anatomical sequestration within the CNS, microglia share fundamental ontogenetic and functional features with TRM populations in peripheral organs. They derive from the same embryonic precursors as AMs, Kupffer cells, and other long‐lived TRMs, and employ common transcriptional regulators including PU.1, express a conserved repertoire of PRRs such as TLR4 and NLRP3, and deploy shared effector mechanisms including phagocytosis and cytokine production [82, 83]. However, CNS‐specific signals such as SALL1 and the TGF‐β‐rich neural environment impose a distinctive functional posture on microglia that suppresses peripheral macrophage features and enforces a neural‐adapted identity [70].

The developmental and functional architecture of microglia carries several properties that may help explain how the CNS macrophage compartment responds to pathological conditions. The yolk sac origin and exclusive dependence on local self‐renewal provide a stable substrate for lifelong surveillance but mean that microglia cannot draw on a mobilizable pool of monocyte‐derived reinforcements when acutely challenged, a constraint that may become particularly consequential if the blood–brain barrier is compromised. The metabolic dependence on astrocyte‐derived lactate and oxidative phosphorylation sustains continuous surveillance but is thought to limit the speed of metabolic reprogramming required for robust responses. The complement‐dependent synaptic pruning machinery that shapes neural circuitry during development is constitutively expressed and poised for reactivation, positioning synaptic integrity as a potential target of microglial dysfunction under both inflammatory and neoplastic conditions. These architectural features are therefore thought to define the repertoire of responses available to the CNS macrophage compartment when confronted with acute systemic inflammation or chronic disease.

### 2.4 Liver

The liver harbors the largest population of TRMs in the body, predominantly represented by Kupffer cells, which reside within the hepatic sinusoids and constitute approximately 80–90% of the total macrophage pool in mammals. Their strategic location at the interface between the portal and systemic circulations positions them as the first macrophage population to encounter gut‐derived microbial products, dietary antigens, and metabolic substrates. This unique anatomical placement, combined with the liver's central role in systemic metabolism and detoxification, has shaped KCs into a functionally specialized macrophage population whose homeostatic activities extend beyond immune surveillance to encompass core metabolic and regulatory functions.

Kupffer cells originate from yolk sac erythromyeloid progenitors that colonize the fetal liver during early gestation [84]. In mice, this seeding occurs around embryonic Day 10.5–12.5 [85], coincident with the onset of hepatic hematopoiesis. Following their initial establishment, KCs are maintained throughout postnatal and adult life primarily through local self‐renewal, with minimal contribution from circulating monocytes under homeostatic conditions [86]. This self‐renewal capacity is dependent on CSF‐1R signaling [87]. IL‐34, the second ligand for CSF‐1R, is constitutively expressed by hepatocytes and has been shown to regulate KC polarization toward an immunosuppressive phenotype through the PI3K/Akt pathway [88]. However, the relative contributions of IL‐34 and CSF‐1 to KC self‐renewal under homeostatic conditions remain to be fully defined. The transcription factor LXRα cooperates with PU.1 and SPIC to establish the KC‐specific gene expression program [89], which includes hallmark surface markers such as CLEC4F, TIM‐4, and CD163 in combination with the pan‐macrophage markers F4/80 and CD11b [90].

The homeostatic functions of KCs extend well beyond classical immune surveillance. Positioned within the sinusoidal lumen, KCs are continuously exposed to senescent erythrocytes, cellular debris, and immune complexes arriving from the systemic circulation. Their efficient phagocytic clearance of these materials through scavenger receptor‐mediated mechanisms, including SR‐A and MARCO, prevents the accumulation of proinflammatory degradation products and maintains hepatic tissue health [91, 92]. KCs also serve as critical regulators of hepatic lipid metabolism. Through the expression of PPARγ, KCs sense and catabolize fatty acids via β‐oxidation, a metabolic configuration that provides energy for their homeostatic functions while simultaneously preventing excessive lipid accumulation within the hepatic parenchyma [93]. This lipid‐handling capacity is coupled to systemic metabolic regulation through the secretion of factors that modulate hepatocyte lipoprotein synthesis and glucose metabolism. The close metabolic interdependence between KCs and hepatocytes establishes a functional axis in which macrophage lipid handling directly influences hepatic and systemic metabolic homeostasis.

A defining feature of KC biology that has emerged from recent spatial transcriptomic and imaging studies is their functional zonation along the portal‐to‐central axis of the hepatic lobule. KCs are not a uniform population but exhibit positional specialization that reflects the gradients of oxygen, nutrients, and microbial products that characterize the hepatic sinusoid. Periportal KCs, located in the proximal sinusoidal regions closest to the portal triad, are exposed to higher oxygen tensions and greater concentrations of gut‐derived microbial products [94]. Their transcriptional program is enriched for genes involved in pathogen recognition, cytokine production, and phagocytosis, reflecting their role as first‐line responders to materials arriving from the gut via the portal vein. Pericentral KCs, by contrast, reside in the low‐oxygen environment surrounding the central vein and express higher levels of scavenger receptors and genes associated with efferocytosis, consistent with their role in clearing cellular debris and aged erythrocytes that reach the distal sinusoid [95].

KCs are yolk sac‐derived and depend on local niche signals, establishing a bidirectional dependency with hepatocytes that amplifies the consequences of disruption. Beyond KCs, the liver also harbors liver capsular macrophages, which reside on the hepatic capsule and are replenished by blood monocytes, and lipid‐associated macrophages (LAMs), which localize near the bile ducts and may derive from peripheral monocytes while retaining self‐renewal capacity. Their central role in systemic iron and lipid metabolism means that KC dysfunction can propagate beyond the liver. Spatial zonation creates distinct populations that likely respond differentially to pathological signals: periportal KCs may be preferentially engaged during gut‐derived microbial translocation, while pericentral KCs may be more vulnerable to hypoxic stress. In the tumor microenvironment, these same populations are thought to be progressively subverted. These features define the repertoire of responses available to the hepatic macrophage compartment during acute inflammation and cancer.

### 2.5 Kidney

The kidney harbors a diverse network of TRMs that is distributed across its anatomically and functionally distinct compartments. Kidney‐resident macrophages (KRMs) operate across steep gradients of oxygen tension, osmolarity, and shear stress that characterize the renal cortex, outer medulla, and inner medulla. This environmental heterogeneity, combined with the kidney's dual role in filtration and systemic homeostasis, has shaped a macrophage network of considerable phenotypic diversity whose organizational principles are only beginning to be resolved through single‐cell genomics.

The developmental origins of KRMs reflect a mixed contribution from embryonic and hematopoietic sources. Fate‐mapping studies have demonstrated that a substantial fraction of KRMs originates from fetal liver progenitors that seed the developing kidney during late embryogenesis and persist into adulthood through local self‐renewal [84]. These embryo‐derived populations coexist with macrophages arising from circulating monocytes that are recruited in a CCR2‐dependent manner and progressively acquire tissue‐resident phenotypes under the influence of the renal microenvironment [96]. Single‐cell transcriptomic and fate‐mapping studies have revealed that CCR2^+^ monocyte‐derived KRMs are predominantly localized to the renal cortex, where their accumulation depends on CX3CR1–CX3CL1 signaling for differentiation and maintenance [96]. This dual‐origin composition distinguishes the kidney from organs such as the brain and liver, where the macrophage pool is dominated by embryo‐derived, self‐renewing populations, and more closely resembles the heart, where both sources contribute to the homeostatic macrophage network.

Single‐cell transcriptomic analyses have identified seven transcriptionally distinct KRM subpopulations under homeostatic conditions. One cluster, defined by Ccr2 and Ptprc (CD45) and localized predominantly to the renal cortex, exhibits few uniformly high transcripts, suggesting a quiescent surveillance state. A second cortical cluster expresses heat shock protein genes and the immediate early response factors Fos and Btg2, potentially reflecting homeostatic priming. A third cluster, localized to the corticomedullary junction, is defined by high expression of Hmox1, ferroportin (Slc40a1), and ferritin chains, indicating a specialized role in heme and iron handling. A fourth cluster, enriched in the renal medulla, expresses the chemokines Cxcl2, Ccl3, and Ccl4 together with CD14, positioning it for rapid innate immune responses and inflammatory cell recruitment. The remaining clusters, distributed across cortical and corticomedullary regions, are enriched for tissue remodeling transcripts (Pf4, Mrc1, Stab1), monocyte‐recruitment factors (Vim, Ccl2, Tnip3), and Type I interferon (IFN) response genes (Isg15, Ifit2, Ifit3), respectively. This diversity spans iron recycling, immune surveillance, tissue remodeling, and antiviral defense [97].

The niche signals that sustain KRM identity and spatial organization are derived from the unique physicochemical properties of the renal microenvironment. Tubular epithelial cells are an important source of CSF‐1 and CX3CL1, which support KRM maintenance and recruitment [98, 99]. The hypertonic medullary interstitium imposes osmotic stress that requires cellular adaptation through NFAT5, a transcription factor that induces osmoprotective gene programs [100]. Macrophages in the kidney can express HIF‐1α under hypoxic conditions, coinhibitory molecule VISTA combined with the oxygen gradient across the corticomedullary axis is thought to contribute to the phenotypic differences between cortical and medullary KRMs [101].

The organizational features of the kidney macrophage network collectively establish a cellular architecture in which anatomically segregated populations serve distinct surveillance and homeostatic functions. The medullary population stands at the interface between the urinary space and the systemic circulation, constitutively equipped for rapid chemokine‐driven responses. The cortical populations maintain tubular and glomerular integrity through continuous immune complex clearance and trophic support. These spatially organized, functionally specialized macrophage subsets define the cellular framework on which kidney macrophage responses to systemic and local pathological challenges are constructed.

### 2.6 Colon

The colon is the organ with the highest microbial density in the human body. Its mucosal surface constitutes an immense interface, constantly challenged by dietary antigens and trillions of commensal microorganisms. Unlike the lung and liver, which primarily confront periodically encountered pathogens, colonic TRMs must strike an exquisite balance between recognizing and eliminating potential pathogens and maintaining immune tolerance toward harmless commensals and food antigens. Shaped by this unique niche, colonic TRMs have become one of the most profoundly adapted and functionally specialized macrophage populations in the body, and their dysfunction is a core driver of inflammatory disease and colorectal cancer.

The developmental origins of colonic macrophages display a complex hierarchy. Traditionally, intestinal macrophages were viewed as a short‐lived population continuously replenished by circulating classical Ly6C^hi^ monocytes, depending on persistent recruitment via the CCL2–CCR2 chemokine axis [102, 103]. However, subsequent lineage‐tracing experiments demonstrate that a subset of long‐lived, self‐maintaining macrophages also resides in the colon. This population, strategically positioned near blood vessels, the submucosal and myenteric plexus, arises from both embryonic precursors and adult bone marrow‐derived monocytes, and persists into adulthood [104]. Mature colon‐resident macrophages can be unequivocally identified as a CD11bʰ^i^ CD64ʰ^i^ population within the CD45^+^ immune cell compartment, and they highly express CX3CR1. For analyzing the lamina propria (LP), a lineage cocktail (e.g., CD3, CD19, NKp46) should be used to exclude lymphocytes prior to gating on Lin^−^ CD11b^+^ cells to define the mature CD64^+^ CX3CR1^+^ macrophages. To further distinguish long‐lived, self‐maintaining TRMs from monocyte‐derived populations, markers such as TIMD4 and CD4 are specifically upregulated by these resident cells [105]. In terms of specific subsets, CD169 identifies perivascular macrophages near epithelial crypts, F11r/CD321 marks macrophages associated with the enteric nervous system in the muscularis externa, LYVE1 defines perivascular macrophages abundant in the serosa [106], and CD206 is upregulated by mature macrophages, while Ly6C (negative) and MHC‐II (positive) indicate the final differentiation state of mature macrophages, as opposed to infiltrating monocytes [107].

Instructed by niche signals, mature, stable colonic TRMs display a unique phenotype of inflammatory anergy. They possess potent phagocytic capacity but weak bactericidal activity, efficiently expressing scavenger receptors such as CD36 and MSR1 to rapidly recognize and ingest bacteria that breach the barrier. Yet even upon bacterial engulfment they avoid mounting a robust oxidative burst, thereby preventing widespread tissue inflammation, a feature often described as “silent phagocytosis” [108]. This homeostatic population also exhibits selective silencing of TLR responses. When exposed to TLR ligands such as lipopolysaccharides (LPS), these cells show minimal to no proinflammatory cytokine production, such as tumor necrosis factor (TNF)‑α, IL‑1β, or IL‑12, and instead constitutively or inducibly produce anti‑inflammatory mediators, most notably IL‑10. This state of quiescence is stringently maintained by the robust expression of intracellular negative regulators, including SOCS1, IRAK‑M, and A20 [109]. This homeostatic program, while essential for peaceful coexistence with the microbiota, is inherently vulnerable to disruption. Acute epithelial injury and microbial translocation can abruptly reverse this tolerogenic state, while chronic exploitation of these same anti‐inflammatory and tissue‐supportive properties may facilitate tumor immune evasion.

### 2.7 Intestine

The small intestine is the principal site for nutrient digestion and absorption, with its mucosal surface structured into millions of finger‐like villi that vastly expand the functional surface area. Unlike the colon, the small intestinal environment must cope with the episodic influx of dietary antigens and chyme while maintaining coexistence with a relatively sparse but rapidly turning‐over microbiota. This dynamic niche imposes distinct functional demands on small intestinal TRMs, rendering them one of the macrophage populations that best exemplify the coupling of immune surveillance with tissue repair.

The developmental origins of small intestinal macrophages closely mirror those of the colon, sharing the dual‐track system of embryo‐derived long‐lived cells and continuous monocyte replenishment. However, two key distinctions set them apart. First, the maturation of small intestinal macrophages does not fully recapitulate the profound “inflammatory anergy” observed in the colon [108]. The small intestinal environment contains higher concentrations of dietary‐derived fatty acids and bile acids, which fine‐tune macrophage gene expression through nuclear receptors such as PPARγ and VDR, allowing these cells to retain a degree of metabolic activity while preserving an anti‐inflammatory tone. Second, using single‐cell transcriptomics and fate‐mapping approaches, Viola et al. identified Tim‐4 as a marker of long‐lived macrophages in both the LP and muscularis, and uncovered CD163 as a discriminatory marker distinguishing submucosal/muscularis (S/M) from LP macrophages. Mechanistically, TGF‐β signaling was shown to be essential for maintaining LP‐resident macrophages, but dispensable for their S/M counterparts. These findings challenge the paradigm that long‐lived macrophages are restricted to the muscularis and reveal TGF‐β as a layer‐specific cue governing regional macrophage identity and intestinal homeostasis [110]. The most prominent functional specialization of small intestinal macrophages is their role in epithelial renewal. Within the crypt base stem cell niche, they secrete WNT ligands, EGF family ligands, and PGE_2_ to directly support Lgr5^+^ stem cell self‐renewal and barrier restitution [111]. Muscularis macrophages form intimate connections with enteric neurons, secreting BMP2 that activates BMPR on neurons to modulate peristalsis. Enteric neurons, in turn, provide CSF‐1 that is required for macrophage maintenance, establishing a bidirectional neuroimmune communication axis that regulates gastrointestinal motility [112].

The pathological vulnerabilities of small intestinal TRMs mirror those of the colon: barrier disruption during sepsis can reverse homeostatic tolerance into inflammatory amplification, while chronic tumor‐derived signals can subvert their tissue‐supportive properties toward immunosuppression. A distinctive feature of the small intestine is its role as a direct site for peritoneal seeding of abdominal and pelvic malignancies, where serosal TRMs establish premetastatic niches that facilitate tumor implantation.

### 2.8 Peritoneal Macrophage

The peritoneal cavity is a unique serosal compartment whose mesothelial lining and resident immune cells together create an ecosystem for maintaining tissue homeostasis, sensing injury, and orchestrating inflammatory responses. Peritoneal macrophages are the most abundant immune cell population within this space. Suspended in peritoneal fluid or attached to milky spots on the omentum, they constitute the body's first line of defense against infectious threats originating from the gut or other abdominal organs. Peritoneal macrophages reside in an environment that, although relatively quiescent under physiological conditions, must remain perpetually poised to respond to sudden infection or injury.

The composition of peritoneal macrophages is markedly heterogeneous. Under homeostatic conditions in the mouse, two major subsets with distinct morphology, phenotype, and function can be defined: large peritoneal macrophages (LPMs) and small peritoneal macrophages (SPMs) [113]. LPMs are the dominant population in the steady state, are developmentally and functionally confined to the peritoneal cavity, and are formed during embryonic life as long‐lived, TRMs. They express high levels of F4/80, CD11b, and the transcription factor GATA6, while displaying low MHC Class II expression. GATA6 has been identified as the master transcription factor that drives the core gene program of LPMs, and its expression is dependent on retinoic acid signaling within the local peritoneal microenvironment [114]. Apart from their primary phagocytic function, LPMs fulfill an essential homeostatic role by achieving efficient clearance of apoptotic cells, which is crucial for the maintenance of self‐tolerance. In contrast, SPMs are derived from circulating monocytes and express lower levels of F4/80 but higher levels of MHC Class II molecules [115]. Functionally, they are more oriented toward producing proinflammatory cytokines and serving as antigen‐presenting cells (APCs), representing a reserve force poised for mounting rapid inflammatory reactions [116].

A hallmark behavior of peritoneal macrophages is the “milky spot reaction,” whereby LPMs rapidly exit the peritoneal cavity independently of vascular adhesion molecules and migrate through lymphatic stomata to omental milky spots in response to pathogenic or sterile inflammatory stimuli [117]. This response concentrates effector cells to contain infection and prevents microbial dissemination within the peritoneal cavity. During this process, LPMs are massively depleted, setting the stage for subsequent inflammatory reprogramming and the initiation of adaptive immune responses [118].

The pathological significance of peritoneal macrophage plasticity is profound. In abdominal sepsis or acute pancreatitis, peritoneal macrophages are among the first cells to sense pathogenic or danger signals and to initiate a robust inflammatory response. If unchecked, this protective reaction can drive a local hyperinflammatory cascade that contributes to systemic inflammatory response syndrome (SIRS) and multiple organ failure [119, 120]. In the later stages of sepsis, peritoneal macrophages can also sink into profound immunoparalysis [121]. In the tumor microenvironment, such as peritoneal metastases commonly seen in ovarian, pancreatic, and gastric cancers, peritoneal TAMs are deeply educated by tumor cells and the ascitic microenvironment [122]. The GATA6‐driven LPM transcriptional program can be subverted by tumor‐derived signals, leading to the acquisition of tumor‐promoting functions, including suppression of T‐cell and NK‐cell activity, promotion of angiogenesis, and facilitation of metastasis [123, 124]. This functional switch from antimicrobial vanguard to protumor accomplice exemplifies the extremes of macrophage plasticity.

Peritoneal macrophages thus serve as an ideal model for studying the functional paradox of macrophages in acute lethal infections and malignant diseases. Comparative dissection of the shared and divergent reprogramming mechanisms in peritoneal macrophages across these two disease states will provide a valuable foundation for developing macrophage‐targeting strategies capable of addressing both septic immunoparalysis and antitumor immunity.

## 3 TRMs in Infection‐Driven Inflammation: Lessons From Sepsis

While the inflammatory response spans a broad spectrum from acute, self‐limiting host defense to chronic, smoldering tissue damage, macrophages serve as central architects across this entire continuum. Infection represents one of the critical causes of inflammation, among which lethal infection or sepsis has been well discussed. To provide a structured framework, we first summarized the infection sources and routes of pathogen entry. Next, according to the progression of disease, we focus on three contexts that collectively recapitulate the functional spectrum of macrophage plasticity. Physiologically, after encountering the pathogens, macrophages plays a crucial role in the first‐line defense, immune coordination with other types of immune cells, and maintenance of homeostasis; if the infection and inflammation is not well‐controlled and sepsis has become inevitable, macrophages become a key factor triggering the transition from homeostasis to hyperinflammation and tissue damages by initiating immune dysregulation, converging tipping points and further amplifying the intercellular positive feedback of inflammation loops. During the sepsis‐induced immunosuppression, macrophage experience dynamic changes in the metabolic shift, mitochondrial dysfunction and epigenetic imprinting, which participates in the persistent inflammation–immunosuppression paradox.

### 3.1 Etiology: Pathogen Classification and Routes of Invasion

Sepsis‐causing microorganisms are broadly classified into obligate pathogens and opportunistic pathogens. Obligate pathogens, including Staphylococcus aureus, Streptococcus pneumoniae, and Pseudomonas aeruginosa, possess inherent virulence factors that enable infection even in immunocompetent hosts. Opportunistic pathogens, such as Enterococcus faecalis, Klebsiella pneumoniae, and Candida albicans, typically cause disease only when host defenses are compromised, such as in patients receiving immunosuppressive therapy, undergoing prolonged hospitalization, or with indwelling medical devices [125].

The routes of microbial invasion into the bloodstream are equally critical for understanding sepsis pathogenesis. Microorganisms enter the systemic circulation through four major pathways: (i) transmucosal invasion across the epithelial barrier of the respiratory, gastrointestinal, or genitourinary tract; (ii) transmural translocation from the intestinal lumen across an intact or disrupted bowel wall without overt mucosal breach; (iii) direct inoculation via intravenous catheters, surgical wounds, or penetrating trauma; and (iv) handborne transmission in neonatal intensive care units, where healthcare workers’ hands serve as vectors for nosocomial pathogens [126].

Among these, transmural translocation of commensal microbes represents a particularly important but often underappreciated mechanism of sepsis. The intestinal tract harbors the largest reservoir of commensal bacteria in the human body. Under physiological conditions, the intestinal mucosal barrier, comprising the mucus layer, epithelial tight junctions, and gut‐associated lymphoid tissue, effectively prevents microbial translocation [127, 128]. However, biological (e.g., pathogen‐induced disruption of tight junction proteins), chemical (e.g., acid injury, drugs), and physical (e.g., surgical trauma, burns, ischemia–reperfusion) insults can compromise barrier integrity [128]. This disruption allows enteric commensals, including Escherichia coli, Enterococcus species, and Bacteroides fragilis, to translocate into the mesenteric lymph nodes and subsequently the bloodstream, triggering systemic inflammation and sepsis, a phenomenon termed gut‐derived sepsis or bacterial translocation. This mechanism is particularly common in critically ill patients with hemorrhagic shock, major burns, or acute‐on‐chronic liver failure [127].

The clinical significance of these pathways is underscored by epidemiological data. In a systematic review and meta‐analysis of neonatal sepsis, Okike et al. reported that Gram‐negative bacteria accounted for 53% of early‐onset and 71% of late‐onset sepsis cases globally. In low‐ and middle‐income countries, Klebsiella species (31.7%) and S. aureus (17.5%) were the predominant pathogens, contrasting with Streptococcus agalactiae in high‐income countries [129]. Furthermore, gut microbiota dysbiosis, characterized by reduced abundance of beneficial commensals (e.g., Lactobacillus, Bifidobacterium) and overgrowth of potentially pathogenic microorganisms, correlates with increased sepsis susceptibility and poor prognosis [127, 130]. Schlechte et al. emphasized that despite being spatially confined to the intestinal lumen, the gut microbiota profoundly regulates systemic immune cell development and function, with dysbiosis linked to aberrant systemic inflammation and immune‐mediated organ injury in sepsis [131].

### 3.2 The Guardian Response: Early Anti‐Infectious Defense by TRMs

During the anti‐infectious immunity, TRMs serve as the “Guardian” of the immune system, exerting anti‐infectious effects through three aspects: the first is first‐line defense, which involves rapid pathogen recognition, phagocytosis and lysosomal degradation and early cytokine/chemokine production to initiate immune response; the second is immune coordination, including antigen presentation and interacting with other types of immune cells, which activates and bridges innate and adaptive immune responses; the third is homeostasis maintenance, including efferocytosis of apoptotic cells, remodeling ECM, and regulating angiogenesis, which prevents excessive inflammatory damage while clearing the infection.

#### 3.2.1 First‐Line Defense

##### 3.2.1.1 Pathogen Recognition

Macrophages serve as sentinels of innate immunity through a multilayered defense strategy. As macrophages are among the first cells to encounter and respond to invading pathogens, the recognition of pathogens by these cells is a fundamental aspect of their function in the first line of defense during anti‐infectious immunity. Pathogen recognition is mediated by an array of PRRs, including TLRs (TLR4 for LPS detection), C‐type lectins (Dectin‐1 for fungal β‐glucans), and scavenger receptors (MARCO in AMs). Recent single‐cell transcriptomic studies have revealed tissue‐specific PRR specialization: splenic macrophages prioritize TLR9 for systemic DNA pathogen sensing, whereas hepatic Kupffer cells upregulate CD36 to clear bloodborne endotoxin complexes, highlighting their compartmentalized roles in sepsis [132, 133]. In addition, macrophages utilize other signaling pathways to modulate their responses to pathogens. The activation of transcription factors such as cAMP‐response element binding protein in macrophages by pathogens such as *Mycobacterium tuberculosis* can promote bacterial survival by altering macrophage responses. These changes include reducing NF‐κB nuclear transit and limiting phagolysosome fusion, which are critical processes in pathogen clearance. Understanding these pathways provides insights into how pathogens can manipulate macrophage functions to evade immune responses [134].

##### 3.2.1.2 Phagocytosis and Lysosomal Degradation

Once a pathogen is recognized, macrophages initiate phagocytosis. The phagocytic process involves the engulfment of the pathogen into a phagosome, which then fuses with lysosomes for degradation [135]. Phagocytosis is a fundamental process by which macrophages engulf and digest pathogens and thereby orchestrating anti‐infectious immune responses. Phagocytosis and lysosomal degradation involve tightly orchestrated steps: actin‐driven engulfment, phagosome maturation, and acidification. The bicarbonate transporter solute carrier family 4, sodium bicarbonate cotransporter, member 7 (SLC4A7) has been identified as critical for phagosome acidification in macrophages, and its loss reduces the intracellular microbicidal capacity [136]. LC3‐associated phagocytosis (LAP), a noncanonical autophagy pathway, links nutrient sensing to pathogen degradation under metabolic stress, demonstrating the integration of metabolic and immune functions [137, 138].

Besides pathogen clearance, phagocytosis and lysosomal degradation also regulates inflammation and tissue repair. The uptake of apoptotic cells, a process known as efferocytosis, is particularly important in resolving inflammation and promoting tissue healing. Efferocytosis by macrophages leads to the production of anti‐inflammatory cytokines and the suppression of proinflammatory responses, thereby contributing to the resolution of inflammation and the restoration of tissue homeostasis [139, 140]. The E3 ubiquitin ligase RNF13, for example, suppresses TLR lysosomal degradation, thereby promoting inflammatory responses in macrophages [141]. These findings indicate that lysosomal degradation pathways can be targeted to modulate immune responses, suggesting potential therapeutic strategies for inflammatory diseases.

##### 3.2.1.3 Early Cytokine/Chemokine Production

Macrophages play a pivotal role in the early stages of the immune response by producing cytokines and chemokines that orchestrate anti‐infectious immunity. These signaling molecules are crucial for the recruitment and activation of immune cells, thereby enhancing the ability to combat infections. The production of cytokines such as IL‐1, TNF‐α, and IFN‐γ by macrophages can initiate a cascade of immune responses that are vital for controlling infections [142, 143]. While chemokines such as CCL2, CCL3, and CXCL10 are involved in the recruitment of monocytes, neutrophils, and lymphocytes to the site of infection, thereby facilitating a robust immune response [144, 145]. The coordinated expression of these chemokines ensures the timely arrival of immune cells, which are essential for effective pathogen clearance.

The regulation of cytokine and chemokine production by macrophages is a complex process involving various signaling pathways. For example, the activation of the NLRP3 inflammasome in macrophages leads to the production of IL‐1β and IL‐18, which are crucial for the amplification of the inflammatory response [146]. Additionally, the roles of PRRs in detecting PAMPs and initiating cytokine production are well documented [147].

Conversely, the cytokine milieu also regulates the polarization of macrophages, either into a proinflammatory M1 phenotype or an anti‐inflammatory M2 phenotype. M1 macrophages, which are activated by IFN‐γ and LPS, are known for their ability to produce proinflammatory cytokines and enhance pathogen clearance [148, 149]. M2 macrophages, which are induced by ILs such as IL‐4 and IL‐13, are involved in tissue repair and the resolution of inflammation [150].

Collectively, the early production of cytokines and chemokines by macrophages is a fundamental aspect of the innate immune response, orchestrating the recruitment and activation of immune cells necessary for effective anti‐infectious immunity. Understanding the mechanisms underlying macrophage activation and cytokine production can provide insights into the development of therapeutic strategies for infectious diseases.

#### 3.2.2 Immune Coordination

##### 3.2.2.1 Antigen Presentation

Antigen presentation by macrophages is pivotal for orchestrating anti‐infectious immune responses, but with important anatomical and functional limitations. Unlike dendritic cells, which specialize in priming naïve T cells within secondary lymphoid organs, macrophages are anatomically restricted to sites of infection and injury. Within these local microenvironments, macrophages effectively present antigens to effector and central memory T cells rather than to naïve T cells. They achieve this function by processing and presenting antigens via MHC molecules: MHC Class I presents endogenous antigens to CD8^+^ T cells, whereas MHC Class II presents exogenous antigens to CD4^+^ T cells. The regulation of MHC expression involves various signaling molecules and transcription factors, such as NLRC5, which has been identified as a key regulator of MHC Class I‐dependent immune responses [151, 152].

Critically, the interaction between macrophages and T cells at infection sites is bidirectional. Macrophages not only present antigens to effector T cells but also receive help from these T cells in the form of IFN‐γ and other cytokines, which in turn enhances macrophage phagocytic and bactericidal activity. This reciprocal crosstalk represents a critical amplification mechanism for local host defense. Through the secretion of cytokines and chemokines, macrophages also regulate the recruitment and activation of other immune cells, such as dendritic cells and additional T cells, influencing dendritic cell function and maturation [153, 154].

Thus, the antigen‐presenting function of macrophages is not a substitute for dendritic cell‐mediated naïve T cell priming but rather a complementary mechanism that amplifies and focuses the effector T cell response at the site of infection. Understanding this anatomically restricted, bidirectional crosstalk between macrophages and T cells is crucial for developing novel therapeutic strategies to enhance immune responses and improve disease outcomes.

##### 3.2.2.2 Crosstalk With Neutrophils and NK Cells

Macrophages play a pivotal role in orchestrating anti‐infectious responses by regulating the activities of other immunocytes through cell–cell interactions. These versatile cells are strategically located throughout tissues in the body, where they ingest and process foreign materials, dead cells, and debris and recruit additional macrophages in response to inflammatory signals. Their ability to rapidly change function in response to local microenvironmental signals allows them to mediate both protective and pathogenic functions in antimicrobial defense, antitumor immune responses, metabolism, and tissue homeostasis [155]. Recent studies reveal that macrophage metabolic reprogramming serves as a key intrinsic mechanism underlying this functional plasticity. During sepsis, the transition from oxidative phosphorylation to aerobic glycolysis (the Warburg effect) not only fuels inflammasome activation but also epigenetically remodels macrophages through histone lactylation, locking them into either hyperinflammatory or immunosuppressive states depending on disease phase [156].

The interactions between macrophages and other immune cells are crucial for mounting an effective immune response. For example, CD169^+^ macrophages in the spleen are vital for the rapid initiation of antibacterial responses. They control initial bacterial growth and mediate the transport of bacteria to splenic T‐cell zones, which is essential for driving the reorganization of innate immune cells into hierarchical clusters and for local IFN‐γ production near sites of bacterial replication foci [157]. The crosstalk with other immune cells are crucial in the context of viral infections, where macrophages can influence the balance between the effective elimination of the pathogen and the prevention of fatal tissue damage [158]. Mechanistically, this process depends on the CD169‐dependent capture of bacteria‐coupled apoptotic bodies, which are then transferred to CD8α^+^ dendritic cells for cross‐presentation, a pathway regulated by the transcription factor IRF8 [159, 160]. In viral infections, macrophages employ the cGAS–STING pathway to sense cytosolic nucleic acids, leading to Type I IFN production that bridges innate sensing to adaptive T cell activation. This signaling axis is tightly regulated by the deubiquitinase USP13, which prevents STING from undergoing lysosomal degradation, thereby sustaining effective antiviral responses [161].

The plasticity of macrophages allows them to perform a variety of functions in response to changing tissue contexts, such as those imposed by aging or disease. This plasticity is particularly intriguing given their dichotomous role in protecting against or accelerating diseases of the cardiovascular system and the eye [162]. Emerging evidence indicates that epigenetic reprogramming via histone modifications (H3K4me3, H3K27ac, H3K18la) and DNA methylation underlies this plasticity, enabling macrophages to retain memory of prior inflammatory encounters and exhibit either trained immunity or tolerance [163, 164, 165]. Furthermore, macrophages are involved in the regulation of inflammasome pathways, which are crucial for controlling inflammation and tissue repair [166]. Specifically, the NLRP3 inflammasome in macrophages is activated by a two‐signal mechanism: priming via NF‐κB and activation via potassium efflux or mitochondrial damage [167]. This activation leads to gasdermin D‐mediated pyroptosis and the release of IL‐1β and IL‐18, processes that are modulated by metabolic checkpoints such as O‐GlcNAcylation [168] and by interactions with neutrophils via NETosis‐derived HMGB1 [169]. Understanding the multifaceted roles of macrophages in immune regulation and tissue homeostasis is essential for developing novel therapeutic strategies to modulate immune responses and enhance host defense.

#### 3.2.3 Maintenance of Tissue Homeostasis

##### 3.2.3.1 Efferocytosis of Apoptotic Cells

Macrophage efferocytosis, the process by which macrophages engulf and eliminate apoptotic cells, plays a pivotal role in maintaining tissue homeostasis, particularly during anti‐infectious immunity [170]. This process ensures that apoptotic cells are cleared efficiently, preventing the release of potentially harmful intracellular contents that could trigger inflammation or autoimmunity. The importance of efferocytosis in tissue homeostasis is underscored by its role in resolving inflammation and promoting tissue repair after infection or injury [171].

In the context of anti‐infectious immunity, macrophages are essential for both pathogen clearance and the removal of apoptotic cells. This dual role is critical for preventing excessive inflammation and ensuring a balanced immune response. For example, during *Trypanosoma cruzi* infection, macrophages not only phagocytose the parasites but also eliminate apoptotic cells through efferocytosis, which helps regulate the immune response and prevents chronic inflammation [172]. Similarly, in the lungs, efferocytosis by macrophages is crucial for controlling inflammation and maintaining homeostasis, particularly in the context of microbial infections [173].

The efficiency of efferocytosis can be influenced by various factors, including the presence of specific receptors and signaling pathways. Axl receptors have been shown to induce efferocytosis and modulate macrophage responses during *Trypanosoma cruzi* infection, highlighting the complex interplay between efferocytosis and immune regulation [174]. Additionally, the expression of receptors such as MERTK is critical for the effective clearance of apoptotic cells, and the dysregulation of these receptors can lead to impaired efferocytosis and contribute to disease progression [175].

Apart from clearing apoptotic cells, efferocytosis is also crucial for modulating macrophage phenotypes. It drives macrophages toward an anti‐inflammatory phenotype, which is essential for resolving inflammation and promoting tissue repair. The efferocytosis of apoptotic cells can lead to the production of anti‐inflammatory cytokines such as IL‐10, which help maintain immune homeostasis during inflammatory conditions [176].

In summary, macrophage efferocytosis is a critical component of tissue homeostasis during anti‐infectious immunity. It ensures the efficient clearance of apoptotic cells, prevents excessive inflammation, and promotes tissue repair. Understanding the mechanisms underlying efferocytosis and its regulation can provide valuable insights into the development of therapeutic strategies for inflammatory and autoimmune diseases.

##### 3.2.3.2 ECM Remodeling

Macrophages are versatile immune cells that significantly contribute to anti‐infectious immunity, primarily through their ability to remodel the ECM. This remodeling process is essential for maintaining tissue homeostasis and facilitating immune responses. Macrophages can adopt different phenotypes, such as the proinflammatory M1 phenotype and the anti‐inflammatory M2 phenotype, which enable them to perform a variety of functions, including pathogen clearance and tissue repair. The ECM provides structural support and biochemical signals that influence macrophage behavior, thereby impacting their roles in immune responses [177, 178].

The interaction between macrophages and the ECM is particularly important in the context of inflammation and infection. During an infection, macrophages are recruited to the injury/infection site, where they engage in ECM remodeling to facilitate immune cell infiltration and pathogen clearance. This process involves the secretion of matrix metalloproteinases (MMPs) and other enzymes that degrade ECM components, allowing immune cells to access the site of infection more effectively [179, 180].

Moreover, they produce cytokines and growth factors, and thereby modulating the behavior of other cells within the tissue microenvironment. For example, macrophages can promote the activation of fibroblasts, leading to increased ECM production and tissue repair. This dynamic interaction between macrophages and the ECM is crucial for resolving inflammation and restoring tissue integrity after infection [181, 182].

Overall, the ability of macrophages to remodel the ECM is a critical aspect of their function in anti‐infectious immunity and other physiological and pathological processes. Understanding the mechanisms underlying macrophage–ECM interactions can provide insights into the development of novel therapeutic approaches for infectious diseases, cancer, and other conditions characterized by dysregulated ECM remodeling.

##### 3.2.3.3 Angiogenesis Regulation

**FIGURE 2:**
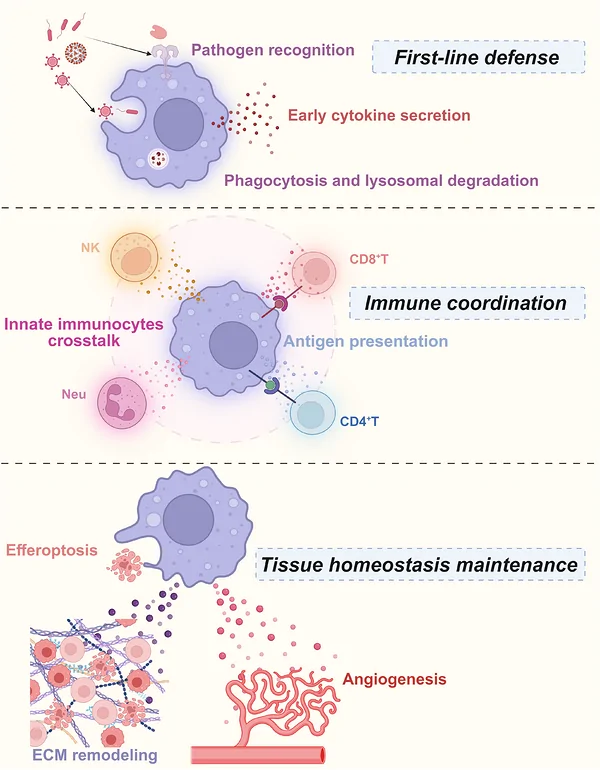
Macrophages in anti‐infectious immunity. The role of macrophages in preventing infection can be divided into three parts. As a part of the first‐line defense (top panel), macrophages play crucial roles in pathogen recognition, pathogen phagocytosis, and lysosomal degradation and promote the early release of cytokines to initiate immune responses. After initiation, macrophages coordinate with other types of immunocytes (middle panel), activate natural killer (NK) cells and neutrophils (Neu), and present pathogen‐derived antigens to CD4^+^T and CD8^+^T cells. In addition, macrophages maintain tissue homeostasis (bottom panel) via the efferocytosis of cells experiencing regulated cell death, remodeling of the extracellular matrix (ECM) and regulation of angiogenesis.

Macrophages play a pivotal role in maintaining tissue homeostasis during infection through their proangiogenic capabilities, which bridge antimicrobial defenses and tissue repair. Recent studies reveal that during infection, macrophage‐produced IL‐1β promotes arteriogenesis by the autocrine STAT3‐ and NF‐κB‐mediated transcription of proangiogenic VEGF‐A [183]. Similarly, FGF is another growth factor secreted by macrophages that plays a crucial role in angiogenesis and tissue regeneration. FGF signaling is involved in various cellular processes, including cell proliferation, differentiation, and survival. FGF can work synergistically with VEGF to enhance angiogenic responses, thereby promoting more robust vascularization under both physiological and pathological conditions [184, 185]. Specifically, TLR4 activation by PAMPs increases MMP‐9 secretion, which not only degrades the ECM to facilitate vascular sprouting but also releases matrix‐bound angiogenic factors [186]. The temporal regulation of these mechanisms involves crucial early‐phase angiogenesis that supports leukocyte trafficking, whereas later‐stage vascular remodeling facilitates metabolic waste clearance and tissue regeneration. Importantly, single‐cell RNA sequencing analyses demonstrated that proangiogenic macrophages transiently coexpressed both M1 (inducible nitric oxide synthase [iNOS^+^]) and M2 (Arg1^+^) markers during helminth infections, suggesting a hybrid phenotype tailored for concurrent pathogen containment and vascular repair [187]. These findings redefine macrophage plasticity as a spatiotemporal coordinator of immune‒vascular crosstalk, where controlled angiogenesis prevents ischemic tissue damage without fostering pathogen dissemination (Figure 2).

### 3.3 The Tipping Point: Transition to Hyperinflammation and Tissue Damage

During sepsis, TRMs initially serve as first responders, containing infection and orchestrating protective inflammation. However, when the inflammatory burden exceeds a critical threshold, whether from high pathogen load, sustained PAMP/damage‐associated molecular patterns (DAMP) exposure, or failure of endogenous brakes, these same macrophages undergo functional conversion. They begin to release excessive proinflammatory mediators and lose their capacity for tissue protection, driving the system past a point of no return. This section examines how this transition unfolds. It first considers the events that destabilize macrophage homeostasis, then identifies the key moments at which protective macrophages convert to drivers of tissue injury and finally describes the feed‐forward loops that lock this hyperinflammatory state in place.

#### 3.3.1 Triggers of Immune Dysregulation

Under normal conditions, macrophage activation is tightly restrained by negative feedback circuits. TLR signaling induces not only proinflammatory cytokines but also anti‐inflammatory mediators such as IL‐10 and SOCS proteins that terminate the response [188]. The tipping point occurs when proinflammatory inputs overwhelm these regulatory circuits, causing the brakes to fail and activation to become self‐amplifying.

The critical factor driving macrophages past this tipping point is the simultaneous engagement of multiple PRRs beyond a critical threshold [189]. When macrophages encounter only a single PAMP, such as LPS alone, NF‐κB activation is balanced by negative feedback. However, when multiple PAMPs are present simultaneously, LPS, flagellin, bacterial DNA, the cumulative signal exceeds the capacity of negative feedback, and proinflammatory gene expression becomes dominant and self‐sustaining.

Pathogen virulence factors can trigger immune dysregulation in individuals with sepsis. Bacterial outer membrane vesicles (OMVs) are a particularly potent trigger because they deliver multiple PAMPs directly into the macrophage cytosol [190]. Unlike free LPS, which engages only surface TLR4, OMVs activate both surface TLRs and cytosolic Caspase‐11 simultaneously. Studies demonstrate that OMVs induce significantly higher IL‐1β and TNF‐α release compared with equivalent amounts of free LPS, generating a synergistic signal that far exceeds the sum of individual inputs [191]. The ability of OMVs to activate the noncanonical inflammasome requires host guanylate binding proteins, further indicating that OMV‐mediated signaling is a multistep, regulated process rather than a simple diffusion event [192].

A second critical input is the loss of negative feedback regulation. Host genetic variants affecting TLR signaling, SOCS proteins, or IRAK‐M expression can shift the activation threshold, making some individuals more vulnerable to tipping at lower levels of stimulation [188]. Sustained TLR signaling can induce epigenetic changes that silence anti‐inflammatory genes while leaving proinflammatory genes poised for rapid expression [188]. Once this epigenetic state is established, the macrophage is locked into a proinflammatory configuration, and activation continues even after the original trigger is removed.

Thus, the tipping point of immune dysregulation is reached when the intensity or duration of PRR signaling overwhelms the capacity of negative feedback systems, when multiple activating pathways are engaged simultaneously, or when epigenetic reprogramming locks macrophages into a proinflammatory state. Once this threshold is crossed, macrophage activation becomes self‐sustaining, transforming these cells from protective effectors into drivers of systemic inflammation. This dysregulated macrophage state initiates the cascade toward SIRS and organ failure, marking the point at which a localized infection transitions into a life‐threatening host response.

#### 3.3.2 Critical Tipping Points

The transition from controlled host defense to dysregulated hyperinflammation is not driven by any single event but by the convergence of three interconnected tipping points within macrophages. Individually, each represents a failure of normal regulatory control; together, they create a self‐sustaining circuit that locks macrophages into a pathogenic state.

The first tipping point is NLRP3 inflammasome hyperactivation. In sepsis, macrophages encounter both PAMPs (from pathogens) and DAMPs (from injured tissues). This dual signal triggers assembly of the NLRP3 inflammasome, activating Caspase‐1 and releasing IL‐1β and IL‐18 [193]. Under normal conditions, this response is self‐limiting. However, when PAMP/DAMP stimulation is sustained, NLRP3 becomes hyperactivated, driving a cytokine burst that amplifies systemic inflammation and organ injury [194].

The second tipping point is the loss of negative feedback. Macrophages normally restrain their own activation through SOCS proteins, which inhibit JAK/STAT signaling. SOCS‐3 is rapidly upregulated in macrophages and neutrophils during sepsis in a time‐dependent manner, serving as a critical feedback inhibitor [195]. This dichotomous control demonstrates that loss of SOCS‐mediated feedback fundamentally alters macrophage inflammatory responses.

The third tipping point is the metabolic switch from oxidative phosphorylation to glycolysis. Resting macrophages rely on OXPHOS, a configuration that supports homeostatic functions. Activation triggers a shift to aerobic glycolysis, the Warburg effect, which is required for proinflammatory cytokine production and inflammasome assembly [196]. Critically, once committed to glycolysis, macrophages are locked into this metabolic state, unable to switch back to OXPHOS for resolution. Lcn2 deficiency has been shown to attenuate NLRP3 inflammasome activation by impairing glycolytic activity and reducing mitochondrial ROS (mtROS) production, directly linking metabolic reprogramming to inflammasome activation [194]. Pnpt1, a mitochondrial RNA processing protein, mediates NLRP3 inflammasome activation by controlling both glycolysis and mitochondrial antiviral signaling protein expression, further confirming that metabolic reprogramming is upstream of inflammasome hyperactivation [197]. This metabolic inflexibility makes the inflammatory response self‐perpetuating.

What drives macrophages past the point of no return is not any single tipping point but their convergence and reinforcement. NLRP3 hyperactivation is potentiated by glycolytic metabolism, which provides the metabolic intermediates and rapid ATP required for inflammasome assembly and cytokine production. SOCS deficiency sustains JAK/STAT signaling, which in turn drives expression of glycolytic genes, reinforcing the metabolic switch. The cytokines released through NLRP3 activation further suppress SOCS expression and promote glycolytic reprogramming. These three tipping points thus form an integrated, self‐reinforcing network: once engaged, each pushes the others forward, and together they drive the transition from controlled immunity to irreversible hyperinflammation.

#### 3.3.3 Amplification Loops

Once the tipping point is crossed, the inflammatory response is further amplified by intercellular positive feedback loops involving macrophages, neutrophils, and the complement system. These loops convert localized macrophage activation into a systemic, self‐sustaining inflammatory cascade.

NETs released by activated neutrophils interact directly with macrophages, inducing pyroptosis through HMGB1‐RAGE signaling [197]. This NET‐induced macrophage death releases additional DAMPs, which in turn activate more neutrophils to form more NETs, establishing a self‐amplifying loop [198]. The complement cascade similarly engages in bidirectional amplification with macrophages: C5a generated during sepsis activates macrophages via C5aR1, and activated macrophages secrete additional complement components, generating more C5a and sustaining the loop independently of the original infectious trigger [199]. Macrophages also engage in feed‐forward amplification with endothelial cells. Activated macrophages release cytokines and proteases that damage the vascular endothelium; injured endothelial cells release DAMPs and express adhesion molecules that recruit more monocytes, which differentiate into additional inflammatory macrophages [200].

**FIGURE 3:**
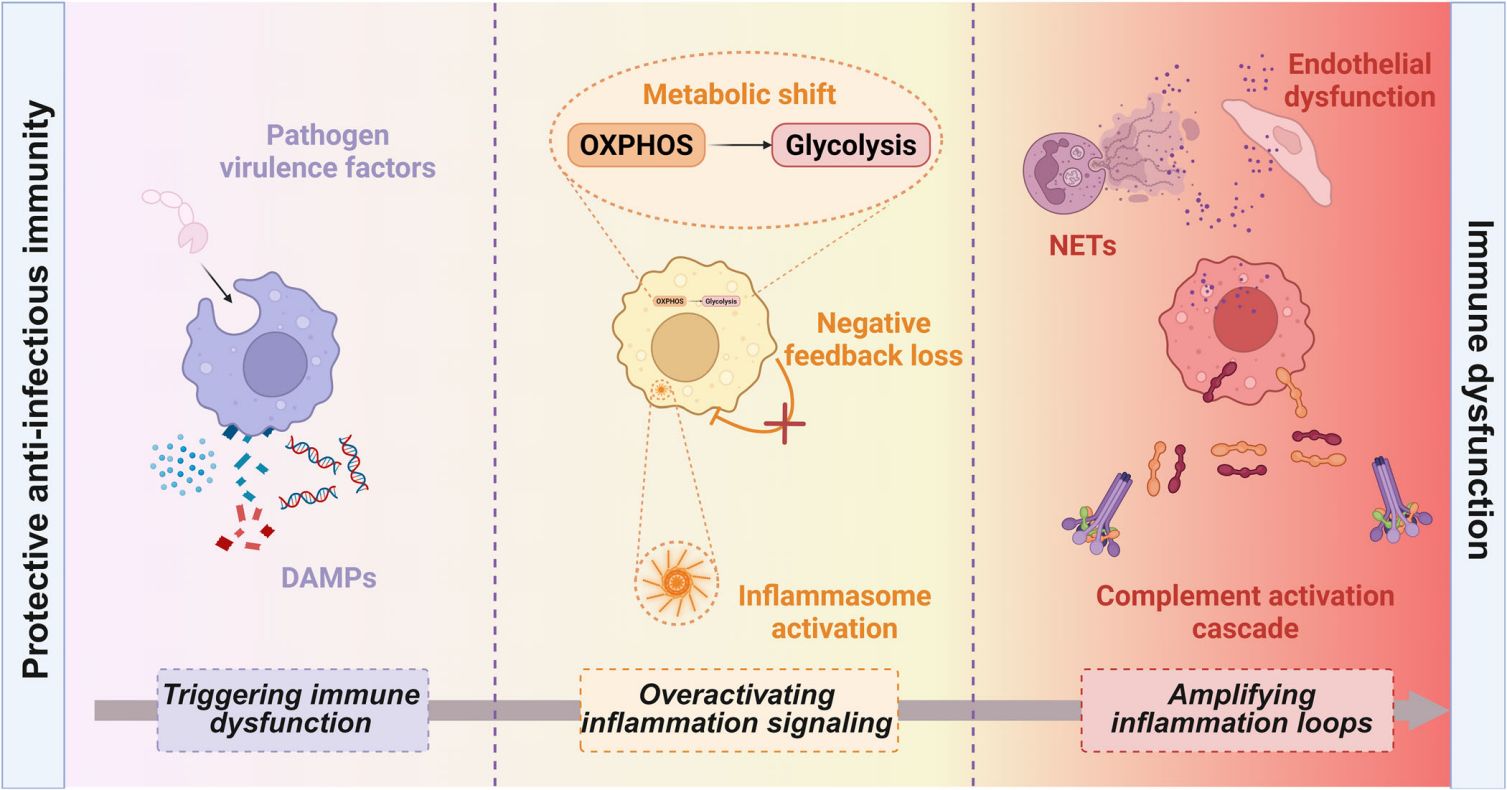
Roles of macrophages in the transition from protective anti‐infectious immunity to immune dysfunction. The transition arises from the release of damage‐associated molecular patterns (DAMPs) after interacting with pathogen virulence factors (left panel). Afterward, the overactivation of inflammatory signaling occurs at a critical tipping point (middle panel), during which macrophages undergo a metabolic shift from oxidative phosphorylation (OXPHOS) to glycolysis, losing negative feedback and leading to inflammasome activation. Finally, the amplification of inflammation loops occurs in a macrophage‐dependent manner (right panel), during which macrophages release cytokines, leading to the formation of neutrophil extracellular traps (NETs) and endothelial dysfunction and triggering the complement activation cascade, resulting in uncontrolled immune dysfunction.

These loops do not operate in isolation. NETs activate complement; complement promotes NET formation; endothelial injury releases DAMPs that trigger both NETosis and complement activation. Macrophages sit at the center of all three loops, serving as both sensors and amplifiers. Once engaged, these loops render the inflammatory response autonomous, the defining feature of the transition from controlled host defense to life‐threatening hyperinflammation (Figure 3).

### 3.4 The Collapse Phase: Immunoparalysis and the Persistent Inflammation–Immunosuppression Paradox

In the late phase of sepsis, macrophages do not simply exhaust themselves after the hyperinflammatory burst. Instead, they enter a paradoxical state in which persistent inflammation coexists with profound immunosuppression. This section examines this macrophage‐centered paradox through its hallmarks as immunosuppression, its clinical manifestation as persistent inflammation–immunosuppression paradox and the intracellular mechanisms, including metabolic shift, mitochondrial dysfunction, and epigenetic imprinting, which lock macrophages into this dysfunctional state.

#### 3.4.1 Immunoparalysis Phase

The immunosuppressive phase of macrophage overactivation is a critical factor contributing to the immunological imbalance observed in sepsis. During sepsis, the immune system undergoes a complex and dynamic response characterized by an initial hyperinflammatory phase followed by a prolonged immunosuppressive phase. The latter is characterized by the dysfunction of immune cells, including macrophages, which play pivotal roles in maintaining immune homeostasis and responding to infections. The upregulation of checkpoint inhibitors, such as PD‐L1 and CD47, represents an aspect of macrophage dysfunction. PD‐L1 can inhibit T‐cell activation, and its overexpression in macrophages can lead to an immunosuppressive environment [201]. CD47, a “don't eat me” signal, is also upregulated in some cases, allowing cancer cells to evade macrophage‐mediated phagocytosis.

These alterations contribute to the immunological imbalance observed in septic patients, highlighting the need for targeted therapies that can restore immune homeostasis and improve patient outcomes.

#### 3.4.2 The Persistent Inflammation–Immunosuppression Paradox

**FIGURE 4:**
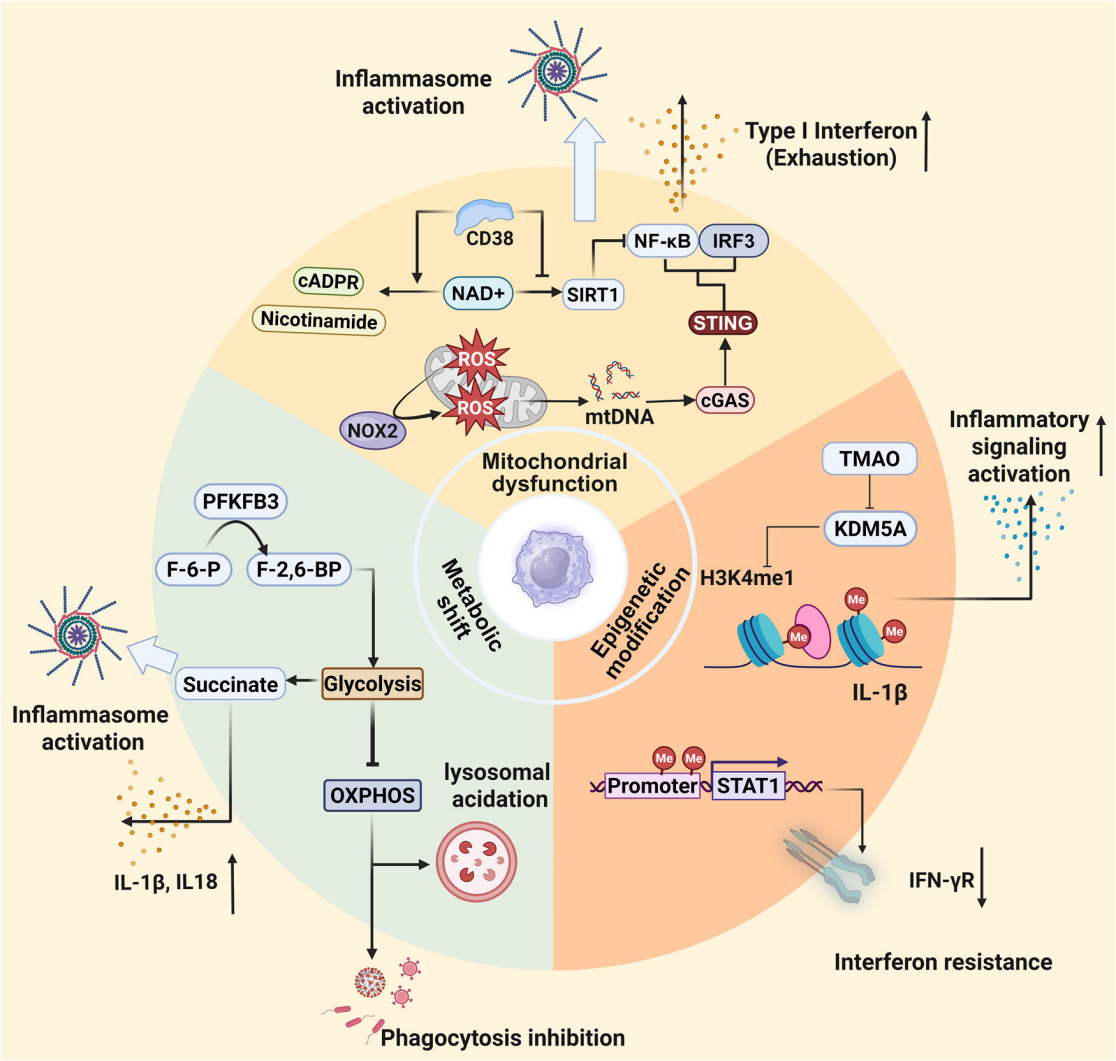
Roles of macrophages in the persistent inflammation–immunosuppression paradox. During the persistent inflammation‒immunosuppression paradox, macrophages experience mitochondrial dysfunction/meltdown (upper panel), metabolic shift/lockdown (lower left panel), and epigenetic modification/imprinting (lower right panel). In the context of mitochondrial dysfunction, the accumulation of “zombie mitochondria” leads to the leakage of mitochondrial ROS (mtROS) and mitochondrial DNA (mtDNA) into the cytosol. mtROS activate NOX2, which further destabilizes mitochondrial membranes via a self‐amplifying loop. mtDNA activates the cGAS–STING pathway, triggering Type I interferon production and leading to T‐cell exhaustion. Moreover, sepsis‐driven CD38 overexpression depletes NAD^+^ into cyclic ADP‐ribose (cADPR) and nicotinamide, activating the NF‐κB signaling pathway and impairing mitochondrial biogenesis. During metabolic shifts, PFKFB3 promotes the transition of fructose‐6‐phosphate (F‐6‐P) into fructose‐2,6‐bisphosphate (F‐2,6‐BP), locking macrophages in Warburg‐like glycolysis, which facilitates the activation of the inflammasome, inhibits oxidative phosphorylation (OXPHOS), and then suppresses ATP‐dependent antimicrobial lysosomal acidification and phagosomal maturation. In terms of epigenetic modifications, the H3K4me1 modification has been shown to play a crucial role. The H3K4me1 modification of the IL‐1β locus primes macrophages for a hyperresponse to secondary challenges during trained immunity, whereas trimethylamine N‐oxide (TMAO) stabilizes H3K4me1 by inhibiting the eraser enzyme KDM5A. In addition, hypermethylation silences the STAT1 promoter, which blunts IFN‐γ signaling and cripples antiviral defenses.

During critical illnesses, the transition from a hyperinflammatory phenotype to a hypoinflammatory phenotype is not only associated with the recovery of organ function but also foreshadows the overall rehabilitation trajectory [202]. However, a distinct paradox can be observed in sepsis, representing a critical juncture. Clinically, this paradox manifests as a biomarker discordance: coexisting hyperinflammation (e.g., IL‐6 >1000 pg/mL) and profound immunosuppression (e.g., CD4^+^ T‐cell lymphopenia <200/µL) [203, 204], a dual‐phase trajectory evolutionarily rooted in the conflicting needs for pathogen eradication and energy conservation; and elevated CRP levels (>150 mg/L) alongside monocytic HLA‐DR suppression (<5000 molecules/cell) [205], a signature of immune exhaustion that defies conventional therapeutic paradigms. We refer to this contradiction in the immune response as the “persistent inflammation‒immunosuppression paradox,” in which macrophage biology intersects with systemic immune collapse (Figure 4).

#### 3.4.3 Metabolic Lockdown

The septic microenvironment imposes a rigid, inflexible metabolic state on macrophages, stripping them of their normal ability to dynamically switch between glycolysis and oxidative phosphorylation. This “metabolic straitjacket” traps them in a dysfunctional loop that perpetuates both hyperinflammation and immune paralysis. At the heart of this dysfunction lies 6‐phosphofructo‐2‐kinase/fructose‐2,6‐biphosphatase 3 (PFKFB3), a glycolytic gatekeeper enzyme that drives excessive fructose‐2,6‐bisphosphate production, locking macrophages into a Warburg‐like metabolism [206, 207, 208]. While this glycolytic surge fuels NLRP3 inflammasome activation through succinate accumulation, it starves OXPHOS, crippling ATP‐dependent antimicrobial functions such as lysosomal acidification and phagosomal maturation [209]. The consequences are dire: macrophages become “metabolically blind” to bacterial threats and are unable to mobilize the OXPHOS‐driven respiratory burst required for intracellular pathogen killing, even as they hypersecrete IL‐1β and IL‐18.

#### 3.4.4 Mitochondrial Meltdown

Simultaneously, mitochondria, metabolic powerhouses, undergo catastrophic meltdown. Oxidized cardiolipin, a phospholipid critical for mitochondrial membrane integrity, forms covalent adducts with cytochrome *c*, rendering mitochondria resistant to mitophagy [210]. These damaged mitochondria escape mitophagy due to oxidized cardiolipin modifications, leading to their pathological accumulation and were referred to as “zombie mitochondria.” These “zombie mitochondria” accumulate, leaking mtROS and mitochondrial DNA (mtDNA) into the cytosol. mtROS fuel a self‐amplifying loop by activating NOX2, further destabilizing mitochondrial membranes [211], while mtDNA fragments activate the cGAS–STING pathway, triggering Type I IFN production that paradoxically exhausts T‐cell responses [212]. Compounding this crisis is NAD^+^ depletion, a metabolic black hole caused by sepsis‐driven CD38 overexpression [213]. CD38, an ectoenzyme, hydrolyzes NAD^+^ into cyclic ADP‐ribose and nicotinamide, depleting the substrate for SIRT1, a deacetylase critical for suppressing NF‐κB signaling and promoting mitochondrial biogenesis [214]. When SIRT1 is silenced, macrophages lose the ability to transition to reparative states, whereas the accumulated nicotinamide inhibits PARP1‐mediated DNA repair, creating a vicious cycle of genomic instability and inflammasome activation. Emerging therapeutic strategies aim to break this cycle: nicotinamide riboside supplementation restores NAD^+^ levels in Phase II trials (NCT03432871), rescuing SIRT1 activity and the OXPHOS capacity, whereas PFKFB3 inhibitors such as AZ67 show promise in preclinical models for uncoupling glycolysis from inflammation [215].

This metabolic‐mitochondrial axis epitomizes the sepsis paradox: macrophages are simultaneously “overfed” with glycolytic metabolites that drive inflammation yet “starved” of the energy and redox balance required for immune coordination, a duality that demands precision interventions to rewire cellular metabolism without extinguishing antimicrobial defenses.

#### 3.4.5 Epigenetic Imprinting

The septic milieu engraves lasting epigenetic scars on macrophages, reprogramming their identity into a maladaptive state that perpetuates immune dissonance. At the forefront is trained immunity, a paradoxical adaptation where transient pathogen exposure leaves enduring epigenetic marks [216, 217]. H3K4me1 modifications at the IL‐1β locus, which are deposited by the histone methyltransferase MLL1, prime macrophages for hyperresponsive secondary challenges, even as the primary infections resolve [218]. This “inflammatory memory” is further amplified by sepsis‐induced microbiota depletion, which increases the circulating levels of TMAO, a gut metabolite that stabilizes H3K4me1 by inhibiting the eraser enzyme KDM5A [219]. Simultaneously, DNMT3a‐mediated hypermethylation silences the STAT1 promoter, blunting IFN‐γ signaling and crippling antiviral defenses [220]. The result is a macrophage population that is paradoxically primed for cytokine overproduction yet unable to respond to adaptive immune cues.

## 4 TRMs in Other Inflammatory Contexts

### 4.1 TRMs in Sterile Inflammation

Sterile inflammation is initiated by DAMPs, endogenous molecules released from necrotic or stressed cells in the absence of viable pathogens [221]. Unlike the PAMP‐driven activation in infectious inflammation, where pathogen‐derived ligands are uniformly present and rapidly detected, sterile inflammation presents macrophages with a fundamentally different challenge: distinguishing between healthy self, injured self, and altered self [222]. This section focuses on the unique mechanisms by which macrophages achieve this discrimination and the consequences when this system fails.

#### 4.1.1 The DAMP Gradient Problem

Unlike the bolus delivery of PAMPs from proliferating pathogens, DAMPs are released in a spatiotemporally heterogeneous manner. At the core of a sterile injury, necrotic cells release high concentrations of intracellular contents, creating a DAMP‐rich zone where macrophages undergo full activation. Toward the periphery, sublethally stressed cells release lower DAMP levels, creating a gradient where macrophages may be primed but not fully activated [223]. This gradient imposes a quantitative challenge on macrophages: they must discriminate not only the presence of DAMPs but also their concentration to calibrate response intensity [224]. Macrophages achieve this through signal integration at the level of the NLRP3 inflammasome, where both priming (signal 1) and activation (signal 2) inputs are required [225]. The threshold for activation is set by the balance between NF‐κB‐dependent priming and the intensity of the second signal [226]. This two‐signal requirement prevents macrophages from responding to low‐level, physiological DAMP release that occurs during normal tissue turnover, thereby maintaining homeostasis.

#### 4.1.2 The NLRP3 Inflammasome as a Metabolic Sensor

A key distinction between sterile and infectious inflammation lies in the central role of the NLRP3 inflammasome as a metabolic sensor. While infectious inflammation activates multiple inflammasome types (NLRC4 for flagellin, AIM2 for DNA), sterile inflammation converges almost exclusively on NLRP3 because this platform responds to cellular stress states rather than specific molecular patterns [227, 228]. The diversity of sterile triggers, including MSU crystals [229], ATP [230], cholesterol crystals [231], amyloid‐β [232], and mtROS [233], converges on a common set of upstream signals: potassium efflux, lysosomal damage, and mitochondrial dysfunction [225]. These are not ligand–receptor interactions in the classical sense but rather homeostatic disruptions that macrophages interpret as evidence of cellular injury.

Mechanistically, MSU and cholesterol crystals are ingested by macrophages into phagosomes. The crystalline structure damages the phagosomal membrane, releasing cathepsin B into the cytosol, which then activates NLRP3 [234]. ATP released from damaged cells engages the P2×7 receptor, which initiates a signaling cascade that leads to the translocation of the two‐pore potassium channel TWIK2 from endosomes to the plasma membrane via Rab11a‐dependent trafficking; TWIK2 then mediates rapid potassium efflux, a potent activator of NLRP3 [235]. Mitochondrial damage releases cardiolipin and mtDNA, both of which directly engage NLRP3 [236]. Notably, the same mitochondrial damage in infectious inflammation would activate the cGAS–STING pathway, creating a differential output [237]. This convergence of diverse sterile triggers on a common effector explains why NLRP3 inhibitors have broad therapeutic potential across crystal arthropathies, ischemia–reperfusion injury, and neuroinflammation [238].

#### 4.1.3 The Self‐Amplifying Loop and Chronicity

A defining feature of sterile inflammation is the potential for self‐amplification without ongoing exogenous stimulation. Pyroptosis, the gasdermin D‐mediated lytic cell death downstream of NLRP3 activation, releases intracellular DAMPs that were previously compartmentalized, including HMGB1, ATP, and histones [239]. These secondary DAMPs activate neighboring macrophages, propagating the inflammatory wave outward from the injury core [240]. This creates a feed‐forward loop that can sustain inflammation even after the initial insult has resolved [241]. In self‐limiting sterile inflammation, efferocytosis terminates this loop by clearing pyroptotic corpses and triggering anti‐inflammatory reprogramming [242]. However, when DAMP clearance is inefficient or when the inciting stimulus persists, the loop becomes self‐sustaining [243].

Gout exemplifies this chronicity. MSU crystals are not readily cleared from joint space [244]. Each gout flare involves cycles of neutrophil infiltration, crystal ingestion, NLRP3 activation, and pyroptosis [245]. Between flares, macrophages remain in a primed state, expressing elevated NLRP3 and pro‐IL‐1β, such that a minor trigger can precipitate another acute attack [246]. This primed but quiescent state is distinct from the immunoparalysis seen in late sepsis, where macrophages become refractory to stimulation. In sterile inflammation, the defect is not in responsiveness but in resolution. Therapeutically, this distinction matters: restoring efferocytosis or promoting proresolving mediators may be more effective than simply blocking IL‐1β in chronic sterile conditions [247].

#### 4.1.4 Tissue‐Specific Variations

The DAMP repertoire and macrophage response vary substantially across tissues. In the ischemic brain, microglia are exquisitely sensitive to ATP released from neurons, responding within minutes through P2Y12 receptor‐mediated process extension and P2×7‐mediated inflammasome activation [248]. In the ischemic heart, resident CCR2^−^ macrophages are relatively resistant to DAMP‐induced pyroptosis, whereas recruited CCR2^+^ monocytes are highly susceptible, creating a time‐dependent shift in inflammatory phenotype [249]. In the joint, synovial macrophages are chronically exposed to mechanical stress and low‐grade DAMP release, maintaining a baseline state of priming that explains the rapid onset of gout flares upon crystal precipitation [250]. In the atherosclerotic plaque, cholesterol crystals accumulate over years, driving continuous low‐grade NLRP3 activation that contributes to plaque progression without triggering overt inflammation until plaque rupture [251]. These tissue‐specific variations suggest that therapeutic strategies targeting sterile inflammation may need to be tailored to both the DAMP profile and the resident macrophage phenotype of the affected organ.

### 4.2 Macrophage in Autoimmune Inflammation: The Type 1 Immune Response

Autoimmune inflammation arises from breakdown of self‐tolerance, driven by self‐antigens rather than pathogens. Unlike acute sterile inflammation, it is chronic and progressive, sustained by persistent autoantigen presentation and self‐amplifying lymphocyte activation. Macrophages serve as APCs, effector cells, and regulators that can either amplify or restrain autoimmune responses. This section focuses on the unique features that distinguish macrophage function in autoimmunity: autoantigen recognition, Fcγ receptor‐mediated activation by immune complexes, antigen presentation, and tissue‐specific effector programs.

#### 4.2.1 The Problem of Self‐Antigen Recognition

Autoimmune inflammation differs fundamentally from infection or sterile injury in how macrophages recognize targets. The antigens are constitutively present self‐molecules that are neither foreign nor released by acute damage. These include nuclear antigens in SLE [252], citrullinated proteins in RA [253], myelin basic protein in MS [254], and GAD65 in Type 1 diabetes [255]. Since macrophages cannot intrinsically distinguish self from nonself, their activation depends on two contextual cues. The first is autoantibody opsonization. Immune complexes engage macrophage Fcγ receptors, making macrophage activation secondary to B and T cell activation [256]. This autoantibody dependence also creates a feed forward loop in which macrophage‐mediated tissue damage releases more self‐antigens, driving further autoantibody production [257]. The second cue is the inflammatory milieu. In the RA joint, TNF‐α, IL‐1, and IL‐6 from activated macrophages and T cells prime‐resident macrophages, upregulating MHC Class II and costimulatory molecules so that self‐antigens normally ignored become immunogenic [258]. The distinction between autoimmunity and sterile inflammation therefore hinges on adaptive immune involvement. Sterile inflammation is innate driven and self‐limiting, whereas autoimmunity involves sustained adaptive responses that render self‐antigens persistently inflammatory [259].

#### 4.2.2 Fcγ Receptor‐Mediated Activation by Immune Complexes

A defining feature of macrophage activation in many autoimmune diseases is the engagement of Fcγ receptors by immune complexes. Unlike the TLR‐mediated activation by PAMPs or the NLRP3 activation by crystals, Fcγ receptor signaling represents a distinct activation pathway with unique downstream consequences. Immune complexes are formed when autoantibodies bind to self‐antigens. These complexes are taken up by macrophages through Fcγ receptors, which recognize the constant region of IgG antibodies [260].

Fcγ receptor cross linking triggers a signaling cascade through immunoreceptor tyrosine‐based activation motif domains in the associated FcRγ chain. This cascade leads to Syk kinase activation, calcium flux, and downstream activation of NF‐κB, MAP kinases, and PI3K pathways [261]. The transcriptional output includes proinflammatory cytokines such as TNF‐α, IL‐1β, and IL‐6; chemokines including CXCL8 and CCL2; and matrix degrading enzymes such as MMPs [262]. Notably, Fcγ receptor activation does not require the two‐signal priming activation sequence that characterizes NLRP3 inflammasome activation. Immune complex‐mediated activation can directly elicit macrophage effector functions, making it a rapid and potent pathway once autoantibodies are present [263].

The pathological consequences of Fcγ receptor activation vary by disease. In SLE, macrophages in the kidney take up immune complexes deposited in the glomerular basement membrane, leading to production of IL‐1β, TNF‐α, and reactive oxygen species that damage podocytes and promote crescent formation [264]. In RA, synovial macrophages internalize immune complexes containing citrullinated proteins, driving production of IL‐1 and TNF‐α that sustain synovitis and activate fibroblasts and osteoclasts [265]. These cytokines perpetuate chronic joint inflammation, with TNF‐α and IL‐1β acting as master regulators of synovial fibroblast activation and osteoclast‐mediated bone erosion, while metabolic exhaustion of synovial macrophages further amplifies tissue damage through PANoptotic cell death [266]. In autoimmune thrombocytopenia, splenic macrophages bearing Fcγ receptors phagocytose antibody opsonized platelets, leading to thrombocytopenia [267, 268]. The therapeutic success of intravenous immunoglobulin in multiple autoimmune diseases is attributed in part to Fcγ receptor blockade, underscoring the centrality of this pathway [269].

#### 4.2.3 Macrophages as APCs in Autoimmunity

Macrophages are now recognized as the dominant APCs for reactivating self‐reactive T cells within autoimmune target tissues, challenging the traditional view that dendritic cells serve this function.

The functional capacity of macrophages as APCs critically depends on costimulatory molecule expression, particularly CD80 and CD86. A study demonstrated that upon acute Treg depletion, macrophages exhibit more prominent CD80/CD86 upregulation than dendritic cells, and complete blockade of CD80/CD86–CD28 interactions reverses all immune activation, preventing lethal autoimmunity [270]. This positions macrophage CD80/CD86 expression as a nonredundant checkpoint in autoimmune T cell activation and provides the mechanistic basis for CTLA4‐Ig therapy already in clinical use.

A feed‐forward mechanism sustains this antigen‐presenting function. Lytic cell death releases autoantigens such as TRIM21, which form immune complexes with circulating antibodies; these complexes are taken up by macrophages, driving further antigen cross‐presentation and metabolic reprogramming in high‐IFN environments [271]. Together, these findings establish macrophages as central APCs that not only initiate but also perpetuate autoreactive T cell responses within target tissues.

#### 4.2.4 Tissue‐Specific Effector Programs

Macrophages in different autoimmune diseases execute tissue‐specific effector programs that reflect the anatomy and function of the target organ. In RA, synovial macrophages differentiate into osteoclasts upon exposure to RANKL (receptor activator of NF‐κB ligand) produced by activated T cells and synovial fibroblasts [272]. Osteoclasts are multinucleated cells specialized for bone resorption, and their formation represents a macrophage lineage switch not observed in other inflammatory contexts [273]. These osteoclasts erode periarticular bone, leading to the characteristic joint erosions of RA. The bone resorption activity depends on acidification of the resorption lacunae and secretion of cathepsin K, a collagenase that degrades bone matrix [274]. This osteoclastogenic program is unique to macrophages in the joint and is not observed in macrophages from other autoimmune tissues.

In systemic lupus erythematosus, macrophages in the kidney and skin specialize in immune complex clearance and Type I IFN production. In the glomerulus, mesangial macrophages internalize immune complexes but often fail to clear them efficiently, leading to complement activation and recruitment of inflammatory cells [275, 276]. Plasmacytoid dendritic cells are classically regarded as the primary Type I IFN producers in SLE, yet recent evidence challenges this paradigm. In cutaneous lupus, only a minority of pDCs stain positive for IFNα, while CD14^+^CD16^+^ macrophages and classical dendritic cells represent the largest contributors of type I IFNs at the tissue level [277]. Regardless of the cellular source, macrophages are prominent responders to the Type I IFN milieu. SLE patients exhibit an IFN‐inducible gene signature in circulating monocytes that persists during monocyte‐to‐macrophage differentiation, and this signature correlates with disease activity [278]. Type I IFN signaling induces macrophage expression of IFN‐stimulated genes including MX1, IFIT1, and IFIT3 [278]. These IFN‐inducible transcripts are also abundantly expressed in tissue macrophages from SLE lesions and serve as biomarkers of disease activity. This IFN signature is a hallmark of SLE and correlates with disease flares.

In multiple sclerosis, microglia and infiltrating macrophages participate in myelin phagocytosis and axonal injury [279]. Myelin debris is phagocytosed through TREM2 and other scavenger receptors [280]. While myelin clearance is necessary for repair, the phagocytic process triggers production of reactive oxygen species and MMPs that damage nearby axons [281, 282]. Chronic microglial activation in MS plaques contributes to the progressive neurodegeneration that underlies irreversible disability. Unlike RA, where macrophage‐derived osteoclasts cause irreversible bone loss, the myelin phagocytosis in MS is potentially reversible if remyelination occurs, but this repair process often fails in chronic disease [283].

In inflammatory bowel disease, intestinal macrophages in the LP adopt a proinflammatory phenotype distinct from their normally tolerogenic state [284]. These activated macrophages produce IL‐23, which drives Th17 cell differentiation and IL‐17 production, leading to neutrophil recruitment and tissue damage [285]. The IL‐23/IL‐17 axis is a therapeutic target in Crohn's disease, and the efficacy of anti‐IL‐23 antibodies underscores the central role of this macrophage–T cell interaction [286].

### 4.3 TRM in Type 2 Inflammation: The Alternative Activation Paradigm

Type 2 inflammation represents a distinct immunological context triggered by helminth infections and allergic stimuli. Unlike the proinflammatory macrophage activation seen in infectious or sterile inflammation, Type 2 inflammation directs macrophages toward an alternative activation state (M2) governed by IL‐4 and IL‐13 signaling. This section focuses on the unique mechanisms that distinguish M2 from M1 polarization, including the signaling pathway, the metabolic commitment to Arginase‐1 (Arg‐1), and the effector functions that define macrophage roles in parasite containment and tissue remodeling.

#### 4.3.1 The IL‐4/IL‐13 STAT6 Axis and the Arginine Fork

The master pathway driving M2 macrophage polarization is the IL‐4/IL‐13 STAT6 axis, which fundamentally differs from the TLR/NF‐κB pathway that initiates M1 activation [287]. IL‐4 and IL‐13 signal through a shared receptor complex, activating JAK1 and JAK3 (or TYK2), which phosphorylate the cytoplasmic tail of IL‐4Rα to create docking sites for STAT6 [288]. Phosphorylated STAT6 dimerizes, translocates to the nucleus, and directly activates transcription of M2‐associated genes including Arg1, Mrc1 (encoding CD206), Retnla (encoding RELMα/FIZZ1), and Chil3 (encoding Ym1) [289]. STAT6 deficiency in mice abrogates M2 macrophage development, and these animals fail to expel helminths, succumbing to disseminated infection [290]. Unlike the two‐signal requirement for NLRP3 inflammasome activation, STAT6‐driven M2 polarization proceeds directly from cytokine engagement, enabling rapid and persistent commitment once the Type 2 cytokine environment is established [291].

The functional specialization of M2 macrophages in Type 2 inflammation is metabolically underpinned by a defining switch in arginine metabolism, creating what can be termed the arginine fork. M1 macrophages express iNOS, which converts l‐arginine to nitric oxide (NO), a potent antimicrobial agent. M2 macrophages instead express Arg‐1, which hydrolyzes l‐arginine to l‐ornithine and urea. Both enzymes compete for the same substrate, and their relative expression determines the functional output. The consequences of Arg‐1 expression are threefold. First, by depleting l‐arginine, Arg‐1 starves helminths of this essential amino acid, impairing larval development [292]. Second, l‐ornithine serves as a precursor for polyamines that promote cell proliferation and for proline that is incorporated into collagen, supporting tissue repair and fibrosis [293, 294]. Third, Arg‐1 activity suppresses iNOS by substrate depletion, effectively turning off the M1 antimicrobial program [295]. This mutual antagonism ensures that macrophages commit to either M1 or M2 polarization rather than adopting a mixed state, with the local concentration of IL‐4/IL‐13 versus IFN‐γ determining which enzyme predominates [296].

#### 4.3.2 Effector Mechanisms and the Primed State in Allergy

M2 macrophages deploy effector mechanisms qualitatively distinct from those of M1 macrophages. Chitinases and chitinase‐like proteins, including Ym1 and Ym2 in mice, bind chitin present in helminth eggshells, although their precise antihelminthic mechanism remains incompletely defined [297]. Resistin‐like molecules such as RELMα/FIZZ1 impair helminth chemotaxis and may interfere with parasite nutrient uptake [298]. Matrix remodeling represents a core M2 function with no parallel in M1 activation. M2 macrophages secrete TGF‐β and PDGF, which activate fibroblasts to proliferate and deposit ECM components, while Arg‐1‐derived proline provides building blocks for collagen synthesis [299]. While this program supports normal wound healing, its persistence in chronic Type 2 inflammation drives pathological fibrosis, as seen in severe asthma where M2 macrophages promote subepithelial fibrosis and airway wall thickening [300].

A distinctive feature of macrophage biology in allergic Type 2 inflammation is the primed state induced by chronic allergen exposure. In asthmatic airways, AMs repeatedly encounter allergens, resulting in sustained STAT6 activation and persistent M2 marker expression [301, 302]. These primed M2 macrophages exhibit exaggerated responses to subsequent allergen challenge, producing more IL‐10 and TGF‐β than naive macrophages. This primed state involves epigenetic remodeling at M2 gene loci, including sustained H3K4me3 at the Arg1 promoter, enabling faster and stronger M2 responses upon re‐exposure. This represents a form of trained immunity specific to the Type 2 lineage, with the clinical consequence that allergic patients exhibit heightened macrophage responses to low‐level allergen exposure that would be innocuous in nonatopic individuals [303].

### 4.4 TRM in Metabolic Inflammation: LAMs and Metaflammation

Metabolic inflammation, also termed metaflammation, is a chronic, low‐grade inflammatory state induced by nutrient excess and metabolic stress, rather than by pathogens or tissue injury. This paradigm was initially recognized in obesity, where adipose tissue expansion triggers macrophage infiltration and activation, but has since been extended to nonalcoholic fatty liver disease (NAFLD), NASH, Type 2 diabetes, and atherosclerosis. Unlike the acute, self‐limiting inflammation, metabolic inflammation persists for years or decades, driving progressive tissue damage through mechanisms that are qualitatively distinct from both PAMP‐driven and DAMP‐driven responses. This section focuses on the unique features of macrophage biology in metabolic inflammation, including the concept of LAMs, the TREM2‐centered metabolic sensing pathway, and the integration of nutritional and inflammatory signals.

#### 4.4.1 The LAM Signature

Single‐cell transcriptomic studies over the past decade have revealed that macrophages in metabolic tissues are not simply M1 or M2 polarized but instead adopt a distinct transcriptional state now termed LAMs [304]. LAMs were first identified in adipose tissue of obese mice and subsequently found in human obesity, NASH liver, and atherosclerotic plaques [305]. Their defining feature is the expression of a core set of genes involved in lipid handling and metabolic sensing, including *Trem2 *(triggering receptor expressed on myeloid cells 2), *Cd9*, *Gpnmb* (glycoprotein nonmetastatic melanoma protein B), *Lpl* (lipoprotein lipase), and multiple genes involved in lysosomal function and cholesterol metabolism [304, 306].

TREM2 has emerged as the master regulator of the LAM program. Originally identified as a receptor for phosphatidylserine and apolipoproteins involved in microglial function in Alzheimer's disease, TREM2 is upregulated on macrophages in obesity, NASH, and atherosclerosis [307, 308]. TREM2 signaling promotes phagocytosis of lipids and apoptotic cells, suppresses proinflammatory cytokine production, and supports metabolic adaptation to lipid‐rich environments [309]. *Trem2* deficiency in mice exacerbates metabolic inflammation: adipose tissue accumulates more crown‐like structures (clusters of macrophages surrounding dead or dying adipocytes) [310], hepatic steatosis progresses to more severe NASH [311], and atherosclerotic plaques become larger and more unstable [312]. Conversely, TREM2 agonism has been shown to limit metabolic inflammation in preclinical models [313], suggesting that LAMs represent a protective, metabolically adaptive response rather than a purely proinflammatory one.

The LAM signature also includes lysosomal lipid handling genes. Macrophages in metabolic tissues internalize large quantities of lipids through scavenger receptors such as CD36 and SR‐A [314]. These lipids are trafficked to lysosomes, where they are processed by lipases and presented to nuclear receptors such as PPARγ and LXR [315, 316]. Cholesterol efflux via ABCA1 and ABCG1 prevents lysosomal lipid accumulation [317]. When lipid influx exceeds efflux capacity, macrophages become foam cells, characterized by lipid‐laden lysosomes that impair cellular function [318]. Foam cell formation is a hallmark of atherosclerosis but also occurs in adipose tissue and liver in obesity. The LAM transcriptional program appears to represent an attempt to manage this lipid overload, upregulating genes involved in lysosomal acidification, lipid catabolism, and cholesterol export.

#### 4.4.2 The Integration of Metabolic and Inflammatory Signaling

A distinctive feature of metabolic inflammation is the direct crosstalk between metabolic pathways and inflammatory signaling within macrophages. Free fatty acids, particularly saturated fatty acids such as palmitate, activate TLR4 directly, providing a PAMP‐like signal from a metabolite [319]. This observation has led to the concept that metabolic inflammation represents a form of sterile inflammation where metabolites act as endogenous TLR4 ligands. However, the concentration of free fatty acids required to activate TLR4 in vitro is supraphysiological, suggesting that other mechanisms are also at play [320]. Ceramides, synthesized from saturated fatty acids, activate NLRP3 inflammasome and promote IL‐1β secretion [321]. Cholesterol crystals, formed when cholesterol concentration exceeds its solubility limit, activate NLRP3 through phagolysosomal damage, a mechanism identical to that in gout [231]. Oxidized phospholipids, generated from LDL oxidation, activate TLR2 and TLR4 and also serve as ligands for the scavenger receptor CD36 [322, 323].

ER stress represents another convergence point between metabolism and inflammation. Adipocyte and hepatocyte expansion in obesity causes ER stress, activating the unfolded protein response (UPR) [324]. The UPR sensor IRE1α activates JNK and NF‐κB pathways in adjacent macrophages, promoting proinflammatory cytokine production [324, 325]. This paracrine mechanism does not require the macrophage to directly sense any metabolite; the inflammatory signal is transmitted from stressed parenchymal cells [326]. ER stress also triggers the release of DAMPs such as HMGB1 from metabolically stressed cells, providing another indirect activation pathway [325, 327].

Mitochondrial dysfunction links metabolic overload to inflammasome activation. Excessive fatty acid delivery to macrophages overwhelms oxidative phosphorylation capacity, leading to mtROS production [328]. These ROS activate NLRP3 inflammasome, driving IL‐1β and IL‐18 secretion [329]. mtDNA released from damaged mitochondria also activates the cGAS–STING pathway, promoting Type I IFN responses that paradoxically can both limit and exacerbate metabolic inflammation depending on context [330]. This mitochondrial pathway is shared with sterile inflammation, but in metabolic inflammation the trigger is chronic nutrient excess rather than acute ischemia or crystal deposition.

#### 4.4.3 Tissue‐Specific Manifestations

The LAM program manifests differently across metabolic tissues, reflecting distinct local lipid environments. In adipose tissue, LAMs localize to crown‐like structures surrounding dead or dying adipocytes [305]. These LAMs express high levels of TREM2, CD9, and Gpnmb and are actively phagocytosing lipid droplets released from ruptured adipocytes [305, 331]. Adipose tissue LAMs suppress local T cell responses and promote adipocyte progenitor differentiation, suggesting a tissue‐reparative function [331]. Their abundance correlates with insulin resistance [332], but whether this represents a protective or pathogenic role remains debated.

In liver, Kupffer cells and recruited monocyte‐derived macrophages accumulate lipid droplets and express the LAM signature in NAFLD and NASH. Liver LAMs localize to areas of steatosis and lipotoxic injury [333]. They secrete proinflammatory cytokines including IL‐1β and TNF‐α, contributing to hepatocyte injury and fibrosis [334]. Single‐cell studies have identified a distinct TREM2‐positive subset in human NASH liver that is associated with disease severity [335, 336]. Unlike in adipose tissue, liver LAMs appear predominantly pathogenic, although their role in clearing lipid‐laden hepatocyte debris may also be protective in early disease stages [333].

In atherosclerotic plaques, macrophages become foam cells by ingesting oxLDL [337]. These cells express TREM2 and other LAM markers [304], and single‐cell sequencing has revealed remarkable heterogeneity within plaque macrophages, including inflammatory, resident‐like, and LAM subsets [338]. The LAM subset in plaques may represent a reparative population attempting to clear lipid debris [313], but in advanced plaques, LAMs contribute to plaque instability through MMP secretion [339]. The balance between lipid uptake, cholesterol efflux, and inflammatory activation determines whether LAMs protect or exacerbate disease.

### 4.5 Summary

The four inflammatory paradigms examined above demonstrate the remarkable functional diversity of TRMs. Sterile inflammation is driven by DAMPs from necrotic or stressed cells. Macrophages respond through the NLRP3 inflammasome, which acts as a metabolic sensor of cellular stress, with pyroptosis creating a self‐amplifying loop that can become chronic when resolution fails. Autoimmune inflammation is driven by self‐antigens and autoantibodies. Macrophages are activated through Fcγ receptors by immune complexes and serve as dominant APCs for restimulating self‐reactive T cells within target tissues. Type 2 inflammation is driven by helminths and allergens, directing macrophages toward M2 polarization via the IL‐4/IL‐13 STAT6 axis, with Arg‐1 replacing iNOS and effector functions focused on tissue repair and fibrosis. Metabolic inflammation is driven by chronic nutrient excess, inducing a distinct macrophage state termed LAMs, defined by TREM2, CD9, and Gpnmb expression, representing a metabolically adaptive response to lipid overload.

Despite their distinct triggers and effector programs, common themes emerge across these four paradigms. All rely on signal integration to calibrate response intensity. All include built‐in resolution mechanisms that restore homeostasis when the inciting stimulus is removed. All can transition to chronicity through self‐amplifying loops when resolution fails. And all exhibit tissue‐specific variations that reflect the anatomical context of the affected organ (Table 1).

**TABLE 1:** Comparison of macrophage features across inflammatory paradigms.

Feature	Infectious inflammation (sepsis)	Sterile inflammation	Autoimmune inflammation	Type 2 inflammation	Metabolic inflammation
Primary trigger	PAMPs (LPS, flagellin, bacterial DNA)	DAMPs (HMGB1, ATP, MSU crystals, mtDNA)	Self‐antigens + autoantibodies (immune complexes)	Helminths, allergens	Nutrient excess (saturated FAs, cholesterol crystals)
Core signaling pathway	TLR/MyD88/NF‐κB; noncanonical inflammasome (Caspase‐11 via OMVs) [190, 191]	NLRP3 inflammasome (acts as a metabolic sensor for K^+^ efflux, lysosomal damage) [225, 227]	FcγR cross‐linking → Syk/NF‐κB; CD80/CD86 costimulation [260, 266]	IL‐4/IL‐13 → STAT6; arginine metabolic fork (Arg‐1 vs. iNOS) [287, 294]	Metabolic stress sensors: TREM2 signaling, paracrine ER stress signals, mtROS/NLRP3 [306, 323, 328]
Macrophage polarization	Dynamic: M1 (early) → mixed/immunoparalysis (late) [156]	M1‐like (often transient, primed for resolution) [245]	M1‐like (e.g., osteoclasts in RA); interferon‐primed state [273, 277]	M2 (alternative activation) [286]	Lipid‐associated macrophages (LAM; TREM2^+^, CD9^+^, Gpnmb^+^) [303, 304]
Unique effector mechanism	Pyroptosis and cytokine storm (IL‐1β, TNF‐α); immunoparalysis via PD‐L1 upregulation [194]	Self‐amplifying loop via pyroptosis‐mediated DAMP release (HMGB1, ATP) [239, 240]	Osteoclastogenesis (bone erosion in RA); Type I IFN production (SLE) [273, 276]	Arg‐1‐dependent polyamine/proline synthesis for fibrosis; Chitinase production (Ym1) [291, 296, 298]	TREM2‐mediated phagocytosis and anti‐inflammatory lipid handling; Foam cell formation when overloaded [308, 317]
Adaptive immunity involvement	Required (macrophages bridge innate and adaptive immunity) [159, 160]	Minimal (predominantly innate‐driven) [258]	Dominant (macrophages are primary APCs for reactivating self‐reactive T cells) [269]	Moderate (Th2 cells provide IL‐4/IL‐13 for M2 polarization) [289]	Minimal
Temporal pattern	Acute, transitioning to persistent inflammation–immunosuppression paradox [203, 204]	Acute to subacute, can become self‐sustaining [242]	Chronic, progressive [265]	Chronic with acute flares (asthma exacerbations) [300]	Chronic, slowly progressive (“metaflammation”)
Driver of chronicity	Metabolic lockdown (Warburg effect), mitochondrial dysfunction, and epigenetic imprinting [156, 210, 216]	Persistent DAMP release due to failed resolution (e.g., impaired efferocytosis) [241, 242]	Persistent self‐antigen presentation; FcγR‐mediated feed‐forward loop [256]	Epigenetic imprinting of primed M2 state (trained immunity) [302]	Persistent nutrient overload overwhelming adaptive lipid handling [313, 317]

## 5 TRM in Cancer: The Malignant Hijacking of Macrophage Plasticity

Intratumoral TAMs are crucial components of the tumor microenvironment and play a significant role in tumor development and drug resistance by creating an immunosuppressive microenvironment. Unlike the acute dysfunction seen in lethal infections, where macrophage plasticity drives a temporary hyperinflammatory storm followed by immunoparalysis, TAMs undergo a chronic, sustained reprogramming process. In the tumor, a nonhealing “chronic wound,” TRMs and recruited monocytes are not simply overwhelmed but are continuously educated and co‐opted by microenvironmental signals, ultimately becoming accomplices of tumor growth, invasion, and immune escape. Strikingly, this chronic subversion shares core molecular programs with the septic immunoparalytic state, including metabolic reprogramming, epigenetic imprinting, and immune checkpoint upregulation. This mechanistic convergence suggests that lessons learned from one disease context can illuminate the other.

### 5.1 Origin and Education of TAMs: A Chronically Reprogrammed Population

TAMs constitute one of the most abundant and functionally complex immune cell populations within the solid tumor microenvironment [340]. Unlike macrophages in acute infection, which either return to homeostasis or sink into immunoparalysis following a brief but intense period of activation, TAMs undergo a protracted process of continuous shaping and profound reprogramming imposed by the tumor microenvironment. Understanding the heterogeneity of TAM origins and the mechanisms underlying their education is a fundamental prerequisite for unraveling their multifaceted tumor‐promoting functions and for developing effective interventional strategies.

The cellular origins of TAMs are not singular but rather exhibit remarkable heterogeneity and disease specificity. It has long been held that TAMs are predominantly derived from circulating Ly6C^hi^ classical monocytes, which are continuously recruited to tumor tissue through chemotactic axes such as CCL2–CCR2 and subsequently differentiate into mature TAMs under the influence of the local milieu [341]. This monocyte recruitment model has been extensively validated in the majority of solid tumor models, and the existence within tumor tissue of abundant monocyte‐derived TAMs at various stages of differentiation provides further support for this paradigm. However, with the widespread application of lineage‐tracing technologies and single‐cell omics, it is now firmly established that in many tumor types, particularly during the early stages of tumorigenesis, tissue‐resident, embryo‐derived macrophages also constitute an important source of TAMs [342]. In pancreatic ductal adenocarcinoma, for example, embryo‐derived pancreatic TRMs become activated and undergo in situ proliferation at the very initiation of neoplasia, representing the major component of early TAMs [343]. In glioma, microglia, rather than recruited monocytes, account for a substantial proportion of TAMs [344]. Likewise, in hepatocellular carcinoma and lung adenocarcinoma, Kupffer cells and AMs have been demonstrated to undergo in situ phenotypic conversion in response to tumor microenvironmental cues and to directly transform into TAMs with tumor‐promoting functions [345, 346]. This dual origin, in situ reprogramming of embryo‑derived resident macrophages and continuous recruitment of monocytes, renders TAMs a highly heterogeneous population. As the disease progresses, TAMs derived from these two sources may exhibit both overlapping and distinct functional roles, constructing a finely regulated macrophage ecosystem within the tumor microenvironment. Furthermore, with increasing tumor burden and the formation of necrotic cores, monocyte‑derived TAMs gradually become dominant, and their distribution in necrotic regions together with their adaptation to hypoxic signals positions them as the principal functional executors during advanced tumor progression [347].

Once recruited to the tumor site, TAMs undergo a process of profound education by the tumor microenvironment that diverts them away from normal immune defense programs and transforms them into multifunctional accomplices serving tumor growth and progression [348]. This educational process is orchestrated by a constellation of signals secreted collectively by tumor cells, stromal cells, and immune cells, with core mechanisms encompassing metabolic reprogramming, remodeling of transcription factor networks, and the establishment of epigenetic imprints [349]. Hypoxia, one of the most pervasive physicochemical hallmarks of solid tumors, serves as a central signal driving TAM education. Under low oxygen tension, hypoxia‑inducible factor HIF‑1α is stabilized and undergoes nuclear translocation, initiating the transcription of proangiogenic factors such as VEGF and PDGF while upregulating the expression of glycolytic enzymes, thereby steering TAMs toward proangiogenic and immunosuppressive phenotypes, and enabling TAMs to resist cell death [350, 351]. Lactate, the end product of aerobic glycolysis by tumor cells, accumulates in the tumor interstitium at concentrations tens of times higher than those in normal tissues. TAMs take up lactate via monocarboxylate transporters [352]. Lactate not only serves as an energy substrate directly fueling oxidative phosphorylation and M2‑associated gene expression but also induces the expression of canonical M2 markers such as Arg‑1 and VEGF through ERK–STAT3 signaling [353]. Concomitantly, via histone lactylation, lactate directly regulates the transcription of genes associated with tissue repair and immunosuppression, achieving an epigenetic lock on their effector functions [354].

The cytokine network within the tumor microenvironment constitutes the critical humoral signaling system driving TAM education. IL‑4 and IL‑13, secreted by tumor cells and Th2 cells, signal through STAT6 to drive TAM polarization toward an M2‑like phenotype, inducing the expression of markers such as CD206, Arg‑1, and CCL17 [355]. IL‑10, abundantly produced by tumor and stromal cells, acts through STAT3 to suppress the production of proinflammatory cytokines, impair antigen‑presenting capacity, and upregulate the expression of immune checkpoint molecules [356]. CSF‑1 (also known as M‑CSF) is essential for macrophage recruitment, survival, and proliferation; in multiple solid tumors, the sustained activation of the CSF‑1/CSF‑1R axis not only maintains TAM viability but actively drives their differentiation toward immunosuppressive and prometastatic phenotypes [357]. TGF‑β is one of the most abundant immunosuppressive factors in the tumor microenvironment, with sources including tumor cells, Treg cells, and TAMs themselves [358, 359]. Lactate potentiates TGF‐β/SNAIL signaling in macrophages by upregulating TGFBR1/2 expression and enhancing Smad2/3 phosphorylation, thereby linking metabolic cues to the canonical TGF‐β pathway to reinforce M2 polarization [360].

Under the chronic, long‑term stimulation imposed by the tumor microenvironment, the transcriptional and epigenetic landscape of TAMs undergoes profound alterations. Single‑cell omics have revealed that the transcriptional states of TAMs do not conform to a simple M1/M2 dichotomy but rather constitute a continuous spectrum encompassing dozens of distinct subsets. In colorectal cancer, single‐cell RNA sequencing identified distinct TAM subpopulations including SPP1^hi^ TAMs with proangiogenic and immunosuppressive functions, and C1QC^hi^ TAMs enriched for complement and phagocytosis‐related gene programs [361]. In hepatocellular carcinoma, TREM2^hi^ TAMs were identified as a major immunosuppressive subset that drives CD8^+^T cell exhaustion through Galectin‐1 secretion [362]. In gliomas, Mo‐TAMs were found to localize to peri‐necrotic regions and display a hypoxic gene signature characterized by ADM and VEGF expression [363]. In pancreatic ductal adenocarcinoma, an IL‐1β^+^TAM subset was identified that fuels pathogenic inflammation and tumor progression [364]. In breast cancer, FOLR2^+^ TRMs were identified as a distinct TAM population located in the perivascular tumor stroma, where they interact with CD8^+^T cells and are positively correlated with better patient survival [365]. These examples underscore the transcriptional and functional diversity of TAMs across tumor types and anatomical niches within individual tumors.

This complex heterogeneity arises precisely because macrophages of different origins are subjected to diverse combinations of local signals, each combination initiating distinct reprogramming trajectories [366]. Chromatin remodeling complexes, such as the BAF complex, play a critical role in shaping the chromatin accessibility landscape of TAMs, and their inhibition leads to widespread changes in gene expression [367]. In line with this, ARID1A‐deficient TAMs exhibit increased promoter accessibility at IFN‐stimulated genes, directly linking chromatin state alterations to functional gene output [368]. At the posttranslational level, lactate‐driven acetylation of the transcription factor C/EBPα has been shown to suppress CD74 expression and impair antigen‐presenting function in TAMs [369]. In parallel, TAMs upregulate the expression of multiple immune checkpoint molecules, including PD‑L1, PD‑L2, VISTA, and B7‑H4 [370]. These molecules directly inhibit antitumor T cell responses by engaging their cognate receptors on T cells, thereby serving as key effector molecules in maintaining the immunosuppressive tumor microenvironment.

In summary, the origin and education of TAMs constitute a dynamic process cooperatively driven by multidimensional signals from the tumor microenvironment. The in situ reprogramming of embryo‑derived resident macrophages and the continuous recruitment of circulating monocytes jointly establish the cytological diversity of the TAM pool, while hypoxia, metabolites, cytokine networks, and epigenetic remodeling progressively drive their functional conversion. This multi‑source, multi‑mechanism functional education renders TAMs the most phenotypically plastic immune cell population within tumors. Yet exactly this reprogrammed state, locked in place by epigenetic mechanisms, also provides a therapeutic window of opportunity for erasing pathogenic imprints through epigenetic drugs or metabolic interventions. Moreover, the upregulation of immune checkpoints, impaired phagocytic function, and M2‑like polarized state that emerge during the chronic education process described above bear striking phenotypic resemblance to the macrophage immunoparalysis observed in the late stage of lethal infectious diseases further highlighting the profound value of cross‑comparing macrophage biology between these two seemingly disparate disease processes.

### 5.2 TAMs in Tumor Promotion: Orchestrating an Immunosuppressive Niche

**FIGURE 5:**
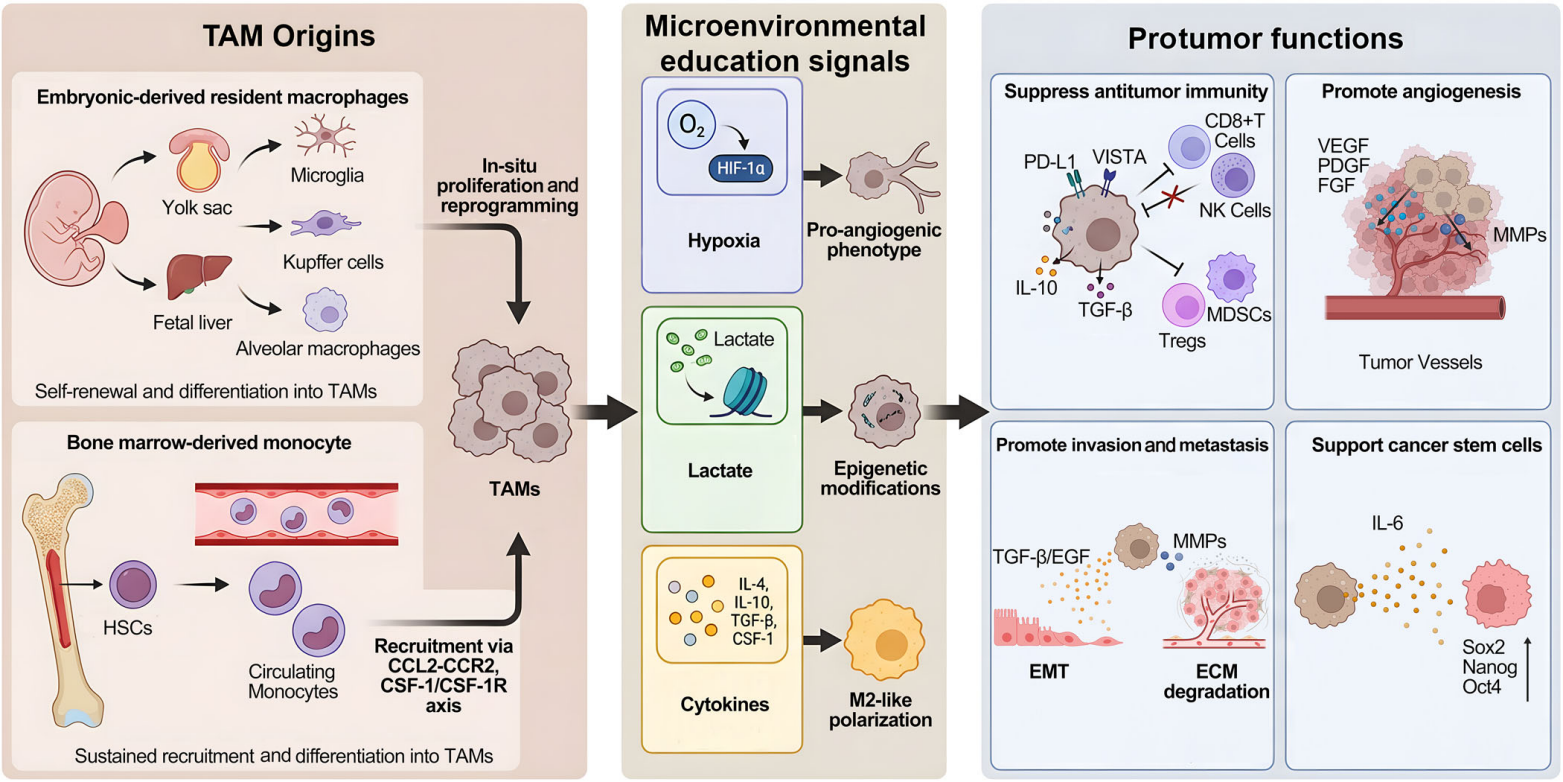
Origins, microenvironmental education, and protumor functions of tumor‐associated macrophages. The diagram illustrates the three‐stage lifecycle and functional remodeling of TAMs within the tumor microenvironment. The left panel depicts the dual origins of TAMs, which arise from both embryo‐derived tissue‐resident macrophages (e.g., microglia, Kupffer cells, alveolar macrophages) via in situ proliferation and reprogramming, and from bone marrow‐derived circulating monocytes recruited through the CCL2–CCR2 and CSF‐1–CSF‐1R axes. The middle panel highlights the pivotal microenvironmental education signals that sustain TAM reprogramming, including hypoxia (driven by HIF‐1α stabilization), lactate (which induces histone lactylation and epigenetic locks), and a cytokine milieu enriched in IL‐4, IL‐10, TGF‐β, and CSF‐1. The right panel summarizes the resulting protumorigenic functions of educated TAMs across four interconnected axes: they suppress antitumor immunity by secreting IL‐10/TGF‐β and upregulating PD‐L1/VISTA while recruiting Tregs and MDSCs; they promote angiogenesis via the HIF‐1α‐driven release of VEGF, PDGF, FGF, and MMPs; they facilitate invasion and metastasis by secreting MMPs and TGF‐β/EGF to degrade the extracellular matrix and inducing EMT; and they support cancer stem cells (CSCs) by activating IL‐6/JAK/STAT3, Notch, and Wnt signaling to upregulate Sox2, Nanog, and Oct4. Collectively, TAMs serve as central drivers of immune evasion, angiogenesis, metastatic spread, and therapy resistance. (Drafted by NanoBanana Pro 4K and subsequently manually redrawn and finalized by the author using BioRender.)

Among the multiple protumor functions of TAMs, the construction of an immunosuppressive niche stands as the most fundamental and best‐characterized. Through a coordinated array of soluble mediators, membrane‐bound checkpoints, and cellular recruitment, TAMs systematically dismantle antitumor immunity (Figure 5).

#### 5.2.1 Inhibiting Antitumor Immune Response

A central mechanism by which TAMs promote tumor progression is the establishment of a profoundly immunosuppressive microenvironment that dismantles antitumor immune responses. This is achieved through coordinated mechanisms including secretion of immunosuppressive factors, expression of immune checkpoint ligands, and recruitment of immunosuppressive cell populations. Together, these drive effector T cells and NK cells into functional exhaustion or physical exclusion, enabling immune evasion. This multilayered immunosuppressive program bears striking molecular similarity to the macrophage immunoparalysis observed in late‐stage sepsis, underscoring the value of cross‐comparing macrophage biology across these two disease states.

Within the tumor microenvironment, TAMs represent a major source of immunosuppressive cytokines, directly suppressing the functions of effector T cells and NK cells through paracrine signaling [371]. IL‑10, abundantly secreted by TAMs, is one of the core cytokines that construct the immunosuppressive ecosystem. Through activation of the STAT3 signaling pathway, IL‑10 inhibits the differentiation, maturation, and antigen‑presenting capacity of dendritic cells, thereby indirectly blocking T cell priming [372]. Concurrently, IL‑10 acts directly on CD8^+^T cells to suppress their ability to produce effector cytokines such as IFN‑γ, TNF‑α, and IL‑2, while inducing the expression of coinhibitory molecules that drive T cells toward exhaustion [373]. TGF‐β is another critical suppressive factor secreted by TAMs. It directly inhibits the cytotoxic function of CD8^+^ T cells by suppressing the expression of perforin, granzyme A, granzyme B, Fas ligand, and IFN‐γ, thereby impairing CTL‐mediated tumor clearance [374]. TGF‐β also suppresses NK cell function. Recent evidence demonstrates that SMAD4‐deficient human NK cells resist TGF‐β‐mediated inhibition and retain their cytotoxicity and cytokine production capacity, confirming that the TGF‐β/SMAD4 axis is a central mechanism by which TGF‐β constrains NK cell antitumor activity [375]. At the metabolic level, TAMs highly express Arg‑1, a hallmark enzyme of M2‑like polarization. By depleting l‑arginine from the microenvironment, Arg‑1 leads to downregulation of the CD3ζ chain in the TCR complex, directly impairing T cell activation and proliferation [376]. Indoleamine 2,3‑dioxygenase is another key metabolic enzyme expressed by TAMs, which catalyzes the degradation of tryptophan along the kynurenine pathway. The resulting local depletion of tryptophan and the accumulation of downstream catabolites together suppress T cell effector function [377].

TAMs also directly engage inhibitory receptor pathways by expressing immune checkpoint ligands. PD‐L1, the most extensively characterized, is upregulated on TAMs in response to hypoxia, IFN‐γ, and CSF‐1, and its binding to PD‐1 on CD8^+^ T cells drives diminished proliferation, reduced effector cytokine production, and eventual exhaustion [358]. However, it has also been found that PD‐L1‐expressing TAMs are immunostimulatory and improve clinical outcome in human breast cancer [378]. Beyond PD‐L1, TAMs express VISTA and TIM‐3, which is associated with resistance to anti‐PD‐1/PD‐L1 therapy, and B7‐H4, which inhibits T cell proliferation and IL‐2 production [379]. CD47 on TAMs engages SIRPα on phagocytes to transmit a “don't eat me” signal; disrupting this axis robustly promotes macrophage‐mediated phagocytosis [380], and USP2 inhibition reduces CD47 expression to enhance antitumor immunity [381].

Beyond directly suppressing effector cell function, TAMs actively recruit additional immunosuppressive cell populations into tumor tissue through the secretion of chemokines, thereby constructing a multicellular, synergistic immunosuppressive network. TAMs selectively recruit CCR4^hi^FoxP3^+^ Treg cells into the tumor bed by secreting chemokines such as CCL22 and CCL17 [382, 383]. Once recruited, Treg cells in turn release IL‑10 and TGF‑β, further fortifying the local immunosuppressive milieu and forming a positive feedback loop with TAMs that continually amplifies the immunosuppressive effect. Meanwhile, through the secretion of CCL2, TAMs not only sustain their own continuous recruitment but also attract CCR2^+^ monocyte‑derived myeloid‑derived suppressor cells (MDSCs) into the tumor [384]. The newly recruited MDSCs, through the production of mediators such as Arg‑1, reactive oxygen species, and reactive nitrogen species, collaborate with TAMs to suppress the effector function of CD8^+^ T cells and the cytotoxic activity of NK cells [385]. In certain tumor types, IL‐6 derived from both tumor cells and TAMs promotes Th2‐cell differentiation, shifting the helper function of CD4^+^ T cells from an antitumor Th1 bias toward a tumor‐promoting Th2 phenotype, thereby indirectly suppressing CTL activation [386]. This TAM‑orchestrated immunosuppressive cellular network renders the tumor microenvironment a highly immune‑privileged site. Recent studies further uncover novel dimensions of TAM‑mediated immunosuppressive regulation. In gastric adenocarcinoma, FPR3 was identified as a novel marker of immunosuppressive TAMs. FPR3 upregulates FZD7 and CCDC88C, thereby activating the Wnt/PCP pathway and downstream JNK signaling to sustain the immunosuppressive function and proliferation of TAMs [387]. In non‑small cell lung cancer, chemotherapy resistance can lead to aberrant accumulation of extracellular adenosine. Adenosine activates the A2A receptor on the TAM surface, driving the PKA/mTORC signaling pathway and inducing metabolic reprogramming and high IDO1 expression in TAMs. The activated TAMs massively deplete local tryptophan, directly impairing T cell function and mediating cross‑resistance to immunotherapy [388]. In melanoma models, IFN‑γ was found to drive the remodeling of the mitochondrial complex IV subunit NDUFA4 in TAMs, which activates the cGAS‑STING pathway to produce CXCL9/10, thereby recruiting and activating NK cells and CD8^+^ T cells [389]. This reveals a novel mechanism in which an intrinsic metabolic–immune axis in TAMs initiates antitumor immunity.

In summary, TAMs collaboratively construct a formidable immune barrier that enables tumors to evade immune attack through three interconnected mechanisms: the secretion of immunosuppressive factors, the expression of immune checkpoint molecules, and the recruitment of immunosuppressive cells. This multifunctional suppressive network not only explains why TAM enrichment is strongly correlated with immunotherapy resistance and poor prognosis, but also provides clearly defined molecular targets for developing therapeutic strategies that aim to target TAMs to enhance antitumor immunity. Viewed through the lens of septic immunoparalysis, this sustained checkpoint molecule upregulation and state of effector cell inhibition exhibited by TAMs represents an extreme extension of the inflammation–immunosuppression spectrum within a chronic tumor environment.

#### 5.2.2 Enhancing Tumor Angiogenesis

Tumor growth and metastasis are critically dependent upon an adequate supply of oxygen and nutrients, rendering angiogenesis a rate‐limiting step in tumor progression. Within the tumor microenvironment, TAMs function as central orchestrators of pathological angiogenesis. Through the secretion of a repertoire of proangiogenic factors, the expression of vascular‐modulating enzymes, and direct participation in vascular structural remodeling, they hijack the normally tightly controlled angiogenic program and convert it into a sustained, disorganized, and functionally aberrant tumor vascular network. This process not only provides the material conditions essential for tumor survival but also pre‐establishes metastatic conduits for the distant dissemination of tumor cells. Hypoxic tumor regions drive TAMs to acquire a proangiogenic phenotype characterized by upregulation of VEGF and other angiogenic factors [363]. This TAM‐mediated angiogenic response represents a key component of the maladaptive wound‐healing program that sustains tumor progression and shapes the immunosuppressive microenvironment.

TAMs are a critical source of proangiogenic factors within the tumor microenvironment [390]. Under the combined stimulation of hypoxia, an acidic milieu, and tumor‐derived cytokines, TAMs secrete large quantities of VEG family members [391]. VEGF‐A is the most potent and most thoroughly characterized proangiogenic factor among them. TAM‐derived VEGF‐A binds to VEGFR‐2 on the surface of vascular endothelial cells, activating their proliferation, migration, and tube formation and inducing the sprouting of new blood vessels from pre‐existing vascular beds [392]. However, the tumor vessels driven by TAMs are severely abnormal in both structure and function: they exhibit uneven luminal diameters, tortuous and chaotic trajectories, and incomplete pericyte coverage, resulting in heterogeneous blood perfusion and markedly increased vascular permeability [393]. This aberrantly leaky vascular network not only generates elevated interstitial fluid pressure, impeding drug delivery, but also provides structural convenience for tumor cell intravasation. Beyond VEGF‐A, TAMs also secrete placental growth factor, which amplifies the proangiogenic effects of VEGF‐A by activating VEGFR‐1 signaling and is particularly critical for the initial vascular sprouting in certain tumor types [394]. TAMs also contribute to PDGF‐BB/PDGFR‐β‐mediated pericyte recruitment through a reciprocal paraCrine loop critical for glioblastoma progression [395]. Although this recruitment is intended to promote vascular maturation, in the tumor context it is incompletely executed and instead exacerbates aberrant vascular remodeling. Members of the fibroblast growth factor family, including FGF2, are also secreted by TAMs and cooperate with VEGF signaling to potentiate endothelial cell proliferation and proangiogenic responses [396, 397].

In the persistently hypoxic microenvironment of tumors, HIF‐1α constitutes the critical transcriptional hub governing the proangiogenic functions of TAMs. Under hypoxic conditions, HIF‐1α stably accumulates in TAMs and undergoes nuclear translocation, where it directly activates the transcription of proangiogenic factors such as VEGF‐A, PDGF, and FGF [398]. Beyond these canonical targets, HIF‐1α also upregulates the expression of genes with potential vascular‐modulating and pericyte‐recruiting functions, including CXCL8 (IL‐8), CXCL12 (SDF‐1), and Angiopoietin‐2, thereby constructing a complex proangiogenic transcriptional network [399, 400]. Another key function of TAMs is the secretion of vascular‐remodeling enzymes, which directly participate in the degradation and restructuring of the ECM, providing spatial conditions for endothelial cell migration and tube formation. TAMs represent an important source of MMPs. By degrading collagen and other components of the basement membrane and interstitial matrix, these enzymes not only release matrix‐sequestered proangiogenic factors but also clear physical barriers for endothelial sprouting and migration [401]. Furthermore, urokinase‐type plasminogen activator (uPA) expressed by TAMs activates plasmin, further participating in the cascade of matrix degradation and vascular remodeling [402]. Members of the cathepsin family also play important roles in TAM‐mediated angiogenesis by directly contributing to matrix degradation and by activating other protease precursors, indirectly facilitating the invasion and extension of nascent vessels [403].

In recent years, the development of single‐cell transcriptomics has enabled the precise molecular identification and definition of specific TAM subsets that are dedicated to proangiogenic functions. Studies across multiple human tumor types have revealed the existence of a TAM subset characterized by high expression of genes such as SPP1 (encoding osteopontin) and TREM2, whose transcriptional program is significantly enriched in pathways related to angiogenesis, hypoxia response, and ECM remodeling [404, 405]. In breast cancer, single‐cell transcriptional atlases have identified an Angio‐TAM subset defined by high expression of proangiogenic genes including VEGFA, FN1, and IL8, and its abundance is associated with patient prognosis and immunotherapy response [406]. In non‐small cell lung cancer, single‐cell RNA sequencing has identified CXCL10^+^, AGER^+^, and FABP4^+^ macrophage subclusters that are implicated in angiogenesis through their interactions with endothelial cells. Aberrant sphingolipid metabolism in the TME was shown to drive M2 polarization and angiogenesis via MIF‐CD74 signaling [407]. In hepatocellular carcinoma, an NFKBIZ^hi^ macrophage subset is characterized to be predominantly enriched in immunotherapy nonresponders. These macrophages exhibit a hypoxia‐driven phenotype characterized by the secretion of VEGFA and HBEGF, which cooperatively promote tumor angiogenesis, together with elevated expression of the inflammatory chemokines CXCL2, CXCL3, and CXCL8 that reinforce an immunosuppressive and protumorigenic microenvironment [408]. In spinal ependymomas, single‐cell transcriptomic and epigenomic analyses have identified a CD44^+^ TAM subset characterized by high VEGFA expression and TEAD1‐driven transcriptional programming, which is significantly enriched for angiogenesis pathways and associated with worse clinical prognosis [409].

In summary, TAMs cooperatively drive the formation of an aberrant tumor vascular network through the secretion of proangiogenic factors, activation of transcriptional programs in response to hypoxic signals, and release of matrix‐degrading enzymes. It is further worth reflecting that macrophages similarly participate in the pathological processes of vascular permeability dysregulation and tissue perfusion impairment during both the hyperinflammatory and immunoparalysis phases of inflammation. Although the outcomes differ, diffuse endothelial injury in infection versus focal pathological sprouting in tumors, the shared molecular basis through which macrophages modulate the vasculature, via VEGF and MMPs in both scenarios, constitutes an important common thread linking vascular pathology in inflammation and cancer.

#### 5.2.3 Facilitating Invasion and Metastasis

TAMs play a critical facilitating role at nearly every step of the metastatic cascade, from assisting local invasion and intravasation to preconditioning the premetastatic niche and supporting colonization of disseminated tumor cells. Through dynamic interactions with tumor cells and stromal cells, TAMs convert the physiological functions of macrophages in tissue repair and wound healing into drivers of metastatic progression. This contrasts sharply with the immunoparalysis seen in late‐stage lethal infections, where macrophages lose their pathogen‐clearing capacity. In tumors, TAMs are not functionally paralyzed but are actively reprogrammed to assist tumor cell spread.

At the primary tumor site, TAMs are key regulators that drive the acquisition of invasive capacity by tumor cells and their breaching of tissue barriers. The starting point of this process is the epithelial–mesenchymal transition (EMT), whereby tumor cells lose epithelial polarity, intercellular junctions, and basement membrane anchorage, and instead acquire the invasive and migratory capabilities of mesenchymal cells. TAMs secrete EMT‐inducing cytokines such as TGF‐β, EGF, HGF, and TNF‐α, which activate transcription factor families including Snail, Twist, and ZEB within tumor cells, directly driving the EMT program [410]. Concurrently, IL‐6 secreted by TAMs upregulates the expression of these transcription factors via STAT3 signaling, further consolidating the mesenchymal phenotype of tumor cells [411]. Tumor cells that have undergone EMT not only display markedly enhanced invasive capacity but also acquire stem cell‐like characteristics and resistance to apoptosis, enabling them to survive independently after detaching from the primary mass [412]. TAMs directly participate in ECM degradation through the secretion of multiple proteases, clearing the physical barriers for local tumor cell invasion. TAMs are a core source of MMPs, which efficiently degrade Type IV collagen in the basement membrane and Type I and III collagens in the interstitial matrix, creating gaps in the basement membrane and providing passageways for tumor cell egress [401]. Beyond MMPs, TAMs also secrete proteases such as cathepsins and uPA, achieving sustained degradation and remodeling of the ECM through multienzyme synergy [402]. This TAM protease‐dependent disruption of ECM architecture not only furnishes physical conduits but also releases matrix‐sequestered growth factors such as EGF, FGF, and TGF‐β during the degradation process, generating a positive feedback loop that promotes sustained tumor cell invasion [398].

Once tumor cells successfully invade the stroma, the next critical rate‐limiting step is intravasation, entry into the vascular lumen to achieve dissemination. TAMs play a critical guiding and assisting role in this process. Within tumor tissue, TAMs are frequently densely distributed around blood vessels, forming so‐called “perivascular TAMs” [413]. These perivascularly localized TAMs secrete chemokines such as CXCL12 and EGF, guiding tumor cells expressing high levels of CXCR4 and EGFR to directionally migrate toward the perivascular region [414]. At local sites of the vascular basement membrane, TAMs establish direct contact with tumor cells and assist them in breaching the vascular barrier [415]. More directly, transient direct physical contacts can form between tumor cells and TAMs, structures composed of invadopodia and intercellular junctions, that provide physical guidance for the directed migration and intravasation of tumor cells [416, 417]. Within the vasculature, TAMs can accompany tumor cell clusters to form microemboli, protecting circulating tumor cells (CTCs) from hemodynamic shear stress and anoikis by providing growth factors and contact‐dependent survival signals, thereby significantly enhancing their survival rate [418]. Moreover, TAMs can transmit specific signaling pathway activation capacities to tumor cells, enhancing their mechanical resistance and survival adaptability in the circulation [419].

Another core mechanism by which TAMs promote metastasis is the preconditioning of the premetastatic niche in distant organs, rendering it suitable for the colonization and growth of disseminated tumor cells. TAMs play a central role in the preconditioning of the premetastatic niche, a process that fundamentally reshapes our understanding of metastasis as a primary tumor‐driven, organ‐specific preparation rather than random dissemination [420]. At the primary site, TAMs secrete soluble factors including VEGF, TNF‐α, and TGF‐β into the circulation, which upregulate adhesion molecules on vascular endothelial cells in distant organs, thereby enhancing the capture of subsequently arriving tumor cells. More specifically, TAM‐derived EVs carry cargoes such as integrins, miRNAs, and growth factors that are internalized by resident cells of distant organs, preactivating proinflammatory and proadhesive gene programs [421, 422]. Upon reaching the target metastatic organ, TRMs are “corrupted” by tumor‐derived signals. For instance, AMs express TGF‐β2 that binds to receptors on disseminated cancer cells (DCCs), sustaining signaling pathways that maintain DCCs in a dormant state [423]. Concurrently, TRMs secrete chemokines to recruit Ly6C^hi^ monocytes, which then differentiate into metastasis‐associated macrophages (MAMs) within metastatic lesions. These MAMs support DCCs to escape from dormancy and initiate proliferation by providing survival factors such as WNT ligands, Notch signals, and TGF‐β [424]. Simultaneously, MAMs re‐establish vascular networks through the secretion of VEGF and MMPs, furnishing nutritional support for the transition from micrometastases to clinically detectable metastatic foci [425]. It is noteworthy that in the immunoparalytic state experienced by macrophages during late‐stage sepsis, the loss of phagocytic and antigen‐presenting capacity renders the host unable to clear disseminated pathogens; in stark contrast, during tumor metastasis, TAMs are not paralyzed but are instead actively reprogrammed into principal coordinators orchestrating the construction of the premetastatic niche [422]. This extreme divergence in the functional state of the same immune cell type in different diseases represents a profound illustration of macrophage plasticity.

Recent research advances have further uncovered noncanonical mechanisms by which TAMs promote tumor invasion and metastasis. Recent evidence indicates that autophagic TAMs promote TGF‐β1 secretion and induce EMT in tumor cells. In lung adenocarcinoma, TAM autophagy upregulates the FUT4/p‐ezrin pathway in an ATG5‐dependent manner, leading to increased TGF‐β1 release and enhanced invasiveness of cocultured cancer cells [426]. In lung adenocarcinoma, tumor‐derived exosomal miR‐19b‐3p has been shown to target PTPRD in TAMs, leading to STAT3 activation and M2 polarization, thereby promoting tumor metastasis [427]. In osteosarcoma, researchers discovered that TAM‐derived CCL18 upregulates UCA1 expression via EP300, thereby activating the Wnt/β‐catenin signaling pathway to promote osteosarcoma cell proliferation, migration, and lung metastasis [428]. Besides, TAMs serve as an acetate reservoir in the tumor microenvironment: cancer cell‐derived lactate activates the lipid peroxidation‐ALDH2 pathway in TAMs to generate and secrete acetate, which is then taken up by cancer cells to synthesize acetyl‐CoA, epigenetically upregulating TWIST2 expression via histone acetylation and thereby promoting HCC metastasis [429].

In summary, TAMs comprehensively participate in every link of the tumor metastatic cascade through the induction of EMT, degradation of the ECM, guidance of intravasation, protection of CTCs, and construction of both premetastatic and postmetastatic niches. This multistep, all‐round involvement makes TAMs a highly promising target in antimetastatic therapy. From a cross‐disease perspective, the collapse of macrophage phagocytic and clearance functions in the septic immunoparalysis, and the functional hijacking of TAMs reprogrammed to actively facilitate tumor cell dissemination and colonization described in this section, constitute landmark examples of the polarized extremes of macrophage plasticity, profoundly revealing how the same cell type can deliver diametrically opposite functional outputs in different pathological environments.

#### 5.2.4 Supporting CSCs

CSCs are a small subpopulation of cells within tumors that possess the capacity for self‐renewal and multilineage differentiation, and are considered the root of tumor initiation, maintenance, recurrence, and metastasis. In contrast to the traditional view that all tumor cells are equally endowed with proliferative capacity, the CSC model posits that only this specific cellular subset can sustain long‐term tumor growth, while the majority of tumor cells undergo limited divisions before committing to terminal differentiation. This concept has profoundly reshaped the design rationale of cancer therapeutic strategies: any treatment that fails to eradicate CSCs, even if it dramatically reduces tumor bulk, will ultimately face fatal recurrence. Within the tumor microenvironment, TAMs are key supporting cells that maintain the CSC niche. Through paracrine signaling, direct cell–cell contact, and adaptive support of CSC metabolic demands, TAMs provide CSCs with a “breeding ground” and a “protective sanctuary” that shelters their stemness characteristics. From an evolutionary perspective, this function represents a pathological distortion of the core macrophage function of supporting stem cell niches during normal tissue repair and development. The molecular toolkit that macrophages originally employ to support tissue regeneration is fully preserved but maliciously redirected in tumors to sustain the unbridled expansion of CSCs.

TAMs secrete a variety of soluble factors that directly activate key stemness‐maintaining signaling pathways in CSCs, constituting the core molecular basis of their supportive function. IL‐6, abundantly secreted by TAMs, serves as a critical bridge linking inflammatory signals to CSC stemness maintenance. By binding to IL‐6R on the CSC surface, IL‐6 activates the downstream JAK‐STAT3 signaling pathway; activated STAT3 translocates into the nucleus and directly initiates the expression of core pluripotency transcription factors such as Sox2, Nanog, and Oct4, thereby endowing tumor cells with stem cell characteristics [430]. Moreover, IL‐6‐STAT3 signaling upregulates the expression of antiapoptotic proteins, conferring upon CSCs resistance to chemotherapy and radiotherapy [431, 432]. TAM‐derived TGF‐β exerts a dual role in CSC regulation through both Smad‐dependent and noncanonical pathways [433, 434]. TGF‐β not only promotes tumor cells to complete EMT, which is itself a key route through which tumor cells acquire stem cell features, cells that undergo EMT simultaneously acquire mesenchymal invasive capacity and stem cell‐like self‐renewal ability. Concurrently, TGF‐β maintains the quiescent state and antiapoptotic capacity of established CSCs, enabling them to survive under adverse conditions and await the opportunity for reactivation [435]. WNT ligands secreted by TAMs bind to Frizzled receptors on the CSC surface, activating β‐catenin‐dependent canonical WNT signaling to sustain CSC self‐renewal and proliferative capacity [436, 437]. TAMs can similarly activate the Notch pathway in CSCs by expressing Notch ligands; Notch signaling plays an indispensable role in maintaining the undifferentiated state of CSCs and suppressing their terminal differentiation [417].

The interactions between TAMs and CSCs rely not only on long‐range transmission via soluble factors but also involve signals mediated by direct physical cell–cell contact. Notably, the Jagged‐Notch signaling axis serves as a key pathway in this context. TAMs express Jagged1 and Jagged2 ligands, which can directly bind to Notch receptors on CSCs. This interaction has been demonstrated to maintain the undifferentiated state of CSCs and suppress their terminal differentiation [438]. CD44 expressed by TAMs is the principal receptor for hyaluronan; CD44 signaling, jointly engaged by the hyaluronan‐rich matrix environment within the tumor, enhances the expression of stemness genes [439]. Integrin‐mediated TAM‐CSC adhesion also merits attention, these adhesive structures not only provide mechanical support but also directly regulate stemness gene expression through integrin signaling [440]. Osteopontin secreted by TAMs, through interaction with CD44, can also synergistically activate stemness‐maintaining pathways [441].

Research advances in recent years have further deepened our understanding of the mechanisms underlying TAM‐mediated CSC support. In hepatocellular carcinoma, the TREM2^+^ TAM subset was demonstrated to drive the enrichment and proliferation of CSCs by activating the PI3K/Akt/mTOR signaling pathway, suggesting TREM2 as a potential immunotherapeutic target directed against CSCs [442]. In NSCLC, elevated H3K18 lactylation transcriptionally activates YTHDF2, which promotes glycolysis and cancer stemness by recognizing m6A‐methylated SFRP2 [443]. This metabolism–epigenetics–CSC regulatory axis represents a novel mechanism linking lactate accumulation to stemness maintenance and therapy resistance.

TAMs promote tumor progression through four interconnected functional pillars: immunosuppression, angiogenesis, invasion/metastasis, and CSC support. The immunosuppressive environment protects CSCs from immune clearance, aberrant vasculature provides metabolic support for CSCs and opens metastatic conduits, and EMT activation simultaneously confers invasive capacity and stem cell features. Contrasting TAMs’ immunoparalysis in late‐stage sepsis with their active CSC support in tumors reveals how macrophage plasticity drives diametrically opposite functional extremes, functional collapse versus directional hijacking, highlighting shared regulatory nodes that may serve as universal therapeutic targets.

## 6 Cross‐Disease Perspectives: Lessons From Macrophage Dysregulation in Inflammation and Cancer

**FIGURE 6:**
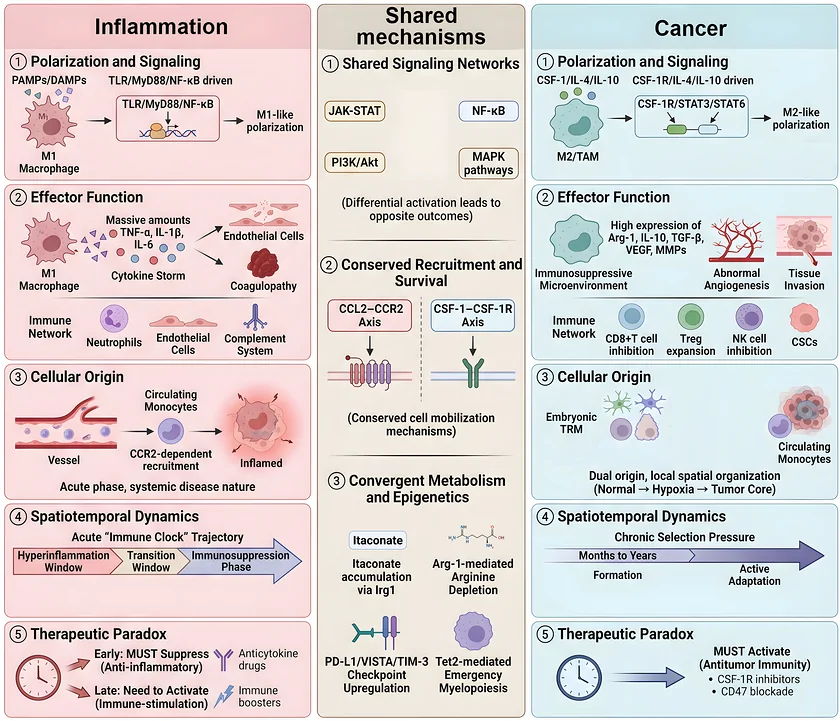
Cross‐disease perspectives on macrophage dysregulation in inflammation and cancer. This schematic illustrates the functional dichotomy and underlying mechanistic convergence of macrophages in acute sepsis and malignant tumors. The left column delineates the five defining features of macrophages during the hyperinflammatory phase of sepsis: M1‐like polarization via TLR/MyD88/NF‐κB signaling; effector functions characterized by massive release of TNF‐α, IL‐1β, and IL‐6, resulting in a cytokine storm and endothelial injury; an acute, systemic disease nature driven by CCR2‐dependent recruitment of circulating monocytes; a dynamic “immune clock” trajectory progressing from hyperinflammation through a transitional window to an immunosuppressive steady state; and a therapeutic paradox requiring early immunosuppression. Conversely, the right column illustrates the opposing features in the tumor microenvironment, where TAMs undergo M2‐like polarization via CSF‐1R/IL‐4/IL‐10‐driven STAT3/STAT6 signaling and SPI1/LILRB2‐mediated suppression of NF‐κB; they execute effector functions involving Arg‐1, IL‐10, TGF‐β, VEGF, and MMPs to establish an immunosuppressive microenvironment, aberrant angiogenesis, and tissue invasion; they exhibit a dual origin from both embryonic TRMs and circulating monocytes, characterized by local spatial organization; they follow a chronic, adaptive trajectory shaped by months to years of selection pressure; and they necessitate therapeutic activation of antitumor immunity. Bridging these opposing states, the middle column highlights three shared mechanistic pillars that govern macrophage plasticity: shared signaling networks (JAK–STAT, NF‐κB, PI3K/Akt, MAPK), conserved recruitment and survival mechanisms (CCL2–CCR2 and CSF‐1–CSF‐1R axes), and convergent metabolic and epigenetic dysfunctions, including itaconate accumulation, Arg‐1‐mediated arginine depletion, checkpoint upregulation (PD‐L1/VISTA/TIM‐3), and Tet2‐mediated emergency myelopoiesis. Collectively, these comparisons reveal that while macrophages are driven to opposite functional extremes across disease states, they rely on a common molecular toolkit, highlighting shared regulatory nodes as potential targets for cross‐disease therapeutic intervention. (Drafted by NanoBanana Pro 4K and subsequently manually redrawn and finalized by the author using BioRender.)

Macrophages, as the core effector cells of innate immunity, are highly plastic in function. This plasticity enables them to adapt to diverse environmental signals, yet in different pathological contexts, it can drive strikingly divergent disease processes. This “functional paradox” is exemplified by comparing lethal infectious diseases, a systemic inflammatory disorder, with cancer, typically a localized proliferative disease. In inflammation, macrophages often show an overactivated proinflammatory state, driving a fatal “cytokine storm”; in the tumor microenvironment, TAMs exhibit an immunosuppressive M2 like phenotype, promoting tumor growth and immune escape (Figure 6).

### 6.1 Core Differences: The Extreme Dichotomy of Functional Phenotypes

Macrophage biology in inflammation and malignant tumors reveals a near‐complete opposition in functional phenotypes. The activation trajectories, immunological roles, and pathological consequences elicited by these two pathological states are diametrically opposed. This section delineates these core differences across five dimensions.

#### 6.1.1 Polarization, Signaling Networks, and Functional Identity

In the hyperinflammation of sepsis and other types of inflammatory context, PAMPs and DAMPs drive macrophages into a proinflammatory state through PRRs. The TLR/MyD88/NF‐κB axis serves as a critical signaling conduit for this polarization [444]. Macrophages in this context function as immunological attackers, their defensive program fully mobilized against invading pathogens [445].

In malignant tumors, the signaling landscape diverges entirely. The tumor microenvironment maintains macrophage survival and proliferation through CSF‐1 [446], while IL‐4 and IL‐10 drive alternative activation, generating M2‐like TAMs that promote immune evasion, angiogenesis, and metastasis [447]. TGF‐β signaling simultaneously promotes an immunosuppressive phenotype and directly remodels the ECM [448]. Notably, inhibitory receptors regulated by transcription factors such as SPI1 in TAMs can suppress TLR‐mediated NF‐κB signaling, actively reinforcing the immunosuppressive state, a mechanism recently characterized in the context of macrophage tolerance, where SPI1 upregulates the inhibitory receptor LILRB2 to inhibit MyD88/NF‐κB signaling [449]. Furthermore, the CD47–SIRPα axis functions as a “don't eat me” checkpoint that specifically impairs phagocytosis of tumor cells [381]. Recent evidence demonstrates that CRISPR/Cas9‐mediated SIRPα knockout enhances macrophage phagocytosis of cancer cells, confirming the functional significance of this axis [450]. Rather than simple M2 polarization, TAMs, in a highly heterogeneous state, are integrated into a multilayered immunosuppressive network through functionally distinct signaling modules, becoming immunological accomplices hijacked to support tumor growth, angiogenesis, and immune escape.

#### 6.1.2 Effector Functions, Pathological Consequences, and Immune Cell Interactions

The effector outputs of macrophages in these two diseases or state are opposed in their molecular composition, tissue‐level consequences, and the immune cell networks they engage. The initial hyperinflammatory phase of sepsis and other context of inflammation is dominated by M1‐polarized macrophages that release large quantities of proinflammatory cytokines including TNF‐α, IL‐1β, and IL‐6. This cytokine storm triggers SIRS, endothelial injury, and coagulopathy, ultimately progressing to multiple organ dysfunction [451]. This represents collateral damage from excessive defense activation. The immune interactome of septic macrophages is organized around neutrophils, endothelial cells, and the complement system, which together constitute the core cellular circuitry of the hyperinflammatory response [452].

TAMs construct an entirely distinct pathological niche. Through high expression of Arg‐1, IL‐10, TGF‐β, VEGF, and MMPs, they establish a microenvironment characterized by profound immunosuppression, aberrant angiogenesis, and tissue invasion [386]. The immune interactome of TAMs is organized around a fundamentally different cellular network: M2‐like TAMs secrete IL‐10 and TGF‐β to inhibit cytotoxic T lymphocytes while expanding Treg cells and impairing NK cell function [453]. TAMs interplay with various cellular components within the tumor microenvironment to shape an immunosuppressive niche, consequently conferring immune evasion and immunotherapy tolerance [454]. More critically, TAMs engage in a bidirectional positive feedback loop with CSCs, forming a self‐reinforcing circuit that has no counterpart in acute infection [455].

#### 6.1.3 Cellular Origins and Spatial Organization

The cellular sources of macrophages driving pathology differ between inflammation and cancer. In inflammation, innate immune activation involves monocyte‐to‐macrophage fate bifurcation, representing a critical decision point in disease progression [456]. A large proportion of the macrophage‐like cells fueling the cytokine storm are monocyte‐derived infiltrates recruited acutely from the circulation [457]. The disease is inherently systemic, involving macrophage activation across multiple organs with marked heterogeneity.

In cancer, TAM origins are dual and complex. TAMs are derived from circulating monocytes recruited to tumor sites via chemotactic signals such as CCL2 and CSF‐1. TRMs, such as Kupffer cells in hepatocellular carcinoma, microglia in glioblastoma, and AMs in lung tumors, undergo local expansion and reprogramming. In parallel, circulating monocytes are continuously recruited and educated within the tumor microenvironment [458]. Moreover, TAM function exhibits characteristic spatial organization: M2‐like TAMs predominantly infiltrate hypoxic and stromal regions, where they secrete VEGF, TGF‐β, and MMPs to remodel the ECM [459]. This spatial ecosystem, organized around nutrient gradients and regional hypoxia, has no parallel in the systemic, time‐compressed macrophage activation of inflammation.

#### 6.1.4 Temporal Dynamics and Reversibility

The temporal architecture of macrophage dysregulation differs between the two diseases in timescale, mechanism, and reversibility. Typical inflammatory disease, sepsis, follows an acute trajectory now conceptualized as an “immune clock” comprising three overlapping phases: a hyperinflammatory window dominated by M1‐polarized macrophages, a transitional window marked by emerging M2 phenotypes and T‐regulatory expansion [460], and an immunosuppressive steady state characterized by sustained PD‐1 upregulation. This late‐phase state represents secondary functional collapse, dysfunction after depletion driven by exhaustion of inflammatory capacity [461]. Single‐cell multiomics analyses have identified irreversible immunosuppression beyond 72 h as a critical transition point, with the 0–18 and 36–48 h windows representing the most therapeutically tractable intervention periods [462]. In other inflammatory contexts, the M1‐to‐M2 polarization drift occurs spontaneously as part of an endogenous, albeit failed, wound‐healing program, in which macrophages transition toward an M2‐like state that, despite being dysfunctional in efferocytosis or resolution, remains responsive to pharmacological reprogramming [463].

TAM immunosuppression is established over months to years through chronic selective pressure within the tumor microenvironment [464]. Macrophages undergo gradual functional drift from initial antitumor potential toward progressive protumorigenic reprogramming, passing through intermediate states difficult to capture in cross‐sectional analyses [465, 466]. Unlike the acute, time‐compressed trajectory of inflammation, this represents active functional adaptation, a tamed phenotype acquired through sustained education. The reversibility of this state requires active therapeutic intervention to reprogram deeply entrenched regulatory circuits.

#### 6.1.5 The Therapeutic Paradox

The functional opposition detailed above generates a direct therapeutic paradox requiring opposite strategies. In the hyperinflammatory phase of sepsis and other inflammatory context, the clinical imperative is to suppress the excessive inflammatory response. Crucially, therapeutic timing is as critical as therapeutic target: early TNF‐α blockade dampens cytokine storm and improves survival, whereas the same intervention aggravates immune paralysis [462]. The identification of the SPI1/LILRB2 axis as a mechanism driving macrophage immunosuppressive phenotypes suggests that targeting this pathway holds promise for reversing immune paralysis in sepsis. The overarching principle is context‐dependent functional cooling, suggesting timing determines efficacy.

In cancer, the therapeutic goal is to reignite the antitumor immune functions quenched by the tumor microenvironment. TAM‐targeted strategies include CSF‐1R inhibitors to deplete or reprogram TAMs, CCL2 antagonists to block monocyte recruitment, and approaches to repolarize M2‐TAMs toward the M1 phenotype [467]. More recently, myeloid‐targeted immunocytokines have been developed that harness TREM2 antagonism and TAM‐protease‐activated IL‐2 to rewire myeloid‐lymphoid immunity, demonstrating high efficacy in preclinical tumor models by simultaneously reprogramming TAMs and activating T and NK cell compartments [468]. Disruption of the CD47–SIRPα “don't eat me” axis combined with chimeric antigen receptor (CAR) targeting further enhances macrophage phagocytic activity against solid tumors [469]. The principle is functional reheating through multiaxis immune activation.

The clinical implications of this opposition are stark. Anti‐inflammatory strategies that are lifesaving in inflammatory diseases (especially sepsis) may exacerbate immunosuppression and accelerate tumor progression if misapplied in cancer patients. Conversely, systemic macrophage activation, while therapeutically attractive in oncology, could precipitate rapid deterioration through amplified cytokine storm if misapplied in sepsis. This therapeutic paradox is the direct, clinically consequential expression of the extreme functional dichotomy that defines macrophage biology across these two disease categories.

The functional phenotypes of macrophages in hyperinflammation and malignant tumors are opposed across multiple dimensions: signaling networks, effector outputs, cellular origins, spatial organization, temporal dynamics, and therapeutic logic. These multidimensional oppositions generate a direct therapeutic paradox whereby strategies that prove lifesaving in one disease may be catastrophic in the other. Yet beneath this opposition, the two states converge on superficially similar immunosuppressive profiles despite their fundamentally different origins. This convergence reveals that their relationship is not one of simple binary opposition but of a complex continuum with shared regulatory nodes, which provide the conceptual foundation for cross‐disease therapeutic translation.

### 6.2 Underlying Similarities: The Common Foundation of Plasticity Mechanisms

While the functional phenotypes of macrophages in hyperinflammation and malignant tumors are opposed, these polarities are achieved through a shared set of core mechanistic tools. This convergence is rooted in the essential nature of macrophages as highly plastic cells that integrate diverse microenvironmental signals to dynamically adjust their functional states. This section dissects the deep mechanistic commonalities across three dimensions: shared signaling networks, conserved recruitment and survival mechanisms, and convergent metabolic and epigenetic reprogramming.

#### 6.2.1 Shared Signaling Networks Governing Plasticity

The core signaling networks that control macrophage functional transitions are conserved across both diseases. The JAK–STAT, NF‐κB, PI3K/Akt, and MAPK pathways constitute a shared molecular framework that drives macrophage polarization in both inflammation and cancer.

The functional output depends entirely on differential pathway activation. In early‐phase sepsis and the other contexts of inflammation, JAK2/STAT1 signaling is a critical driver of M1‐like proinflammatory polarization [470, 471]. In the tumor microenvironment, the JAK–STAT pathway is not silenced but redirected, with STAT3 and STAT6 activated by tumor‐derived IL‐4, IL‐10, and CSF‐1 to drive an immunosuppressive TAM phenotype [472]. Similarly, the NF‐κB pathway acts as a central regulatory hub. In inflammatory contexts, NF‐κB drives the transcription of proinflammatory cytokines like TNF‐α and IL‐1β. In tumors, NF‐κB signaling is actively hijacked [473]. Tumor cells induce IRG1 expression in TAMs via the NF‐κB pathway, leading to itaconate accumulation and subsequent immune suppression [474]. This demonstrates not only a shared pathway but also the precise point of divergence leading to opposing functional outcomes. In sepsis and atherosclerosis, the PI3K‐AKT pathway is activated during the immunosuppressive phase and predominantly drives M2‐like polarization, whereas its inhibition has been shown to restore bacterial clearance and improve survival [475]. In tumors, CSF‐1R‐associated PI3K–AKT signaling underpins M2‐like TAM survival and maintenance, representing a therapeutic vulnerability [476]. In both early‐phase sepsis and cancer, the parallel activation of p38 and ERK has been shown to orchestrate key pathological features. In transitional or late‐phase sepsis, combined inhibition of these kinases reduces inflammatory cytokine production in macrophages [477], while in tumors, p38/ERK signaling can be co‐opted to induce immunosuppressive programs, thereby demonstrating a shared functional collaboration in distinct immune contexts [478]. The fundamental logic is conserved: the same signal transduction networks are recruited in both diseases, with functional outcomes dictated by the specific combinations of upstream signals.

#### 6.2.2 Conserved Recruitment and Survival Mechanisms

The molecular mechanisms that mobilize monocytes to pathological sites are remarkably similar. The CCL2–CCR2 chemokine axis serves as the central and most conserved pathway for monocyte recruitment in both conditions. In sepsis, TRMs sense PAMPs and rapidly secrete CCL2 to recruit peripheral blood monocytes to sites of infection [479]. In tumors, cancer cells and stromal cells continuously express CCL2, a process that sustains the recruitment of monocytes into the tumor microenvironment to replenish the TAM pool. High CCL2 levels are associated with more aggressive malignancies and poorer outcomes, highlighting its pathological significance [480].

Beyond CCL2, the CSF‐1–CSF‐1R axis plays an indispensable role across both pathological settings. In the tumor microenvironment, this axis is well established as the core pathway for TAM survival and M2‐like polarization [446]. In sepsis, the CSF‐1R is likewise essential for monocyte‐to‐macrophage differentiation and survival, consistent with its canonical role in macrophage homeostasis [481]. This substantial overlap in chemokine and growth factor signaling implies that targeting these mechanisms may have cross‐domain therapeutic potential, though therapeutic windows differ.

#### 6.2.3 Convergent Metabolic and Epigenetic Dysfunction

Under chronic or severe stimulation, macrophages in both diseases converge on a state of functional exhaustion characterized by shared metabolic and epigenetic features. A hallmark of this convergence is the accumulation of the metabolite itaconate. In late‐phase sepsis, itaconate serves as an anti‐inflammatory metabolite that inhibits succinate dehydrogenase and NLRP3 inflammasome activation [482]. In the tumor microenvironment, TAMs also accumulate itaconate via IRG1, which is induced by NF‐κB signaling [474]. However, in this context, itaconate acts to suppress T‐cell chemoattractants like CXCL9 and CXCL10, directly impairing antitumor immunity [483]. This demonstrates that a single shared metabolite can serve as a functional bridge between the two diseases, though its pathological consequence is context dependent. Arginine metabolism represents another core node. In septic immunoparalysis and within tumors, high Arg‐1 expression depletes l‐arginine, impairing T cell proliferation and effector function [294]. This shared mechanism directly links macrophage‐driven metabolic starvation to adaptive immune suppression in both diseases.

At the immunological level, this dysfunction converges on shared checkpoint expression. PD‐L1 is highly expressed on macrophages in both late sepsis and the tumor microenvironment, where it suppresses T cell function and drives exhaustion. Other checkpoints like VISTA, TIM‐3, and LAG‐3 are also upregulated, forming a conserved molecular network for immune evasion [484, 485].

At the systemic level, both disease states are characterized by a common maladaptive process: emergency myelopoiesis. Both sepsis and cancer can reprogram the bone marrow to continuously produce functionally compromised myeloid cells at the expense of lymphopoiesis, a state termed tumor‐driven myeloid bias in cancer and endotoxin tolerance‐driven reprogramming in sepsis [486, 487, 488]. Emerging evidence suggests a conserved epigenetic mechanism. Tet2, a known regulator of myelopoiesis, was shown to promote infection‐induced emergency myelopoiesis by oxidizing mRNA [489], revealing a posttranscriptional regulatory layer that may also be operative in tumor‐driven myeloid bias.

This convergence of metabolic profiles, checkpoint molecule expression, and systemic hematopoietic reprogramming reveals that even when macrophages are driven to opposite functional extremes, they rely on a common molecular toolkit, establishing TAMs in tumors and functionally exhausted macrophages in sepsis as fundamentally linked states.

## 7 Targeting Macrophages: Promising Keys to Unlock Inflammation and Cancer Treatment in the Future

Building upon the functional duality of macrophages and their shared regulatory nodes in inflammation and cancer, targeting macrophages has evolved from simple activation or inhibition toward a strategy of “recalibration” based on precise disease staging and molecular phenotyping. In inflammation, the therapeutic time window dictates strategy: the cytokine storm phase requires suppression of excessive macrophage activation, whereas the immunoparalysis phase demands reversal of functional collapse. In cancer, the goal is to dismantle the immunosuppressive TAM niche by reprogramming TAMs into antitumor effector cells or blocking their protumor functions. This section overviews macrophage‐targeted therapies across three levels: clinically validated strategies, frontier approaches, and key translational studies, with an emphasis on bidirectional borrowing between inflammation and cancer.

### 7.1 Strategies

Current macrophage‐targeting therapeutic strategies have progressively moved from laboratory concepts toward clinical validation, with some agents already approved for marketing and many more undergoing evaluation at various stages of clinical trials. These strategies are organized around the key nodes of the macrophage life cycle, recruitment, polarization, survival, phagocytosis, and immune checkpoints, constituting a multilayered intervention system. It is noteworthy that the therapeutic strategy for the same target in inflammation and in cancer may be diametrically opposed or entirely convergent; this complexity is a direct reflection of macrophage plasticity and demands that disease context and disease phase be fully considered when designing therapeutic strategies. Tables 2 and 3 have summarized the preclinical studies and clinical trials of macrophage‐targeting therapies respectively.

**TABLE 2:** Preclinical and animal studies of macrophage‐targeted therapies in inflammation and cancer.

Therapeutic strategy	Disease	Agent/approach	Key Findings	References
Blocking recruitment	Sepsis	CCR2 inhibitor (RS504393), CCR2 deficiency	Reduces monocyte infiltration, attenuates inflammatory injury, improves survival in mouse sepsis models	[490]
		CRV peptide‐modified lipid nanoparticles (in situ CAR‐M programming)	Enables pathogen‐specific targeting of MRSA, avoids systemic immunosuppression	[491]
	Atherosclerosis	CCR2 inhibitor (Apoe^−^/^−^ mice)	Promoted atherosclerosis regression and enriched M2‐like macrophages within plaques	[492]
		Self‐assembled peptide‐conjugated nanoparticle (CCTV)	Inhibited monocyte chemotactic migration and attenuated NLRP3 inflammasome activation	[493]
Reprogramming polarization	Sepsis	NLRP3 inhibitor (MCC950)	Attenuates cytokine storm and organ injury in multiple animal models	[494, 495]
		PI3K inhibitor (IPI549)	Reverses M2b polarization, restores bactericidal function	[475]
		Icariin	Increases M1/M2 ratio, enhances bactericidal function via RSK2–YAP–cGAS–IFN‐β pathway	[496]
		Modified Xi‐Jiao‐Di‐Huang decoction	Promotes M1‐to‐M2 transition by inhibiting PIM2/NF‐κB pathway	[497]
		Ombuin (AI‐screened)	Suppresses NF‐κB‐driven M1 polarization as an ALDH2 agonist	[498]
		Self‐assembled metabolic regulator (targeting HK2 and STING)	Induces M1‐to‐M2 polarization via CCL2 signaling	[499]
		PFKFB3 inhibitors	Restores macrophage bactericidal capacity, relieves glycolysis‐induced phagocytic impairment, improves survival	[500]
		Itaconate derivatives	Protective anti‐inflammatory effects in septic cytokine storm phase	[499]
	Rheumatoid arthritis	Nanoparticle‐delivered IL‐4	Reprograms synovial M1 macrophages to an M2 phenotype in situ, suppressing synovitis and bone destruction	[501]
Reprogramming polarization	Rheumatoid arthritis	Platelet‐derived exosomes (PLT‐Exos)	Stimulates M1‐to‐M2 macrophage conversion in situ, significantly reducing synovial inflammation, bone erosion, and cartilage damage in collagen‐induced arthritis mice	[502]
		Engineered Panax notoginseng polysaccharide micelles (FA‐PPI‐Ms)	Promotes M2 macrophage polarization by inhibiting the JAK2/STAT3 signaling pathway, restoring M1/M2 balance and alleviating joint swelling and inflammation in a rat model of RA	[503]
	Inflammatory bowel disease	Chondroitin sulfate‐functionalized curcumin nanoparticles (OCC NPs)	Reprograms intestinal macrophages toward an anti‐inflammatory phenotype by inhibiting the TLR4/MyD88/NF‐κB pathway, attenuating mucosal injury	[504]
		Human umbilical cord MSC‐derived exosomes (hucMSC‐Ex)	Promotes M2 macrophage polarization via the METTL3–Slc37a2–YTHDF1 axis, alleviating intestinal inflammation in colitis models	[505]
		Endometrial regenerative cell‐derived exosomes (ERC‐Exos)	Promotes M2 macrophage polarization via SIRT6‐mediated NF‐κB blockade, significantly alleviating ulcerative colitis in mice	[506]
	Atherosclerosis	Metformin	Promotes M1‐to‐M2 macrophage polarization through hematopoietic AMPK–ATF1 and AMPK–STAT3 signaling, attenuating plaque progression in preclinical models	[507]
	Neuroinflammatory diseases	CSF‐1R inhibitors (PLX5622)	Depletes proinflammatory microglia; repopulated cells adopt an M2‐biased phenotype and reduce neuroinflammation	[508]
		Intranasal IL‐4/PPARγ agonists	Reprograms microglia toward a neuroprotective M2 phenotype via STAT6/PPARγ signaling, promoting functional recovery in preclinical models	[509]
	Cancer	Oral plant‐derived LNPs delivering CRISPR‐Cas9 targeting Lsd1	∼60% editing efficiency in macrophages, promotes H3K4 methylation, therapeutic in colitis and colitis‐associated tumorigenesis	[510, 511]
	Cancer (colorectal)	Nanosheet immunoswitches (PA‐WSe_2_, LA‐WS_2_)	Bidirectionally polarizes macrophages (M1 in tumor, M2 in sepsis)	[512]
	Cancer (multiple solid tumor models)	Glutaminase inhibitors	Blocks M2‐like TAM polarization, restores M1‐like antitumor phenotype	[513, 514]
Enhancing phagocytosis	Sepsis	Probiotic‐derived EVs	Increases bacterial uptake via FPR1/2 signaling	[515]
		Oroxylin A	Induces trained immunity, increases glycolysis and mTOR phosphorylation, promotes LC3‐associated phagocytosis	[516]
		Fibroblast growth factor 8 (FGF8)	Enhances bacterial phagocytosis and killing via ERK1/2, reduces mortality	[517]
	Atherosclerosis	Resolvin D1	Enhances macrophage efferocytosis and promotes plaque stability in Ldlr^−/−^ mice	[518]
		Macrophage membrane biomimetic nanoparticles (MM@Lips‐SHP1i)	Competitively blocks CD47–SIRPα signaling, enhancing efferocytosis and attenuating plaque inflammation in Apoe^−/−^ mice	[519]
	Inflammatory bowel disease	Oxidation‐responsive nanoparticles containing annexin A1‐mimetic peptide Ac2‐26 (AON)	Promotes macrophage efferocytosis of apoptotic neutrophils, reducing colonic inflammation and accelerating mucosal healing in DSS‐induced colitis mice	[520]
	Multiple sclerosis	TREM2 agonistic antibody	Promotes microglial clearance of myelin debris, enhances remyelination, and preserves axonal integrity in the cuprizone model of CNS demyelination	[521]
	Cancer (multiple solid tumor models)	Macrophage‐derived engineered EVs	Natural macrophage targeting; can be loaded with miRNAs, siRNAs, or small molecules	[522]
Inhibiting immunosuppressive signals	Sepsis	Anti‐PD‐1 antibody (late phase)	Improves survival, restores T cell IFN‐γ, enhances macrophage phagocytosis in CLP model	[523]
		Anti‐PD‐L1 antibody	Restores immune effector function, improves survival in fungal sepsis models	[524]
		Oral plant‐derived LNPs delivering CRISPR–Cas9	Enables macrophage‐specific gene editing, programmable regulation of immunosuppressive genes	[510]
	Cancer (multiple tumor models)	TGF‐β blocking antibodies and receptor kinase inhibitors (e.g., galunisertib)	Enhances antitumor immunity, reduces metastasis in preclinical models	[525]
Inhibiting immunosuppressive signals	Cancer (hepatocellular carcinoma)	COX‐2 inhibitors	Shows promise in preclinical hepatocellular carcinoma models	[526]

**TABLE 3:** Clinical studies of macrophage‐targeted therapies in inflammation and cancer.

Therapeutic strategy	Disease	Agent/approach	Phase (NCT No.)	Key results	References
Blocking recruitment	Cancer (tenosynovial giant cell tumor)	Pexidartinib (CSF‐1R inhibitor)	FDA approved (Phase III, NCT02371369)	39% ORR vs. 0% placebo; first approved TAM‐targeted agent	[527]
	Cancer (glioblastoma)	PLX3397 (CSF‐1R inhibitor)	Phase II (NCT01349036)	Failed to improve survival (PFS^a^ 3.2 vs. 2.9 mo)	[528, 529]
	Cancer (pancreatic)	PF‐04136309 (CCR2 inhibitor) + FOLFIRINOX	Phase Ib (NCT01413022)	Superior ORR^b^ and immune reprogramming vs. chemotherapy alone	[530]
	Cancer (diffuse‐type tenosynovial giant cell tumor)	Emactuzumab (anti‐CSF‐1R mAb)	Phase III (NCT05417789)	Comparable efficacy to pexidartinib; being evaluated with PD‐L1 inhibitors	[531]
	Cancer (pancreatic)	Cabiralizumab (anti‐CSF‐1R mAb) + nivolumab	Phase II (NCT04050462)	No significant PFS improvement; enhanced responses in subgroups	[532]
	Cancer (HER2+ solid tumors)	HER2‐targeting CAR‐M (CT‐0508)	Phase I (NCT04660929)	Safe, no dose‐limiting toxicities	[533]
	Rheumatoid arthritis	Anti‐CCR2 monoclonal antibody MLN1202	Phase IIa (NCT01015560)	Effective target engagement (57–94% reduction of free CCR2 on CD14^+^ monocytes) but no reduction in synovial inflammation or clinical improvement	[534]
Reprogramming polarization	Sepsis (MALS)	Anakinra (IL‐1 receptor antagonist)	Phase III (ImmunoSep^c^, NCT04990232)	SOFA^d^ improvement in MALS^e^ patients (ferritin >4420 ng/mL)	[535]
	Sepsis (immunoparalysis)	Recombinant human IFN‐γ	Phase III (ImmunoSep, NCT04990232)	Restores immune function in immunoparalysis (mHLA‐DR <5000 AB/cell)	[535]
		IFN‐γ	Phase II (INFINITY, NCT06694740)	Ongoing; for mHLA‐DR‐defined immunosuppression	[535]
Reprogramming polarization	Inflammatory bowel diseases	hucMSCs‐Exos^f^	Early Phase I (NCT06853522)	Ongoing	NCT06853522
	Cancer (pancreatic)	CD40 agonists	Phase Ib/II (NCT04888312)	Converts immunologically “cold” tumors to “hot” tumors	[536]
Enhancing phagocytosis	Cancer (B‐cell non‐Hodgkin lymphoma)	Magrolimab (anti‐CD47) + rituximab	Phase Ib/II (NCT02953509)	ORR 52.2%, CR^g^ 30.4%	[537]
	Cancer (myelodysplastic syndrome)	Magrolimab + azacitidine	Phase III (NCT04313881)	Terminated; increased death risk	[538]
	Cancer (B‐cell non‐Hodgkin lymphoma)	Evorpacept (SIRPα–Fc) + lenalidomide + rituximab	Phase I (NCT05025800)	Safe, 80% CR, 2‐year PFS 69%	[539]
Inhibiting immunosuppressive signals	Sepsis	Anti‐PD‐L1 antibody (BMS‐936559)	Phase Ib (NCT02576457)	Restores monocyte HLA‐DR, no cytokine storm	[540]
	Cancer (advanced solid tumors)	Anti‐VISTA antibody (HMBD‐002)	Phase II (NCT05082610)	Reprograms TAMs toward inflammatory phenotype	[541, 542]

^a^ Progression‐free survival.

^b^ Objective response rate.

^c^ Personalized immunotherapy in sepsis (name of the trial).

^d^ Sequential organ failure assessment.

^e^ Macrophage activation‐like syndrome.

^f^ Human umbilical cord mesenchymal stem cells‐derived exosomes.

^g^ Complete response.

#### 7.1.1 Blocking Macrophage Recruitment

Blocking macrophage recruitment represents one of the most direct strategies for reducing pathological macrophage tissue infiltration. In sepsis, small‐molecule CCR2 inhibitors such as RS504393 and CCR2‐deficiency have shown efficacy in reducing monocyte tissue infiltration, attenuating inflammatory injury, and improving survival in multiple mouse sepsis models [490]. However, the time window for CCR2 blockade is critically important: blockade during the early phase of infection may impair pathogen clearance and paradoxically accelerate death, whereas intervention during the cytokine storm phase and the transition toward immunoparalysis may confer benefit. Accordingly, partial agonism or selective inhibition of downstream isoforms such as PI3Kγ may represent safer and more efficacious approaches than complete blockade [543]. Beyond small molecule inhibitors, novel delivery platforms are being developed to enhance targeting precision. CRV peptide‐modified lipid nanoparticles have been developed for in situ programming of CAR‐M targeting MRSA, demonstrating the potential of nanoparticles enabled in vivo macrophage engineering for infectious diseases [491]. This approach achieves pathogen‐specific targeting while avoiding systemic immunosuppression.

In RA, where CCR2 is abundantly expressed on synovial monocytes and macrophages, clinical translation of CCR2 blockade has proven challenging. A Phase IIa trial of the anti‐CCR2 monoclonal antibody MLN1202 in active RA patients demonstrated effective target engagement, reducing free CCR2 on CD14^+^ monocytes by 57–94%, but failed to show any reduction in synovial inflammation or clinical improvement [534]. These negative results suggest that redundancy in chemokine signaling or compensatory recruitment pathways may limit the efficacy of single‐target blockade in autoimmune arthritis.

In atherosclerosis, CCL2/CCR2 axis plays a critical role in monocyte recruitment to atherosclerotic plaques, CCR2 inhibition has demonstrated therapeutic potential. Treatment with a CCR2 inhibitor in Apoe^−/−^ mice promoted atherosclerosis regression and was associated with enrichment of M2‐like macrophages within plaques, indicating that blocking monocyte recruitment may facilitate a switch toward a more reparative macrophage phenotype [492]. To enhance targeting precision, nanoparticle‐based CCR2 antagonists have been developed. A self‐assembled peptide‐conjugated nanoparticle (CCTV) exhibited CCR2 antagonistic properties, inhibited chemotactic migration of primary monocytes, and attenuated inflammasome activation via NLRP3 downregulation in atherosclerotic models [493].

In cancer, the CSF‐1R inhibitor pexidartinib received FDA approval in 2019 for tenosynovial giant cell tumor (TGCT) based on the Phase III ENLIVEN trial, which demonstrated a 39% overall response rate compared with 0% in the placebo arm, making it the first approved TAM‐targeted agent [527]. In January 2025, pexidartinib also entered the Chinese market, having been granted priority review by the NMPA. However, in solid tumors such as glioblastoma, single‐agent CSF‐1R inhibition with PLX3397 failed to improve survival in a Phase II trial, with median progression‐free survival of only 3.2 versus 2.9 months in controls [528], as tumors developed resistance through compensatory PI3K–AKT activation and increased Treg infiltration [529]. Accordingly, strategies combining CSF‐1R inhibitors with radiotherapy, chemotherapy, or immune checkpoint inhibitors are currently under exploration in multiple clinical trials [544, 545, 546]. In the oncological arena, the CCR2 inhibitor PF‐04136309 combined with the FOLFIRINOX chemotherapy regimen demonstrated superior local immune reprogramming and objective response rates compared with chemotherapy alone in a Phase Ib clinical trial in pancreatic cancer [530]. In addition to pexidartinib, other CSF‐1R targeting agents have been evaluated clinically. Emactuzumab, a monoclonal antibody targeting CSF‐1R, has shown comparable efficacy to pexidartinib in diffuse type TGCT, and is currently under clinical investigation in combination with PD‐L1 inhibitors in multiple solid tumors [531]. Cabiralizumab, another anti‐CSF‐1R monoclonal antibody, when combined with nivolumab in a Phase II trial in pancreatic cancer, did not significantly improve progression free survival, yet enhanced antitumor immune responses were observed in specific subgroups [532], suggesting that the benefit of CSF‐1R inhibition may require biomarker based precision patient selection. In parallel, CAR‐M have emerged as a novel platform for precise macrophage targeting. A Phase I trial of HER2 targeting CAR‐M (CT 0508) has demonstrated a safe profile with no dose limiting toxicities [533], and systematic evaluation of CAR formats identified that CAR‐M with CD3ζ TLR4 intracellular domains elicit robust adaptive immune activation and synergize with PD‐1/PD‐L1 therapy [547].

#### 7.1.2 Reprogramming Macrophage Polarization

Reprogramming the polarization state of macrophages is a strategy that fundamentally reverses their pathological functions and carries diametrically opposite therapeutic goals in sepsis and in cancer. In sepsis, the goal during the hyperinflammatory phase is to suppress excessively activated M1‐like macrophages to mitigate inflammatory injury. The NLRP3 inflammasome inhibitor MCC950 has effectively attenuated cytokine storms and organ injury in multiple animal models [494, 495]. More importantly, the landmark ImmunoSep trial provided the strongest clinical evidence for phenotype‐guided macrophage targeting: patients with macrophage activation‐like syndrome (MALS; ferritin >4420 ng/mL) treated with the IL‐1 receptor antagonist anakinra showed significant improvement in SOFA scores compared with placebo [535]. Beyond this clinical breakthrough, multiple preclinical studies have identified promising agents that modulate macrophage polarization to reverse sepsis‐induced immunosuppression. Icariin increases the M1/M2 ratio and enhances bactericidal function via the RSK2–YAP–cGAS–IFN‐β pathway [496]. The modified Xi‐Jiao‐Di‐Huang decoction promotes M1‐to‐M2 transition by inhibiting PIM2/NF‐κB [497]. AI‐screened ombuin suppresses NF‐κB‐driven M1 polarization as an ALDH2 agonist [498]. A self‐assembled metabolic regulator targeting HK2 and STING induces M1‐to‐M2 polarization via CCL2 signaling [499]. PI3K inhibitor IPI549 reverses M2b polarization and restores bactericidal function [475]. Beyond these agents, PFKFB3 inhibitors have shown promise in animal models of septic immunoparalysis, restoring macrophage bactericidal capacity by relieving glycolysis‐induced phagocytic impairment and significantly improving survival [500]. Itaconate derivatives have demonstrated protective anti‐inflammatory effects in the septic cytokine storm phase [499], though their effects in tumors vary by tumor type. Collectively, these studies demonstrate that reprogramming macrophage polarization, whether by suppressing excessive M1 activation during cytokine storm or promoting M2 polarization to reverse immunoparalysis, represents a viable therapeutic strategy for sepsis

In chronic and autoimmune inflammatory diseases, the therapeutic goal shifts to suppressing persistent M1‐driven inflammation or inducing a transition toward M2‐like anti‐inflammatory and tissue‐reparative phenotypes. In RA, nanoparticle‐delivered IL‐4 reprograms synovial M1 macrophages to an M2 phenotype in situ, suppressing synovitis and bone destruction [501]. Platelet‐derived exosomes (PLT‐Exos) effectively stimulate M1‐to‐M2 macrophage conversion in situ, significantly reducing synovial inflammation, bone erosion, and cartilage damage in collagen‐induced arthritis mice [502]. Engineered Panax notoginseng polysaccharide micelles (FA‐PPI‐Ms) promote M2 macrophage polarization by inhibiting the JAK2/STAT3 signaling pathway, restoring M1/M2 balance and markedly alleviating joint swelling and inflammation in a rat model of RA [503].

In inflammatory bowel disease, orally delivered curcumin‐loaded nanoparticles, such as chondroitin sulfate‐functionalized OCC NPs, reprogram intestinal macrophages toward an anti‐inflammatory phenotype by inhibiting the TLR4/MyD88/NF‐κB pathway, thereby attenuating mucosal injury [504]. Similarly, exosomes derived from human umbilical cord mesenchymal stem cells (hucMSC‐Ex) or endometrial regenerative cells (ERC‐Exos) promote M2 macrophage polarization via the METTL3–Slc37a2–YTHDF1 axis or SIRT6‐mediated NF‐κB blockade, respectively, significantly alleviating intestinal inflammation in colitis models [505, 506]. An Early Phase 1 clinical trial (NCT06853522) evaluating the safety and efficacy of exosomes derived from human umbilical cord mesenchymal stem cells (hUC‐MSCs‐Exos) in patients with moderate‐to‐severe active ulcerative colitis is currently in the recruiting phase, but no results have been reported yet.

In atherosclerosis, metformin promotes M1‐to‐M2 macrophage polarization through hematopoietic AMPK–ATF1 and AMPK–STAT3 signaling, thereby attenuating plaque progression in preclinical models [507]. In neuroinflammatory diseases, CSF‐1R inhibitor‐mediated depletion and repopulation yields M2‐biased microglia and reduces neuroinflammation [508]. Additionally, intranasal IL‐4 or PPARγ agonists reprogram microglia toward a neuroprotective M2 phenotype, promoting functional recovery in preclinical models [509].

In cancer, the goal is to “reignite” M2‐like TAMs toward an M1‐like antitumor phenotype. CD40 agonists have shown potential to convert immunologically “cold” pancreatic tumors into “hot” ones [536]. TLR agonists (imiquimod, poly(I:C), CpG) and STING agonists activate PRRs and the cGAS–STING pathway, bridging innate and adaptive antitumor immunity [548, 549]. Of note, a recent study developed functional nanosheet immunoswitches that can bidirectionally polarize macrophages based on disease context, offering a unified platform for both cancer and sepsis immunotherapy [512]. Engineered EVs represent another precision tool for macrophage reprogramming. Anti‐CD80 MTX engineered EVs targeting inflammatory CD80‐positive macrophages delivered methotrexate intracellularly, reduced CD80‐positive macrophage populations, promoted Treg differentiation, and suppressed Th1 cell production in cancer and inflammatory disease models [550]. Besides, glutaminase inhibitors have effectively blocked M2 like polarization of TAMs in multiple solid tumor models, enabling TAMs to reacquire an M1 like antitumor phenotype [513, 514]. Epigenetic editing offers an additional layer of precision for macrophage polarization control. Oral plant‐derived lipid nanoparticles for CRISPR–Cas9 ribonucleoprotein delivery targeting Lsd1 in macrophages achieved nearly 60% editing efficiency, promoted H3K4 methylation, and demonstrated therapeutic effects in colitis and colitis‐associated tumorigenesis [510]. Lsd1 knockout in macrophages promoted anti‐inflammatory features including downregulated TNFα and upregulated IL10 [511], providing a proof of concept for epigenetic reprogramming of macrophage polarization states in inflammatory diseases and cancer.

#### 7.1.3 Enhancing Macrophage Phagocytosis

Enhancing the phagocytic function of macrophages is a shared goal that transcends the therapeutic boundaries of inflammation and cancer, yet the therapeutic logic differs between the two disease contexts. In sepsis, enhancing phagocytosis focuses on restoring the intrinsic phagocytic and bactericidal functions that become impaired during immunoparalysis. Recent studies have identified various approaches to augment phagocytic activity in septic patients. Probiotic‐derived EVs increase macrophage bacterial uptake through activation of the FPR1/2 signaling pathway [515]. Oroxylin A induces trained immunity in macrophages by increasing glycolysis and mTOR phosphorylation, promoting LAP [516]. Fibroblast growth factor 8 enhances bacterial phagocytosis and killing through ERK1/2 signaling, with recombinant FGF8 administration reducing mortality in septic mice [517].

In atherosclerosis, impaired efferocytosis by plaque macrophages drives secondary necrosis and plaque destabilization. Resolvin D1 enhances macrophage efferocytosis and promotes plaque stability in Ldlr^−/−^ mice [518]. Macrophage membrane biomimetic nanoparticles (MM@Lips‐SHP1i) competitively block CD47–SIRPα signaling, enhancing efferocytosis and attenuating plaque inflammation in ApoE^−/−^ mice [519]. In inflammatory bowel disease, defective clearance of apoptotic cells by intestinal macrophages perpetuates mucosal injury. Mesenchymal stem cell‐derived exosomes, such as HucMSCs‐Exos, promote M2 macrophage polarization via delivery of miR‐21‐5p targeting the SPRY2/ERK axis, thereby reducing colitis severity in TNBS‐ and DSS‐induced mouse models [551]. Oxidation‐responsive nanoparticles containing the annexin A1‐mimetic peptide Ac2‐26 (AON) promote macrophage efferocytosis of apoptotic neutrophils and induce M1‐to‐M2 phenotypic switching, thereby reducing colonic inflammation and accelerating mucosal healing in DSS‐induced colitis mice [520]. In multiple sclerosis, TREM2 agonistic antibody promotes microglial clearance of myelin debris, enhances remyelination, and preserves axonal integrity in the cuprizone model of CNS demyelination [521]. While numerous preclinical studies across these diseases have identified promising targets to enhance macrophage phagocytosis and efferocytosis, clinical evidence remains sparse.

In cancer, the core strategy is blocking “don't eat me” signals on tumor cells. The CD47–SIRPα axis has emerged as a promising target in cancer immunotherapy. However, clinical translation has faced significant challenges. The anti‐CD47 antibody magrolimab initially showed encouraging efficacy in combination with rituximab for non‐Hodgkin lymphoma. In a Phase 1b/2 trial of 46 patients with relapsed/refractory indolent NHL, including rituximab‐refractory patients, the overall response rate was 52.2% with a complete response rate of 30.4% [537]. Nevertheless, the Phase III ENHANCE trial in myelodysplastic syndrome was terminated due to failure to meet its primary endpoint, ultimately leading to discontinuation of the magrolimab development program [538]. This setback was primarily attributed to “on‐target off‐tumor” hematological toxicity, particularly anemia. Consequently, therapeutic strategies have evolved toward next‐generation approaches including SIRPα–Fc fusion proteins, bispecific antibodies, and CAR‐M. The novel CD47 blocker evorpacept (ALX148), which avoids erythrocyte binding, has demonstrated a safe toxicity profile in a Phase I trial combined with lenalidomide and rituximab for B‐cell non‐Hodgkin lymphoma, with 80% of patients achieving complete response and a 2‐year progression‐free survival rate of 69% [539]. The core clinical lesson from the CD47–SIRPα axis is that merely releasing the “don't eat me” signal may be insufficient for adequate antitumor immunity; a concomitant “eat me” signal, such as Fc‐mediated phagocytic signals provided by antitumor antibodies or immunogenic cell death signals released by chemotherapy and radiotherapy, must be provided synchronously for effective macrophage‐mediated tumor clearance [552]. To further enhance phagocytic precision, nanoparticle‐based delivery systems are being developed. Macrophage‐derived engineered EVs, by virtue of carrying surface proteins from parental cells, exhibit natural macrophage targeting properties and can be loaded with specific miRNAs, siRNAs, or small molecule drugs to enhance phagocytic function in a cell type‐specific manner [522].

#### 7.1.4 Inhibiting Immunosuppressive Signals

Blocking immunosuppressive signals is a strategy that directly relieves the suppression imposed upon downstream effector cells by macrophages. In sepsis, macrophages in the late immunosuppressive phase contribute to immune dysfunction through multiple pathways. PD‐L1 expression is significantly upregulated on monocytes/macrophages during sepsis, driving M1 polarization via the AIM2/IL‐18/STAT1 signaling axis, with upregulation mediated by the PI3K/Akt and p38 MAPK pathways upon LPS stimulation [553]. Targeting the PD‐1/PD‐L1 axis has been evaluated in both clinical and preclinical settings; a Phase 1b study of the anti‐PD‐L1 antibody BMS‐936559 in 24 septic patients restored monocyte HLA‐DR expression without drug‐induced cytokine storm [540], and preclinical models have shown that anti‐PD‐L1 therapy improves survival by restoring macrophage phagocytic activity and T‐cell IFN‐γ production [554]. In animal models, anti‐PD‐1 antibody administered during the late phase of sepsis significantly improved survival, restored T cell IFN‐γ production, and enhanced macrophage phagocytic function in the cecal ligation and puncture mouse model [523]. In fungal sepsis models, anti‐PD‐L1 treatment likewise restored immune effector function and improved survival [524]. The core challenge remains that administration during the cytokine storm phase could accelerate death, whereas administration during the immunoparalysis phase may confer benefit. Beyond PD‐1/PD‐L1, the IL‐1 pathway has been clinically validated; the ImmunoSep trial demonstrated that the IL‐1 receptor antagonist anakinra significantly improved SOFA scores in macrophage activation‐like syndrome patients [535], and the ongoing INFINITY trial (NCT06694740) is investigating IFN‐γ in mHLA‐DR‐defined immunosuppression. Other immunosuppressive pathways involve TGF‐β and IL‐10. Emerging epigenetic strategies offer new avenues for blocking immunosuppressive signals at the transcriptional level. Oral plant‐derived lipid nanoparticles delivering CRISPR–Cas9 targeting epigenetic modifiers have achieved efficient macrophage‐specific gene editing in vivo, enabling programmable regulation of immunosuppressive gene expression without genome wide off target effects [510].

In cancer, TAMs secrete a plethora of immunosuppressive cytokines that dampen antitumor immunity. TGF‐β is a critical immunosuppressive factor. TAM‐derived TGF‐β inhibits CD8^+^ T cell cytotoxicity [555], promotes Treg differentiation [556], and induces fibroblast activation [557]. TGF‐β blocking antibodies and receptor kinase inhibitors (e.g., galunisertib) have demonstrated enhanced antitumor immunity and reduced metastasis in preclinical models [525], but clinical translation has been constrained by cardiac valve toxicity and autoimmunity [558]. IL‐10 is another key cytokine secreted by TAMs that suppresses dendritic cell maturation and T cell activation [559, 560], though its pleiotropic functions in maintaining immune homeostasis make direct blockade challenging. PGE2, produced by TAMs via COX‐2 upregulation, inhibits T cell proliferation and NK cell activity [561]; COX‐2 inhibitors have shown promise in preclinical hepatocellular carcinoma models [526]. Beyond soluble factors, TAMs express membrane‐bound immune checkpoint ligands including PD‐L1, VISTA, and B7‐H4, which directly engage inhibitory receptors on T cells. Unlike therapeutic antibodies targeting T cell checkpoints, strategies that block these ligands specifically on TAMs are emerging. For instance, anti‐VISTA antibody HMBD‐002 has entered clinical trials and is thought to reprogram TAMs toward an inflammatory phenotype [541, 542]. The CCL22–CCR4 axis, through which TAMs recruit Treg cells, represents another suppressor; blocking CCR4 reduces intratumoral Treg accumulation [562].

### 7.2 Clinical Challenges

Despite the remarkable progress in macrophage‐targeted therapies, several critical challenges impede their clinical translation. These challenges span therapeutic timing, delivery systems, and biomarker development, and are common to both sepsis and cancer, albeit with disease‐specific manifestations.

#### 7.2.1 Therapeutic Timing: The Window of Opportunity

The dual‐phase plasticity of macrophages presents a fundamental timing dilemma. In sepsis, macrophages transition from a hyperinflammatory state to an immunosuppressive state, each demanding opposite interventions. Preclinical studies have demonstrated that anti‐PD‐1/PD‐L1 blockade improves survival only when administered during the late immunoparalysis phase, whereas administration during the early hyperinflammatory phase may accelerate death [563]. Similarly, CCR2 blockade during early infection impairs pathogen clearance and paradoxically worsens outcomes [564]. This time‐dependent window necessitates precise disease staging using biomarkers such as mHLA‐DR and ferritin, as successfully implemented in the ImmunoSep trial [535].

In cancer, the timing challenge manifests differently. The education of TAMs is a chronic, progressive process. Early‐stage tumors have relatively low TAM infiltration and less profound immunosuppression, whereas advanced tumors harbor heavily reprogrammed, epigenetically locked TAMs resistant to reprogramming. The optimal timing for TAM‐targeted interventions likely varies across tumor types and stages, yet current clinical trials rarely stratify by these parameters [565].

#### 7.2.2 Delivery System Limitations

Effective delivery of therapeutic agents to macrophages faces multiple barriers. In sepsis, the host is often hemodynamically unstable with compromised organ perfusion. Systemic delivery risks unintended immunosuppression, increasing susceptibility to secondary infections [566]. Localized delivery strategies, such as CRV peptide‐modified lipid nanoparticles for in situ CAR‐M programming targeting MRSA, offer promise but remain at the preclinical stage [491].

In cancer, solid tumors present dense stromal barriers, abnormal vasculature, and elevated interstitial fluid pressure, all impeding efficient drug penetration [567]. TAMs are often located in hypoxic, poorly perfused tumor regions, further limiting drug access [363]. While nanoparticles can leverage the EPR effect for passive targeting, active targeting strategies using ligands such as mannose (for CD206) or folate (for FRβ) have shown improved specificity [568]. However, the heterogeneity of TAM phenotypes across and within tumors complicates universal targeting. Additionally, systemic administration of TAM‐modulating agents may cause on‐target off‐tumor effects, exemplified by the anemia and thrombocytopenia observed with CD47 blockade due to CD47 expression on erythrocytes and platelets [569].

#### 7.2.3 Biomarker Gaps for Patient Stratification

The success of macrophage‐targeted therapies depends critically on identifying patients most likely to respond. In sepsis, the ImmunoSep trial demonstrated that stratifying patients by ferritin (>4420 ng/mL) and mHLA‐DR (<5000 receptors/cell) successfully identified those who would benefit from anakinra or IFN‐γ therapy [535]. However, these biomarkers are not universally available, and their cutoffs require validation across diverse populations.

In cancer, reliable biomarkers for TAM‐targeted therapies are lacking. While high TAM infiltration often correlates with poor prognosis and immunotherapy resistance [570], it does not reliably predict response to CSF‐1R inhibitors or CD47 blockers [571]. Candidate biomarkers under investigation include TAM subset signatures (TREM2^+^, SPP1^+^) [572, 573], serum CSF‐1 levels [574], and tumor CD47 expression [575], but none have been prospectively validated. The failure of magrolimab in Phase III trials was partly attributed to the lack of patient selection based on CD47 expression or TAM functional status [576]. Biomarkers distinguishing patients who would benefit from TAM depletion versus reprogramming versus checkpoint blockade are urgently needed.

#### 7.2.4 Looking Forward: Integration and Solutions

Addressing these challenges requires several coordinated efforts. First, the development of point‐of‐care dynamic biomarkers capable of real‐time immune monitoring will enable timely therapeutic intervention. Second, smart delivery systems that respond to microenvironmental cues (pH, enzymes, hypoxia) could achieve on‐demand drug release at target sites. Third, modular combination strategies that simultaneously target multiple nodes of the macrophage life cycle may overcome resistance. Finally, cross‐disease borrowing of successful paradigms, such as the ImmunoSep trial's phenotype‐to‐treatment matching approach, could accelerate progress in oncology. The future of macrophage‐targeted therapy lies in precision immunomodulation guided by biomarkers, enabled by advanced delivery platforms, and optimized by context‐dependent timing.

## 8 Conclusion and Future Perspectives

In this review, we began by tracing the ontogeny and tissue‐specific specialization of resident macrophages, establishing the physiological foundation for their remarkable phenotypic plasticity. We then examined two extreme pathological trajectories of macrophage dysfunction. In sepsis, macrophages transition from guardians of anti‐infectious immunity into drivers of cytokine storm and ultimately sink into immunoparalysis. In cancer, they are progressively educated by the tumor microenvironment into accomplices that suppress antitumor immunity, promote angiogenesis, facilitate metastasis, and support CSCs. Despite this functional opposition, profound mechanistic commonalities including metabolic reprogramming, epigenetic imprinting, and immune checkpoint upregulation converge across both diseases. This suggests that lethal infection can be viewed as an accelerated model of macrophage dysfunction, and that insights derived from one context can inform the other. Building upon these mechanistic understandings, we further reviewed current macrophage targeted therapeutic strategies, spanning blockade of recruitment, reprogramming of polarization, enhancement of phagocytosis, and inhibition of immunosuppressive signals, with ongoing clinical trials and emerging technologies including CAR‐M and epigenetic editing.

Despite these advances, several critical gaps remain. First, current understanding of macrophage heterogeneity largely relies on reductionist M1/M2 classifications that fail to capture the continuous spectrum of functional states revealed by single cell technologies. Second, the optimal therapeutic windows for macrophage targeted interventions in both sepsis and cancer remain poorly defined, contributing to mixed clinical trial outcomes. Third, reliable biomarkers for patient stratification are lacking, particularly in oncology, where TAM subset signatures await prospective validation. Fourth, delivery systems capable of achieving macrophage‐specific targeting without off tumor effects remain a significant technical hurdle, as exemplified by the on target hematological toxicity of CD47 blockade.

Addressing these gaps will require coordinated efforts across multiple fronts. The integration of single cell multi omics and spatial transcriptomics with clinical outcome data holds promise for establishing precision patient stratification systems based on macrophage functional states. At the therapeutic level, we propose a paradigm shift from functional blockade toward functional recalibration, re‐establishing the functional equilibrium of macrophages under specific pathological contexts. CAR‐M technology, metabolic modulators, and epigenetic editing provide unprecedented tools for achieving this goal. Finally, bidirectional cross disease borrowing represents a natural extension of mechanistic research toward clinical translation. Metabolic or epigenetic interventions validated in septic immunoparalysis may be applicable to TAM reprogramming, and immunotherapeutic advances from oncology may provide novel avenues for late‐stage sepsis. Realizing this vision will require sustained interdisciplinary collaboration across immunology, metabolism, epigenetics, and bioengineering.

## Author Contributions

L.Z., J.J., and Z.W. conceived the study, participated in coordination, and helped draft the manuscript. S.H. drafted the manuscript and substantively revised it and designed all the figures. J.F., W.L., D.L., R.W., Y.W., J.S., H.Z., Q.C., and Z.X. helped draw the figures. Z.X., F.X., and D.L. helped revise the manuscript. All authors have read and approved the final manuscript.

## Funding

This study was supported by the National Natural Science Foundation of China (82222038 and 81970259), the Outstanding Young Talents of National Defense Biotechnology (01‐SWKJYCJJ06), and the Military Clinical Key Specialty Construction Project, and Chongqing Outstanding Youth Fund (CSTB2022NSCQ‐JQX0017).

## Ethics Statement

The authors have nothing to report.

## Conflicts of Interest

The authors declare no conflicts of interest.

## Data Availability

Data sharing is not applicable to this article as no datasets were generated or analyzed during the current study.
